# Mapping the plasma proteomic architecture of systemic lupus erythematosus

**DOI:** 10.1172/jci.insight.206938

**Published:** 2026-06-02

**Authors:** Geoffrey H.D. Leung, Charlotte Bottomley, Norzawani Buang, Robert T. Maughan, Benjamin J. Whittle, Boroumand Zeidaabadi, Yun-Ju Huang, Tabitha Turner-Stokes, Marie Condon, Liz Lightstone, Tom Cairns, Marina Botto, Matthew C. Pickering, James E. Peters

**Affiliations:** 1 Department of Immunology and Inflammation, Imperial College London, London, United Kingdom.; 2 School of Medicine, Chang Gung University and Division of Rheumatology, Allergy and Immunology, Chang Gung Memorial Hospital, Taoyuan, Taiwan.; 3 Imperial Lupus Centre, Imperial College Healthcare NHS Trust, London, United Kingdom.

**Keywords:** Autoimmunity, Clinical Research, Inflammation, Lupus, Proteomics, Rheumatology

## Abstract

Systemic lupus erythematosus (SLE) is a heterogeneous systemic autoimmune disease, yet the molecular basis underlying this variability remains incompletely understood. We profiled the plasma proteome in 260 SLE patients and 86 healthy volunteers (HVs) using the SomaScan v4.1 platform, quantifying 7,288 analytes corresponding to 6,595 unique proteins. We identified 215 proteins that were robustly differentially abundant between SLE patients and HVs in both discovery (*n* = 207 SLE, *n* = 45 HVs) and validation sets (*n* = 53 SLE, *n* = 41 HVs). Within-cases analyses identified 421 proteins associated with disease activity. Network-based clustering delineated correlated protein modules, including an interferon-associated (IFN-associated) module and a kidney-associated module. Autoantibody-stratified analyses further uncovered distinct proteomic endotypes; positivity for antibodies targeting RNA-binding proteins (anti-Sm, anti–Ro-60, anti-RNP68, anti–RNP-A) was associated with increased IFN-stimulated protein levels (e.g., MX1, ISG15, and CXCL10), independent of disease activity. Anti-Sm, anti–RNP-A, and anti-Ro52 antibodies were associated with reduced plasma levels of their respective autoantigens. Anti-dsDNA antibodies were associated with elevated levels of CD40 ligand (CD40LG) and the neutrophil protease, proteinase-3. Moreover, we identified an association between CD40LG and disease activity specific to the anti-dsDNA–positive subgroup. Together, these data define plasma protein signatures of SLE and disease activity, highlight autoantibody-specific molecular phenotypes, and provide a basis for precision medicine.

## Abstract 2

Most comprehensive #proteomic map of #SLE to date (>6000 proteins) reveals potential therapeutic targets, biomarkers, and autoantibody-linked proteomic signatures providing a basis for #precision medicine

## Graphical abstract

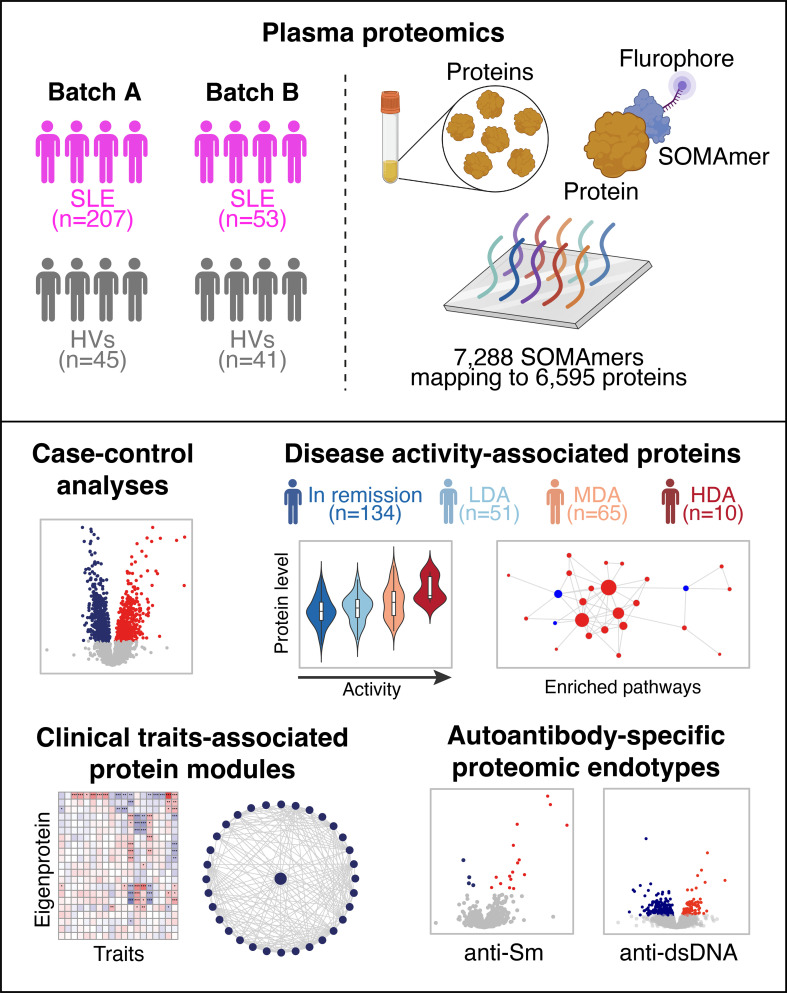

## Introduction

Systemic lupus erythematosus (SLE) is an autoimmune disease with complex pathogenesis involving dysregulation of both adaptive and innate immunity. A hallmark of SLE is the production of autoantibodies directed against nuclear antigens (anti-nuclear antibodies; ANAs). Established pathogenic pathways include autoreactive B cells, the complement system, and type I interferon (IFN) signaling, which lead to tissue inflammation and injury. Clinically, SLE is highly heterogeneous, ranging from mild to organ- or life-threatening disease, and with variable patterns of organ involvement. There is also interpatient variability in the targets of ANAs. This heterogeneity presents major challenges for patient management, drug development, and trial design. There is a pressing need for a precision medicine approach, matching the right therapy to the right patient. Our ability to deliver such a tailored therapeutic strategy is hampered by insufficient understanding of the molecular basis of heterogeneity and we lack biomarkers that can identify patient subgroups who would benefit from pharmacological targeting of specific pathways.

The advent of genomic technologies has enabled comprehensive assessment of various molecular domains in SLE such as the genome or transcriptome. Genome-wide association studies (GWAS) have elucidated genetic risk factors for SLE (1–3), while transcriptomic studies have revealed the dysregulated gene expression programs that occur once disease is established (4–6). A key finding from the latter was the identification of the IFN-stimulated gene (ISG) signature, a gene expression signature indicating stimulation by IFNs, particularly type I IFNs (4, 7–9). By contrast, few studies have investigated the proteome in SLE (10–12). Proteins are the effector molecules of biology and the targets of most drugs. In addition, circulating proteins (measurable in plasma or serum) can provide useful and tractable biomarkers suitable for measurement in clinical practice. Thus, evaluation of proteomic changes in SLE could be valuable both for understanding pathogenesis and for translational research (13). Advances in proteomic technologies now allow for highly multiplexed protein measurements. Here we performed plasma proteomic profiling using the SomaScan platform, measuring 7,288 protein analytes. Our data identify differentially abundant proteins in patients with SLE versus healthy volunteers (HVs), protein signatures of active disease, and modules of correlated proteins associated with clinical traits. Moreover, we identify autoantibody-specific proteomic endotypes, with distinct proteomic patterns correlated with anti-Smith (anti-Sm) and anti–double-stranded DNA (anti-dsDNA) autoantibody positivity. Intriguingly, we identify an association of disease activity and CD40LG that is specific to anti-dsDNA autoantibody–positive individuals. These findings provide a basis for precision medicine approaches in SLE.

## Results

### Plasma proteomic profiles of SLE.

We recruited 268 patients with SLE and 86 HVs. The cohort encompassed a wide range of disease duration and disease activity (Table 1). Positivity for autoantibodies was as follows: anti-dsDNA *n* = 70 patients (28%), anti-Sm *n* = 63 (24.6%), anti-Ro60 *n* = 120 (46.9%), anti-Ro52 *n* = 79 (30.1%), anti-La *n* = 31 (12.1%), anti–ribonucleoprotein 68 (anti–RNP-68) *n* = 36 (14.1%), and anti–RNP-A *n* = 68 (26.6%) (Table 1). Reduced complement C3 (<0.70 g/L) was observed in 14% of samples and reduced complement C4 (<0.16 g/L) in 26%. One hundred fifty-eight patients (61%) had a history of previous lupus nephritis (LN) (Supplemental Data File 1; supplemental material available online with this article; https://doi.org/10.1172/jci.insight.206938DS1), and 10 patients had recent LN (defined as blood sampling within 3 months of a renal biopsy showing LN). Proteomic measurements were performed in 2 batches. After quality control, 260 SLE and 86 HV samples were available for analysis (Supplemental Figure 1). Batch A (“discovery set”) consisted of 207 SLE samples and 45 HV samples and batch B (“validation set”) consisted of 53 SLE samples and 41 HV samples. We examined the effects of demographic variables on the proteome (Supplemental Material and Supplemental Figures 2 and 3). No global proteomic differences between male and female SLE patients or between male and female HVs were observed on PCA (Supplemental Figure 3).

**Figure 1:**
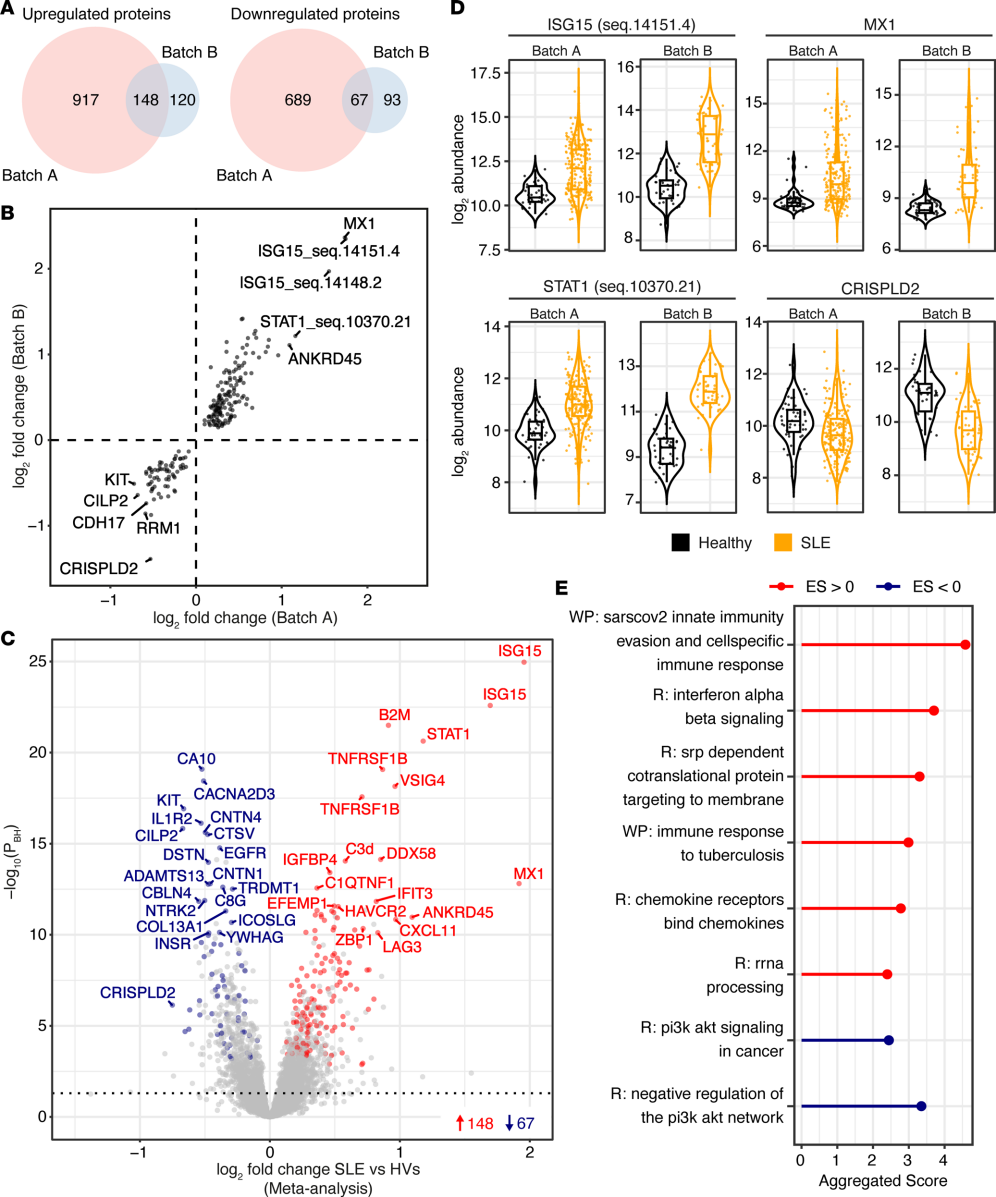
Differentially abundant proteins and enriched pathways in SLE versus HVs. (**A**) Venn diagrams of the number of significantly (FDR < 0.05) upregulated (left) and downregulated (right) proteins in SLE versus HVs in batch A (*n* = 207 SLE, *n* = 45 HVs) and B (*n* = 53 SLE, *n* = 41 HVs) data. (**B**) Comparison of effect size estimates (covariate-adjusted log_2_ fold change) for validated proteins between batch A and batch B. Each point represents the protein target of a SOMAmer; 215 protein targets that were significantly differentially abundant (FDR < 0.05) in both batch A and batch B are shown. The top 5 upregulated and downregulated protein targets (ranked by meta-analyzed effect sizes) are annotated. (**C**) Volcano plot showing the results from meta-analysis of batches A and B. Colored points represent protein targets replicated in both batches (FDR < 0.05). Red, upregulated; blue, downregulated; gray, not replicated or non-significant. (**D**) Examples of most significant differentially abundant proteins. Each point represents a sample. Orange, SLE; black, HVs. (**E**) Pathways enriched (FDR < 0.05) in both batches. Aggregated score was calculated using robust-rank aggregation. Red, upregulated pathways; blue, downregulated pathways. R, reactome; WP, WikiPathways; ES, effect size; *P*_BH_, Benjamini-Hochberg–corrected *P* value.

To identify proteomic signatures associated with SLE, we performed differential protein abundance testing of SLE versus HVs. For readability, where reporting the number of associations we use the term “protein” to refer to the protein target of each SOMAmer, but provide further detail of SOMAmer to protein mapping in Supplemental Data File 2. In batch A (*n* = 207 SLE, *n* = 45 HVs), we identified 1,821 proteins associated with SLE (1,065 upregulated, 756 downregulated; FDR < 0.05) (Figure 1A, Supplemental Figure 4A, and Supplemental Data File 3). In batch B (53 SLE, 41 HVs), we identified 428 differentially abundant proteins (268 upregulated, 160 downregulated; FDR < 0.05) (Supplemental Figure 4B and Supplemental Data File 4). To enhance robustness, SLE-associated proteins were only considered replicated if they were statistically significant (FDR < 0.05) independently in both batch A and batch B; a total of 215 proteins fulfilled this criterion (Figure 1A and Supplemental Data File 5). The estimated effect sizes for these replicated proteins were strongly correlated between the 2 batches (Pearson’s *r* 0.94) (Figure 1B). We then performed a meta-analysis of the 2 batches to obtain an overall estimate of the effect sizes for the replicated proteins (Figure 1C and Supplemental Data File 5). Notably, there was marked upregulation of IFN-stimulated proteins (ISPs) in SLE, including ISG15, MX1, STAT1, B2M, DDX58, IFIT3, and CXCL11 (Figure 1, C and D). Other strongly upregulated proteins included TNFRSF1B (TNF-R2), VSIG4 (the receptor for complement C3b and iC3b), and the complement fragment C3d. The most downregulated protein was CRISPLD2, a secreted LPS-binding glycoprotein that dampens TLR4-mediated cytokine responses (14). To evaluate whether disease chronicity or treatment impacted our results, we performed sensitivity analyses limiting to samples taken within 5 years of SLE diagnoses and excluding patients receiving particular treatments. The findings from these analyses were consistent with the primary analysis (Supplemental Figure 5). Gene set enrichment analysis (GSEA) identified 8 pathways that were significantly enriched in both batches, including multiple terms reflecting IFN signaling, as well as SRP-mediated cotranslational protein targeting, and downregulated PI3K/AKT signaling (Figure 1E and Supplemental Data File 6).

### Plasma proteomic signatures of lupus disease activity.

**Figure 2:**
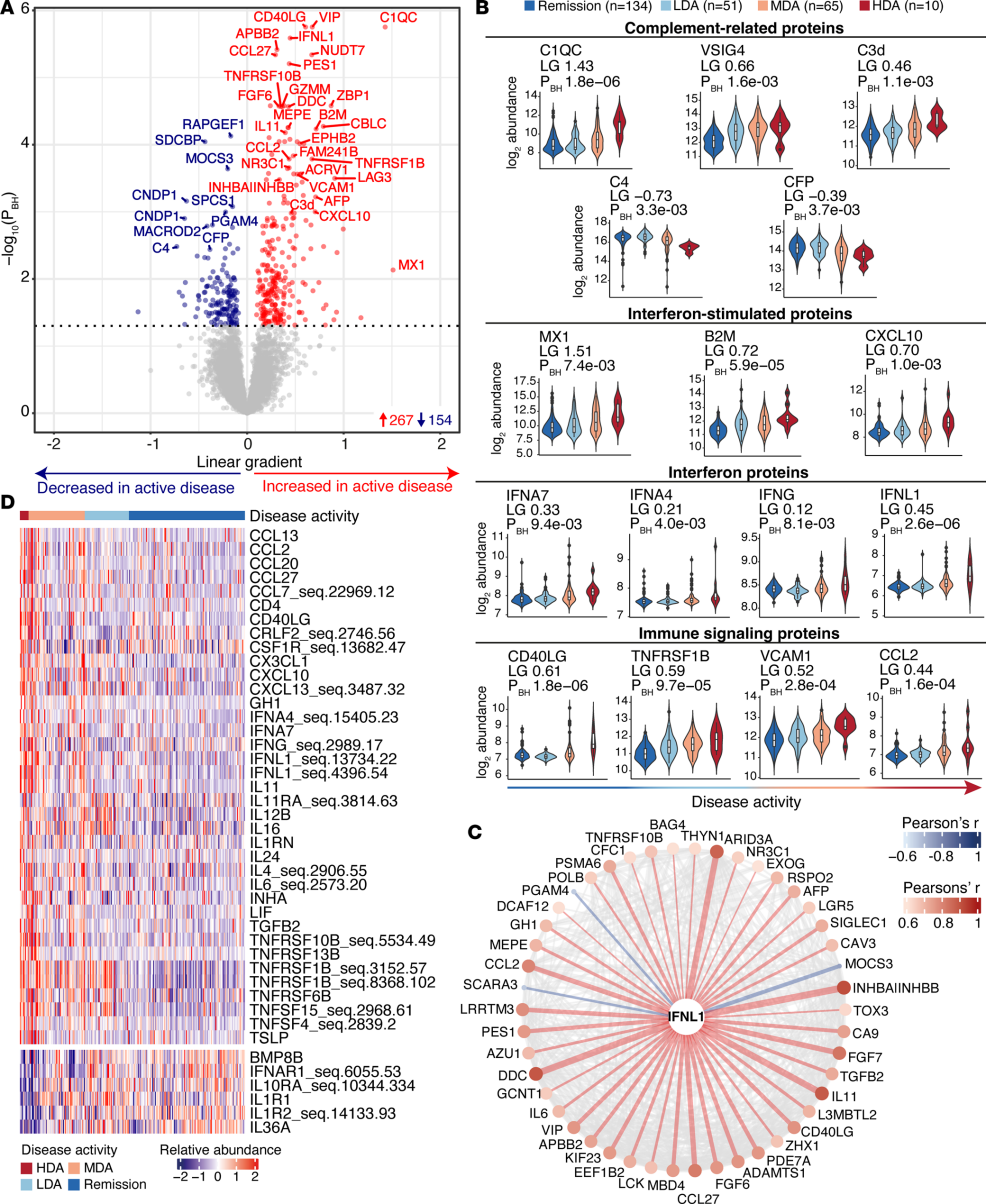
Proteins associated with SLE disease activity. *n* = 260 SLE samples. (**A**) Volcano plot. Linear gradient shows the magnitude and direction of change in the protein level with increasing disease activity. Dotted line = 5% FDR. Each point represents a protein. Red, significant positive association with active disease; blue, negative association; gray, non-significant. (**B**) Examples of disease activity–associated complement-related, IFN pathway, and immune signaling proteins. LG, linear gradient. Unique SOMAmer identifiers for displayed proteins where the SomaScan has more than 1 SOMAmer are B2M (seq.3485.28), IFN-α4 (IFNA4, seq.15405.23), IFN-γ (IFNG, seq.2989.17), TNFRSF1B (seq.3152.57), and VCAM1 (seq.2967.8). (**C**) Disease activity–associated proteins correlated with IFNL1. For clarity of visualization, only the proteins associated with disease activity at 1% FDR are displayed. A detailed list of proteins correlated with IFNL1 is provided in Supplemental Data 9. Edges represent pairwise protein correlations with |Pearson’s *r*| > 0.6. Node size, color, and edge thickness indicate the strength of correlation (Pearson’s *r*) with IFNL1. Edge color denotes correlation direction: blue for negative and red for positive. (**D**) Heatmap of significant (FDR < 0.05) disease activity–associated proteins in the KEGG “cytokine-cytokine receptor interaction” pathway. Protein levels were adjusted for sex and batch. *P*_BH_, Benjamini-Hochberg–corrected *P* value.

Samples were obtained from patients with a range of disease activities; 134 patients were in clinical remission, 51 had low disease activity (LDA), 65 had moderate disease activity (MDA), and 10 had high disease activity (HDA) (definitions in Methods). We tested for proteomic associations with SLE disease activity coded as an ordinal variable. To increase power, we analyzed both batch A and B together, adjusting for batch; 421 proteins were significantly associated (FDR <0.05) with disease activity (Figure 2A and Supplemental Data File 7). Comparison of results using an alternative approach for batch correction (ComBat) (15) yielded highly consistent results (Supplemental Material and Supplemental Figure 6). As a sensitivity analysis to explore the impact of different ways of encoding disease activity, we performed Spearman’s correlation analysis between SLE Disease Activity Index (SLEDAI) (as a numeric variable) and each protein, which showed similar results to the primary analysis (Supplemental Figure 7). Sensitivity analyses exploring the impact of disease duration and treatment produced results consistent with the primary analysis (Supplemental Figure 8). For 3 patients, longitudinal samples taken at 4 or more time points were available, which we used to qualitatively explore selected proteins that we had identified as associated with disease activity in the cross-sectional analysis (Supplemental Material and Supplemental Figure 9).

GSEA revealed significant enrichment of 90 pathways (Supplemental Data File 8), the most significant of which were “immunoregulatory interactions between a lymphoid and a non-lymphoid cell” and “cytokine-cytokine receptor interaction.” The proteins most strongly associated with disease activity by Benjamini-Hochberg–corrected *P* value (*P*_BH_) were CD40LG, vasoactive intestinal peptide (VIP), and C1QC (all *P*_BH_ 1.77 × 10^–6^). Active disease was also associated with other complement-related proteins, including increased C3d and VSIG4 and decreased intact C4 and CFP (properdin) (Figure 2B). ISPs (MX1, B2M, and CXCL10) were positively associated with disease activity, with MX1 showing the greatest effect size. Levels of type I, II, and III IFNs were also associated with active disease, with IFNL1 (a type III IFN) showing the strongest effect. Evaluation of connections between IFNL1 and other disease activity-associated proteins revealed IFNL1 was co-correlated (|Pearson’s *r*| > 0.6) with 75 other proteins (Figure 2C and Supplemental Data File 9).

The SomaScan includes many proteins that are primarily intracellular. Their detection in plasma may reflect cell death and turnover. While such proteins may be useful biomarkers of disease activity, some are likely to be downstream readouts of tissue inflammation and injury rather than upstream pathogenic drivers. In contrast, plasma proteins associated with disease activity that have a biological role in blood may be more promising therapeutic candidates. For example, cytokines and their receptors have proven to be important therapeutic targets in inflammatory diseases. We therefore extracted a list of proteins annotated in the KEGG “cytokine-cytokine receptor interaction” pathway and intersected this with the list of SLE activity–associated proteins (Figure 2D). In active SLE, there was upregulation of TNF superfamily and TNF receptor superfamily members (e.g., TNFSF4 [OX40L], TNFSF15 [TL1A], TNFRSF1B [TNFR2], and TNFRSF10B [DR5]), chemokines (e.g., CCL2, CCL27, CXCL10, and CXCL14), interleukins (e.g., IL-6, IL-11, and IL-24), and growth factors. In contrast, there was downregulation of antiinflammatory proteins, including IL-10RA and the decoy receptor IL-1R2.

### Associations with complement proteins.

Given the strong links between complement proteins and disease activity in the literature and in our data, we further analyzed proteomic associations with serum complement C3 and C4 measured in the clinical laboratory (*n* = 260 SLE patients). Both C3 and C4 were also included on the SomaScan assay. There was very strong concordance of protein measurements between the SomaScan and the clinical laboratory for intact C4 (Spearman’s ρ 0.95 in batch A, 0.91 in batch B), and moderately strong correlation for intact C3 (ρ 0.61 in batch A, 0.45 in batch B) (Supplemental Figure 10), providing validation of SomaScan measurements. In addition, various complement cleavage fragments were measured; correlations with intact C3 and C4 are presented in Supplemental Figures 11 and 12. Sixty protein targets were correlated with clinical laboratory measured C3 (|ρ| > 0.3, FDR < 0.05; Supplemental Data File 10), including 21 associated with disease activity (Supplemental Figures 13, A and B). Sixty-two proteins were significantly correlated with C4 (Supplemental Data File 11), 13 of which were disease activity associated (Supplemental Figure 10C and Supplemental Figure 13A). In total, 24 protein targets were associated with disease activity and levels of C3 and/or C4 (Supplemental Figure 13D). Proteins associated with higher disease activity and lower C3/C4 included CD40LG, ISPs (e.g., ISG15, IFIT3, and LAG3), and C1QC, while IL-36A, complement-related proteins (CFP [properdin], FCN1 [ficolin], and C2) and IFNAR1 showed the opposite relationships. The latter may reflect compensatory downregulation in the context of chronic IFN stimulation.

### Network analysis reveals protein modules associated with clinical phenotype.

**Figure 3:**
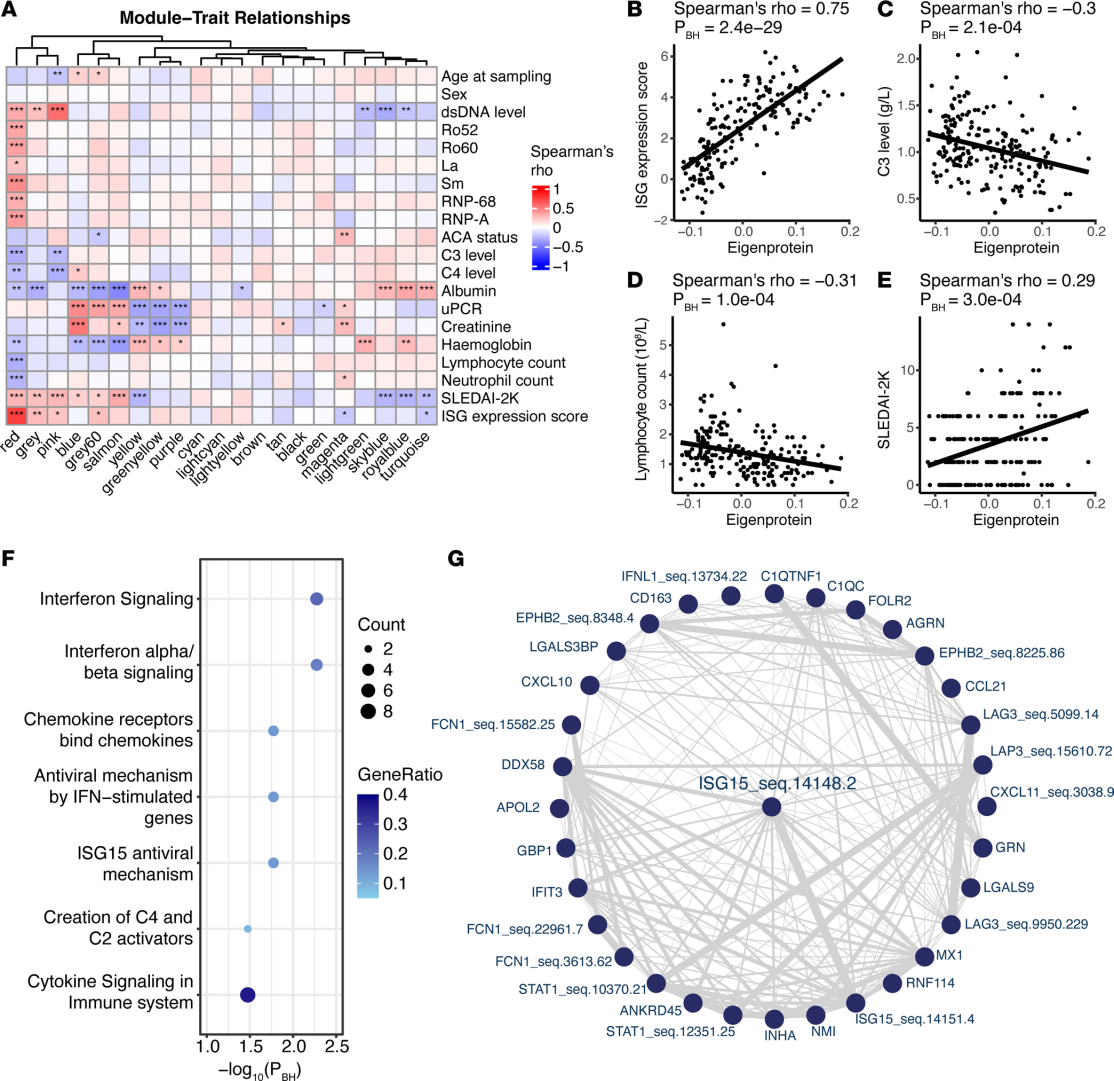
Network analysis identifies protein modules correlated with clinical traits. (**A**) Network constructed using SLE samples (*n* = 207). Correlation between each module and clinical traits. Asterisks represent Benjamini-Hochberg–adjusted *P* values (*P*_BH_) after correcting for 21 modules × 20 traits = 420 tests: *P_BH_ < 0.05, **P_BH_ < 0.01, ***P_BH_ < 0.001. Color gradient represents Spearman’s ρ. (**B**–**E**) Relationships between the eigenprotein values of the red module and clinical traits: (**B**) ISG expression score, (**C**) C3 level, (**D**) lymphocyte count, and (**E**) SLEDAI-2K. Each point represents a sample. Solid line indicates best fit from linear regression. (**F**) Pathways enriched for proteins in the red module. (**G**) Network of protein members of the red module. Edges represent strength of correlation between each pair of proteins. ISG15 (measured by SOMAmer seq.14148.2) was the module hub protein.

We used weighted gene coexpression network analysis (WGCNA) to construct a protein-protein correlation network, identifying 21 protein modules. We then tested for associations between each module and traits, including SLEDAI-2K score, ISG score (measured by quantitative PCR, qPCR), and clinical laboratory parameters. The red module, consisting of 35 proteins, was significantly associated with multiple clinical traits. It was positively correlated with SLEDAI-2K score and multiple autoantibodies and negatively correlated with neutrophil count, lymphocyte count, hemoglobin, albumin, and complement C3 and C4 (Figure 3, A–E, and Supplemental Figure 14). Notably, this module showed the strongest correlation with ISG score (ρ 0.75, *P*_BH_ 2.4 × 10^–29^; Figure 3B), and pathway analysis showed enrichment for IFN signaling pathways (Figure 3F). ISG15 (measured by SOMAmer seq.14148.2) was the hub protein in this IFN-associated module (Figure 3G and Supplemental Data File 12). ISG15 is an ISP and the module contained other ISPs (including CXCL10, CXCL11, DDX58, MX1, STAT1, APOL2, and LAG3) and also IFNL1.

**Figure 4:**
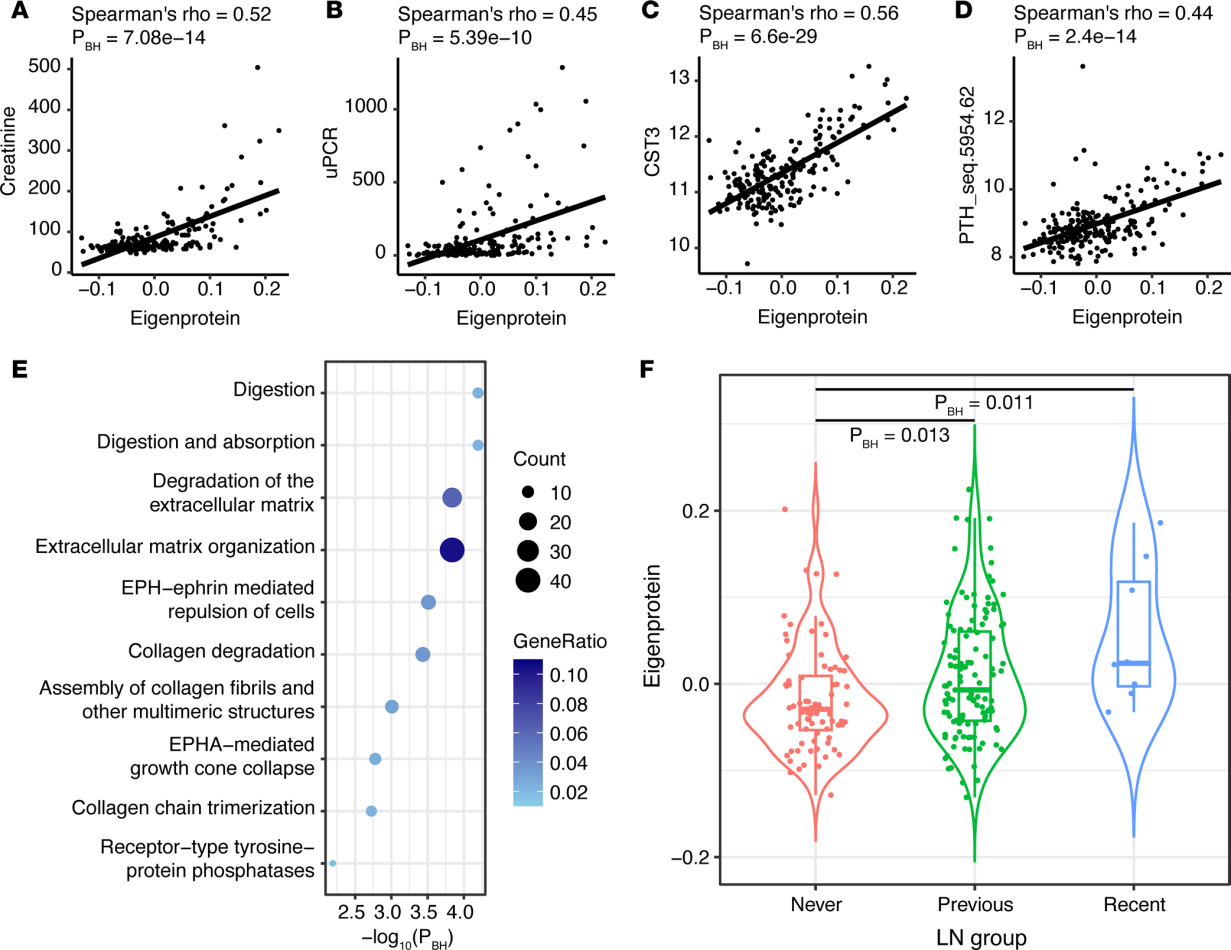
Kidney-associated protein module. (**A**–**D**) Relationships between the eigenprotein values of the blue module and renal markers: (**A**) creatinine, (**B**) urinary protein/creatinine ratio (uPCR), (**C**) cystatin C (CST3), and (**D**) parathyroid hormone (PTH). Each point represents an SLE sample. Solid line represents the line of best fit from linear regression. (**E**) Pathways enriched for proteins involved in the blue module. (**F**) Eigenprotein values of the blue module according to lupus nephritis (LN) status. *n* = 207 SLE samples. *P*_BH_, Benjamini-Hochberg–corrected *P* value.

The blue module, which had the second-highest number of clinical associations, was positively correlated with creatinine (ρ 0.52, *P*_BH_ 7.1 × 10^–14^) and proteinuria (ρ 0.45, *P*_BH_ 5.4 × 10^–10^) and negatively correlated with albumin (Figure 3A and Figure 4, A and B), indicating a link to renal dysfunction. Consistent with this, the module contained cystatin C (CST3, an accurate marker of glomerular filtration rate; Figure 4C), osteopontin (SPP1, a biomarker of kidney injury), and parathyroid hormone (PTH, which is dysregulated in renal impairment) (Figure 4, C and D, and Supplemental Data 13). Other kidney-related proteins included Ephrin and Ephrin receptors, which are associated with glomerular podocyte survival, angiogenic modeling, and responses to renal injury (16, 17). Comprising 611 proteins, this kidney-associated module was enriched for extracellular matrix–related (ECM-related) pathways, possibly reflecting renal scarring (Figure 4E). Moreover, the module eigenprotein was significantly higher in patients with a history of LN, particularly those with recent LN (defined as a renal biopsy showing active LN within 3 months of the blood sample), compared with those who never had LN (Figure 4F).

To systematically evaluate all SOMAmers for association with renal parameters, we performed univariate testing versus serum creatinine and urinary protein/creatinine ratio (uPCR). Four hundred forty-six proteins were associated with creatinine and 507 with uPCR (defined as *P*_BH_ < 0.05 and |ρ| > 0.3). Two hundred eighty-three proteins were associated with both creatinine and uPCR, and of these, 252 were members of the blue module identified in WGCNA (Supplemental Figure 15).

The red (IFN-associated) module was independent of the blue (kidney-associated) module (*r* –0.08) and not significantly associated with serum creatinine or uPCR. Given the cross-sectional nature and chronicity of disease in our study (such that both renal scarring and/or active nephritis could affect creatinine and uPCR), we then compared the red module eigenprotein values in patients who had never had LN, those with previous LN, and those with recent LN. There was no difference between the group who had never had LN and those with previous LN. However, individuals with recent LN had higher red module eigenprotein values (Supplemental Figure 16).

### Autoantibody-specific proteomic endotypes.

**Figure 5:**
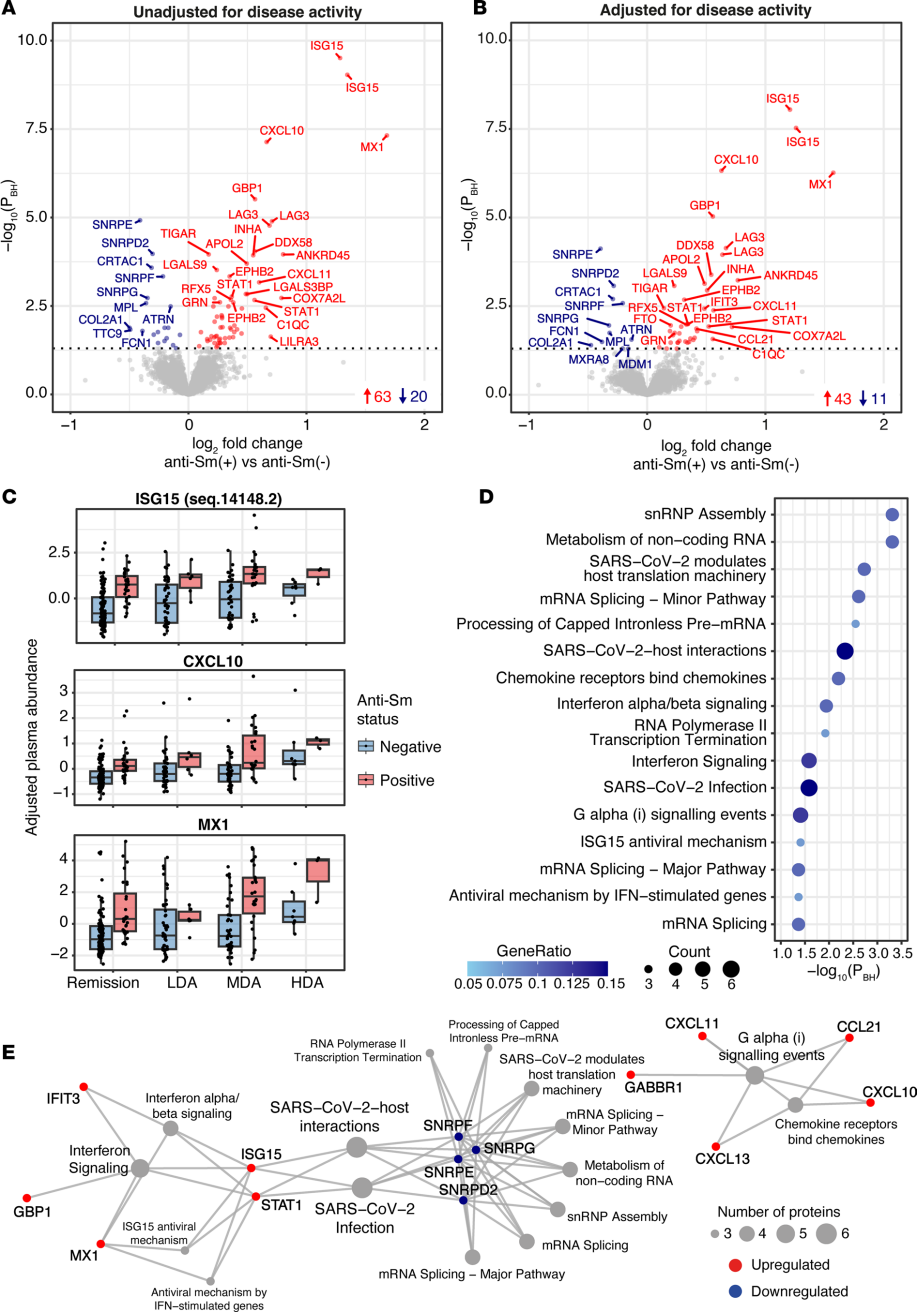
Proteins associated with anti-Sm autoantibodies. (**A**) Proteins differentially abundant in anti-Sm–positive (*n* = 63) versus anti-Sm–negative SLE (*n* = 193), without adjustment for disease activity. Each point represents a protein. Red, significantly upregulated; blue, significantly downregulated; gray, non-significant. (**B**) As for **A**, but after adjustment for disease activity. (**C**) IFN-stimulated proteins are associated with anti-Sm antibodies independent of disease activity. Displayed protein levels have been adjusted for batch and sex. (**D**) Significantly enriched pathways for proteins associated with anti-Sm antibodies after adjustment for disease activity. (**E**) Connections between proteins associated with anti-Sm antibody status independent of disease activity and the enriched pathways. Node size (for pathway terms) represents the number of proteins associated with the pathway. Red, upregulated proteins; blue, downregulated proteins. *P*_BH_, Benjamini-Hochberg–corrected *P* value.

Anti-Sm autoantibodies are highly specific to SLE and are associated with worse prognosis (18, 19). To identify a proteomic signature associated with anti-Sm autoantibodies, we compared the abundance levels of each protein between anti-Sm(+) (*n* = 63) and anti-Sm(–) (*n* = 193) patients. Eighty-three proteins were differentially abundant, with 63 proteins upregulated and 20 downregulated in the anti-Sm(+) samples (Figure 5A). We considered the possibility that disease activity might be confounded with anti-Sm autoantibody positivity, since patients with anti-Sm antibodies tend to have a worse prognosis and that some of the anti-Sm–associated proteins were associated with disease activity in our previous analyses. We therefore performed multiple linear regression, adjusting for disease activity. This revealed 54 proteins associated with anti-Sm antibodies independent of disease activity (43 proteins positively and 11 negatively correlated; Figure 5B and Supplemental Data File 14). Visualization of abundance of these proteins against anti-Sm status stratified by disease activity confirmed that the association with anti-Sm antibodies was independent of disease activity (Figure 5C and Supplemental Figure 17). Pathway enrichment analysis revealed anti-Sm–associated proteins were enriched for SARS-Cov-2–related, IFN signaling, small nuclear ribonucleoprotein (snRNP) assembly, and RNA processing pathway terms (Figure 5D). These pathway enrichment terms reflected increases in ISPs and reductions in snRNPs (Figure 5E). ISPs increased in anti-Sm(+) SLE included ISG15 (SOMAmer seq.14148.2), MX1, CXCL10, GBP1, DDX58, CXCL11, APOL2, STAT1, LGALS9, EPHB2, and LAG3. Since snRNPs are the targets of anti-Sm antibodies, it is possible that the reduction in circulating snRNPs reflects autoantibody-mediated depletion. Sensitivity analyses exploring the effects of disease duration and treatment confirmed that the finding of upregulated ISPs and downregulated snRNPs in anti-Sm(+) SLE was robust (Supplemental Figure 18, A–C). Finally, these associations remained significant after adjustment for anti-dsDNA antibody status in multivariable regression (Supplemental Figure 18D).

**Figure 6:**
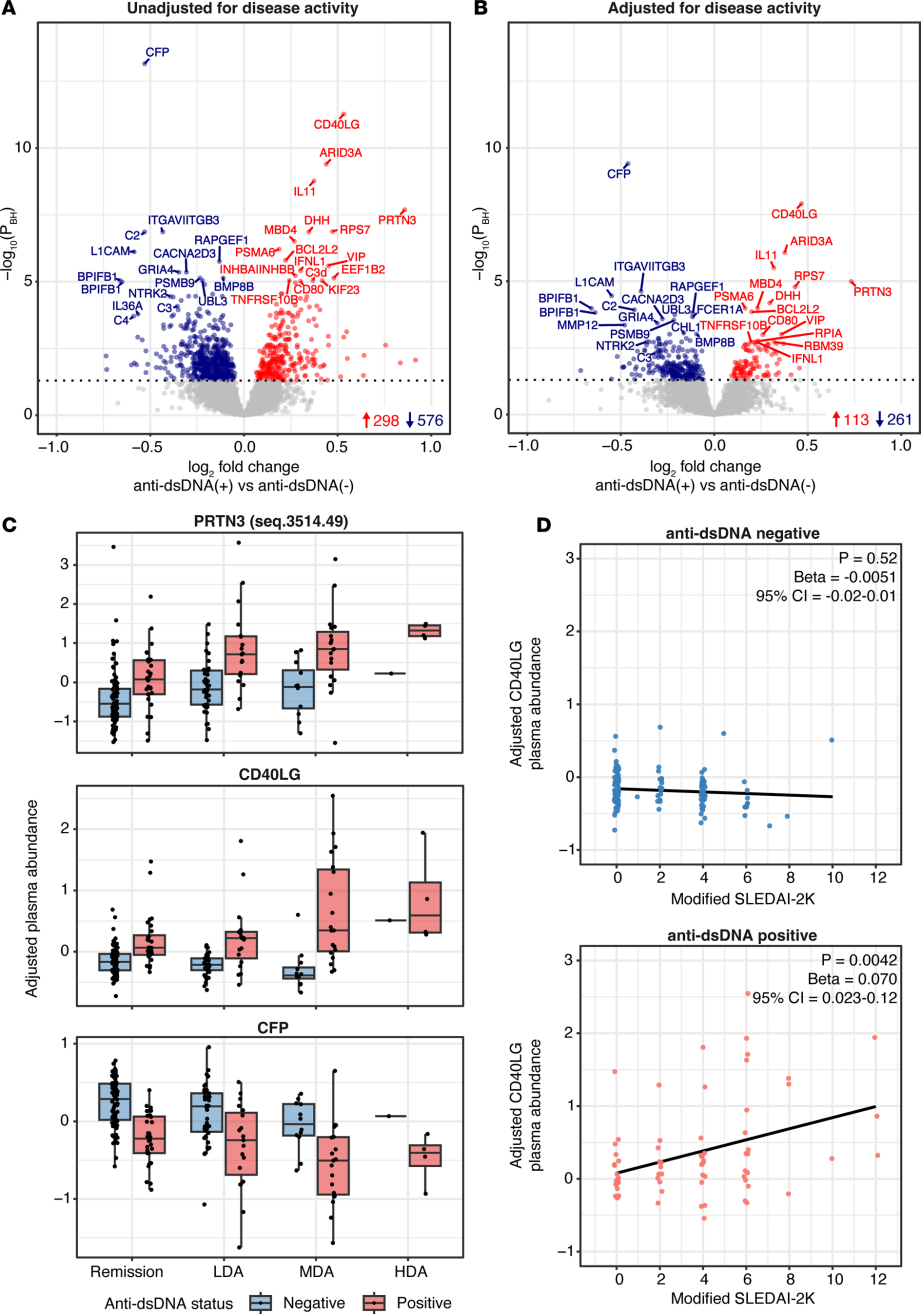
Proteins associated with anti-dsDNA autoantibodies. (**A**) Proteins differentially abundant in anti-dsDNA–positive (*n* = 70) versus anti-dsDNA–negative (*n* = 140) SLE samples, without adjustment for disease activity. Each point represents a protein. Red, significantly upregulated; blue, significantly downregulated; gray, nonsignificant. (**B**) As for **A**, but after adjustment for disease activity. (**C**) Examples of proteins associated with anti-dsDNA antibodies independent of disease activity. Protein levels in anti-dsDNA antibody–positive and –negative samples are shown stratified by disease activity. (**D**) The association of plasma CD40LG with disease activity is restricted to anti-dsDNA–positive individuals. SLEDAI-2K modified to exclude anti-dsDNA antibody status from the score. Each point represents an SLE sample. Solid line represents the line of best fit from linear regression. Protein levels in **C** and **D** have been adjusted for batch and sex. *P*_BH_, Benjamini-Hochberg–corrected *P* value; CI, confidence interval.

We then analyzed the associations between proteins and anti-dsDNA antibodies. Eight hundred seventy-four proteins were associated with anti-dsDNA antibody positivity (Figure 6A). After adjusting for a modified disease activity score (which excluded anti-dsDNA antibody status), 374 proteins remained associated with anti-dsDNA antibody status (Figure 6, B and C, and Supplemental Data File 15). The most upregulated proteins included CD40LG (CD40 ligand), a costimulatory molecule that activates B and T cells and promotes inflammatory responses (20), ARID3A (a DNA-binding protein), IL-11, the neutrophil protease proteinase-3 (PRTN3), and RPS7. Proteins reduced in anti-dsDNA antibody–positive SLE included CFP, ITGAV|ITGB3 (the vitronectin receptor, an integrin heterodimer), L1CAM, BPIFB1, and C2. Unlike for anti-Sm autoantibody status, the association of canonical ISPs with anti-dsDNA antibody positivity became non-significant after adjustment for disease activity. After adjusting for anti-Sm antibody status, associations of ISPs with anti-dsDNA positivity were attenuated (Supplemental Figure 19A). As a sensitivity analysis, Spearman’s rank correlation was performed to test for correlations between the levels of anti-dsDNA autoantibodies (as a continuous trait) and each protein (Supplemental Figure 20). CD40LG, PRTN3, and ARID3A were again among the strongest associations. Additional sensitivity analyses studying the effects of disease duration and treatment also recapitulated these strongest associations (Supplemental Figure 19, B–D). Intriguingly, visualization of CD40LG’s relationship with disease activity stratified by antibody status showed this association was restricted to the anti-dsDNA antibody–positive subgroup (Figure 6D; anti-dsDNA–positive: *P* 4.2 × 10^–3^, β 0.07; anti-dsDNA–negative: *P* 0.52, β –0.005). Formal interaction analysis confirmed this effect (*P* 6.7 × 10^–5^).

We also tested for proteomic associations with other autoantibodies against extractable nuclear antigens (ENAs) (Ro-52, Ro-60, La, RNP-A, and RNP-68). As for Sm, positivity for anti–RNP-68, anti–RNP-A, and anti–Ro-60 antibodies, but not anti–Ro-52 or anti-La, was associated with upregulated ISPs, even after adjustment for disease activity (Supplemental Figures 21–24). Antibodies against RNP-A were, like anti-Sm antibodies, associated with reduced circulating snRNPs. Anti–Ro-52 positivity was associated with reduction in circulating Ro-52 (TRIM21) and increased TNFSF13B (BAFF). Anti–Ro-60 and -La antibodies were also associated with reduced plasma Ro-52 in univariate analysis, but this association was abrogated after adjusting for anti–Ro-52 antibody status in multivariable analysis (Supplemental Figure 22C and Supplemental Figure 23C), indicating that it was being driven by concomitant anti–Ro-52 antibody positivity in patients with multiple autoantibody positivity. Finally, we found no proteomic associations with anti-phospholipid antibodies.

### Comparison with anifrolumab-induced proteomic changes in TULIP-1 trial.

Given the prominence of IFN pathways in proteomic signatures in our data, we examined to what extent these signatures might be reversible with anti–type I IFN receptor therapy through comparison with an external dataset from the study of Baker et al., which analyzed 169 proteins in SLE before and after anifrolumab in the TULIP-1 trial (21) using Olink and Simoa immunoassays. One hundred fifty-eight of these proteins were measured in our SomaScan dataset (reflecting 228 SOMAmers since some proteins were measured by multiple SOMAmers). Across these 158 proteins, log_2_ fold changes from our SLE versus HV comparison were negatively correlated with anifrolumab-versus-placebo changes at week 52 in TULIP-1 (*r* –0.43; Supplemental Figure 25A). Similarly, protein associations with disease activity in our study were inversely correlated with changes following anifrolumab treatment (*r* –0.52; Supplemental Figure 25B). Of the proteins measured in both studies, 30 were associated with disease activity in our study, with 13 significantly modulated by anifrolumab treatment. These data underscore the centrality of the IFN response in plasma proteomic signatures of SLE. However, some disease activity–associated proteins were not modulated by anifrolumab, including CD40LG, suggesting IFN-independent disease-activity related pathways (Supplemental Figure 25C). A caveat is interstudy differences, particularly the proteomic platforms used.

## Discussion

We performed wide-angled proteomic profiling in SLE, measuring 7,288 analytes reflecting 6,595 unique proteins, representing the most comprehensive assessment of the plasma proteome to date. Proteins differentially abundant in SLE versus HVs and proteins associated with disease activity reflected known pathogenic pathways in SLE, including dysregulation of adaptive immunity, complement pathways, and IFN and TLR signaling, validating the utility of plasma proteomics in uncovering disease mechanisms.

Notably, there was strong upregulation of protein products of the ISG signature in SLE and this was more pronounced in active disease. The lupus ISG signature was first described in transcriptomic microarray studies of immune cells (whole blood or PBMCs) (4, 7, 22). Here, we demonstrate that a similar “ISP” signature is present at the protein level in the extracellular space in blood. Few broad-capture plasma proteomic studies have been conducted in SLE. One prior study, which used an earlier SomaScan version (1,129 proteins), identified 4 ISPs (EPHB2, LAG3, CXCL13, and CXCL10) associated with SLE (10). In our study, all of these proteins were significantly upregulated in SLE samples and with increasing disease activity, validating previous findings. An analysis by Yang et al. of UK Biobank proteomic data generated on a different proteomic platform (Olink Explore) identified CXCL10 as a predictor of future incident SLE (23). This indicates that dysregulation of ISPs occurs prior to clinically overt disease. There are several caveats to the study by Yang et al.: (i) individuals were all more than 50 years old at study entry and developed unusually late-onset SLE, and (ii) diagnosis relied on UK Biobank codings based on routine healthcare administrative data and not formal classification criteria for SLE. A high ISG score is associated with worse prognosis (9), but has not entered clinical practice. A plasma-based ISP score rather than a gene expression assay might prove more tractable for clinical translation for several reasons. First, proteins are more robust to sample handling than RNA, which rapidly degrades at room temperature. Second, an ISP score would be more readily integrated into clinical workflows since blood-based protein biomarkers (e.g., C-reactive protein, troponin, and autoantibodies) are measured routinely in clinical biochemistry laboratories. Finally, a protein-based assay is likely to be less costly than a gene expression assay.

Among the most upregulated ISPs in the present study were ISG15, MX1, STAT1, CXCL11, B2M, and LAG3. LAG3 is an inhibitory receptor expressed on activated T cells. Its expression is increased in the context of chronic antigen exposure (e.g., cancer and chronic viral infection) and leads to T cell exhaustion (24–26). This may represent a compensatory mechanism to limit immune-mediated tissue damage. Our comparison to proteomic data from the TULIP-1 trial showed that LAG3 was downregulated by anifrolumab. In addition to elevated ISPs, we also observed upregulation of IFN proteins themselves. Notably, IFNL1, a type III IFN, was strongly upregulated in both comparison with HVs and the analysis of disease activity. IFNL has received less attention than type I IFNs in lupus pathogenesis, but its upregulation in SLE has been previously reported (27–31). In our exploratory longitudinal analysis, there was some correlation between ISPs and SLEDAI, but ISPs failed to fully normalize despite improvements in disease activity, suggesting persistent IFN pathway activation, in line with longitudinal studies of the ISG signature (32, 33). However, the number of patients for whom we had serial data was very small and so this needs to be interpreted with caution.

The IFN pathway protein module was not correlated with renal function or urinary protein leak, or with previous nephritis, but was elevated in patients with recent nephritis. Monogenic interferonopathies are characterized by skin, nervous system, and vascular involvement, while renal involvement is rare (34). However, higher ISG expression is associated with increased LN risk (35). A randomized trial of anifrolumab in LN (TULIP-LN) did not achieve its primary endpoint, but lupus clinical trial design is challenging and there was a trend towards more favorable secondary endpoints in the anifrolumab group (36, 37). Larger trials such as the ongoing phase III IRIS trial (ClinicalTrials.gov NCT05138133) should help clarify the relative importance of the IFN pathway in LN.

Our SomaScan results recapitulated historical studies of the complement pathway proteins beyond intact C3 and C4 (which are currently used in clinical practice as biomarkers) (38). We observed positive association of C3d and negative association of CFP (properdin) with active disease, in keeping with previous reports (39–42). C1Qc was one of the most strongly associated proteins with active disease. A previous study reported that C1Q negatively regulates type I IFN production by promoting preferential binding of immune complexes (ICs) to monocytes and thus reducing IC activation of plasmacytoid DCs, which are a major producer of type I IFNs (43). However, we observed a strong positive correlation between C1Qc and levels of ISPs. Our data are consistent with the observation of reductions in whole-blood *C1Qc* gene expression following anifrolumab therapy (21).

Disease activity–associated proteins included several members of the TNF superfamily of cytokines and receptors. One example was TNFSF4 (OX40L), whose plasma levels increased in more active disease. The *TNFSF4* gene locus is a susceptibility locus for SLE, pointing to a causal role in SLE aetiology (44). In keeping with this, conditional knockout of B cell OX40L ameliorates mouse models of SLE (45). Our data add to these lines of evidence, suggesting that OX40L is a potential therapeutic target in SLE. Further support for an upstream role of OX40L in SLE pathogenesis comes from the analysis of UK Biobank proteomic data by Yang et al., which identified upregulation of OX40L as a predictor of incident SLE (23). Another example was TNFSF15 (TL1A), which is a promising therapeutic target in inflammatory bowel disease (46). Upregulated TNFSF receptors included TNFRSF1B (TNF-R2), which acts as a receptor for both TNF and lymphotoxin α (LTA), and TNFRSF10B, the receptor for the apoptosis-inducing cytokine TRAIL. Interleukins increased in active disease included IL-6, IL-11, and IL-24. Conversely, there were negative correlations between disease activity and the antiinflammatory proteins IL-1R2 and IL-10RA. IL-1R2 is a decoy receptor for IL-1, and reduced IL-1R2 would be expected to increase proinflammatory IL-1 signaling. IL-10RA is a receptor for IL-10, an important antiinflammatory cytokine. Genetic variants in the *IL10RA* gene are associated with early-onset inflammatory bowel disease (47). Our findings raise the possibility that insufficient activation of homeostatic mechanisms contribute to increased disease activity, although since our data are observational, we cannot exclude the possibility that reductions in antiinflammatory proteins are secondary to active disease rather than contributors to it.

Coexpression network analysis identified modules of correlated proteins. Two modules had notable associations with clinical and laboratory parameters. One module correlated with ISG score and multiple autoantibodies, consistent with previous reports linking high ISG expression to autoantibodies against Ro, U1-RNP, Sm, and dsDNA (48, 49). Its hub protein, ISG15, bridges the type I and type II IFN pathways; induced by type I IFNs, it provides negative feedback to downregulate type I responses while promoting IFN-γ (50). A previous study identified ISG15-secreting plasmablasts and plasma cells in active SLE (51). The second protein module reflected renal function and other kidney-related physiology. This module contained over 600 proteins, potentially reflecting several distinct processes. These include (i) upstream drivers of renal injury, (ii) consequences of renal injury, including scarring/remodeling, (iii) direct effects of loss of renal function in terms of reduced hemofiltration or loss of renal endocrine functions (e.g., we observed elevation of PTH, likely reflecting reduced renal hydroxylation of vitamin D), and (iv) indirect effects of renal impairment on the proteome (e.g., from hemodynamic changes causing endothelial activation). Pathway analysis showed enrichment for ECM- and fibrosis-related pathways, suggesting renal scarring was a prominent feature underpinning this module.

Serological profiles in SLE correlate with clinical phenotype and prognosis (18, 19). For example, anti-dsDNA antibodies are associated with renal disease and anti-Sm antibodies with poor prognosis. Here we show distinct proteomic signatures associated with specific lupus autoantibodies that persist after adjustment for the potential confounder of disease activity. The Sm antigen represents a group of snRNPs that are RNA-binding proteins and whose physiological role is in RNA processing. Anti-Sm–positive SLE showed increased ISPs and reduced circulating snRNPs. A similar association with increased ISPs was seen for antibodies against RNP-A, RNP-68, and Ro60, but not Ro52 and La. Our observation extends previous reports (48, 52, 53) showing the association between antibody positivity to ENAs, including Sm, and IFN-stimulated transcripts to the protein level. In contrast, the association of anti-dsDNA antibodies with ISPs was abrogated or attenuated after adjustment for disease activity or anti-Sm positivity, respectively. Other studies report variable associations of anti-dsDNA antibodies and the ISG signature, but even when observed this association tends to be weaker than for anti-Sm or anti-RNP antibodies (48, 53, 54). Intriguingly, like Sm, RNP-A, RNP-68, and Ro60 are RNA-binding proteins, whereas Ro-52 (an E3 ubiquitin ligase) is not. Of potential relevance, a study of mothers of children of neonatal lupus did not show an association between anti-Ro antibody titers and a cellular reporter of type I IFN activity, although the specificity for Ro-52 versus Ro-60 was not available (55). Our data suggest a specific pattern whereby autoantibodies against RNA-binding proteins, but not other nuclear antigens, are associated with an ISP signature. The exception to this trend was anti-La antibodies, but this may reflect lack of statistical power due to the smaller number of anti-La–positive patients in our cohort. Of note, while there was no association between anti-La antibodies and individual ISPs when each protein was analyzed separately, there was a weak but statistically significant association between the IFN-related module and anti-La positivity, perhaps reflecting the power gain from WGCNA.

While correlative data cannot distinguish whether the association between antibodies against RNA-binding proteins and the IFN pathway represents a causal relationship or the direction of such a relationship, in vitro experiments support the hypothesis that such antibodies are interferonogenic. When combined with dead cellular matter, lupus sera containing antibodies against RNA-binding proteins, but not anti-dsDNA antibodies, can induce IFN-α production in PBMCs (56). This is consistent with our observation that the associations of ISPs with anti-dsDNA antibodies was attenuated after adjustment for anti-Sm antibody status. The link between anti-RNP antibodies and IFN pathway activation raises the question of whether specific autoreactive B cells (e.g., anti-Sm positive) could be targeted directly for therapeutic benefit. Examples of such an approach might be antigen-specific Tregs or bispecific autoantigen–T cell engagers (BaiTEs).

The reduction in snRNPs that we observed in patients with anti-Sm and anti–RNP-A antibodies may reflect depletion by these antibodies targeting epitopes on snRNP complexes. Although the reductions in snRNPs were in plasma, this may proxy intracellular abundance, raising questions about anti-Sm antibody effects on cellular machinery. Similarly, we observed depletion of the autoantigen Ro-52 (TRIM21) in patients with antibodies against Ro-52. These findings across multiple autoantibody specificities suggest a general pattern whereby circulating autoantibodies are associated with reduced plasma concentrations of their corresponding autoantigens.

Anti-dsDNA–positive SLE was associated with elevation of the neutrophil protease PRTN3. This might reflect release of neutrophil contents via neutrophil extracellular traps (NETs), which are a well-established source of nuclear autoantigens in SLE. It is also possible that anti-dsDNA antibodies drive further NET formation and release of PRTN3.

Soluble CD40LG showed strong associations across multiple analyses, including correlations with disease activity, anti-dsDNA antibody status, and complement C3/C4 levels. Intriguingly, the association with disease activity was specific to individuals with anti-dsDNA antibodies. CD40LG/CD40 interaction drives B cell differentiation into IgG-secreting plasma cells, making it central to antibody responses. This is consistent with the associations with anti-dsDNA antibodies (both positivity and titer) and reductions in complement C3/C4 (reflecting complement consumption by immune complexes). Previous studies have identified increased expression of CD40 or CD40LG in immune cells and serum in a range of immune-mediated diseases, including SLE (57), and, in line with our data, Goules et al. found that CD40LG was elevated in the sera of SLE patients and associated with anti-dsDNA positivity and disease activity (58). Further support for a pathogenic role for the CD40 pathway in SLE comes from animal models; blocking the CD40-CD40LG interaction ameliorates lupus-like disease in NZB/SWR or NZB/NZW F1 mice (59, 60) and also reduces anti-dsDNA antibodies. There have been efforts to therapeutically target CD40LG in SLE and other autoimmune diseases. While first-generation anti-CD40LG monoclonal antibodies (mAbs) were abandoned due to thrombotic events (61), direct targeting of CD40 or newer generation anti-CD40LG mAbs bioengineered with inert or absent Fc tails circumvents this issue. A phase II trial of the anti-CD40 mAb BI655064 in LN failed to meet its primary endpoint, although post hoc analysis suggested potential benefits of higher doses (62). Evaluation of frexalimab, a bioengineered anti-CD40LG mAb, is ongoing (ClinicalTrials.gov NCT05039840). Notably, comparison of our data to proteomic data from the TULIP-1 study revealed that, unlike many of the proteins we found associated with disease activity, CD40LG was unaffected by anifrolumab. This suggests that CD40LG axis inhibition might be a complementary approach to anti-IFN therapy by tackling an orthogonal pathogenic pathway. Given the strong associations between CD40LG and anti-dsDNA antibody and complement levels, our data highlight that a stratified medicine approach for anti-CD40 pathway therapeutics might be beneficial. This concept has parallels to the path to approval of belimumab, which proved effective in SLE when used in serologically defined subsets of patients after initial trial failure (63).

Our study has limitations. Although our data were arbitrarily split into batches that we used as discovery and replication subcohorts for case versus control analysis, this was a single-center study lacking external validation. For within-cases analyses, we analyzed both subcohorts together to boost power and thus we did not have a hold-out set for validation. We observed preanalytical variation accentuating case versus control differences in batch B, likely related to the control samples (Supplemental Material). Although statistical adjustment for PC1 attenuated inflation of test statistics, this issue motivated us to require SLE-associated proteins to be statistically significant in both batch A and B. As expected, we also observed a batch effect related to differing proteomic runs, but statistical batch correction abrogated this effect, enabling us to perform within-cases analysis on data from both batches. Ancestry matching of controls to cases was imperfect, although there were no global effects of ancestry on the proteome observed on PCA, suggesting this is unlikely to have substantially impacted our findings. Statistical adjustment for ancestry was not feasible since ancestry categories were based on self-reported data and were multiple and heterogeneous, included mixed and “other” ancestry designations, with several ancestry categories containing only small numbers of participants. This makes regression-based covariate adjustment unreliable, particularly in the context of high-dimensional proteomic data. Patients were sampled opportunistically at various points in the disease course and not at predefined time points; while this enabled cross-sectional evaluation of markers of disease activity, disease duration, chronic damage, and past and current treatment were potential confounders. Although we performed various sensitivity analyses to address this, there remains the possibility of residual confounding. In addition to effects of treatment on the proteome, treatment allocation is non-random and is influenced by both disease severity and serology (e.g., in our practice, belimumab is usually restricted to those who are anti-dsDNA antibody positive or who have low complement C3 or C4). Future studies examining paired pre- and posttreatment samples will be informative. Our cohort were adults and predominantly female and most had chronic disease. It is uncertain whether these proteomic findings can be extrapolated to other groups such as pediatric or early-onset groups. We did not see obvious global differences in terms of male versus female lupus on PCA. Nevertheless, we did not have sufficient numbers to ascertain whether the proteomic signatures of disease activity and autoantibody status hold true in males. The sample size in the high disease activity group was small compared with the low disease activity and remission groups, which may have limited power and could potentially be a source of bias. We analyzed activity as an ordinal variable, which partly offsets this issue by capturing the overall linear relationship between protein levels and increasing activity. We did not have sufficient longitudinal samples to formally and systematically evaluate intra-individual changes in disease activity–related markers over time. Future studies using inception cohorts with serial sampling are needed to validate putative biomarkers of disease activity identified in the present study.

Interpretation of plasma proteomic measurements can be challenging. In addition to canonical soluble proteins, the SomaScan platform includes targets measuring intracellular and membrane-bound proteins. Their detection may represent release from dead cells, and thus they are more likely to be biomarkers of tissue injury than pathogenic drivers. However, some intracellular proteins might also contribute to pathogenesis as a source of autoantigenic material in a feedforward loop or by causing tissue injury (e.g., neutrophil proteases such as PRTN3). Where proteins exist in both membrane-bound and cleaved states, it is not always clear whether plasma proteomic assays are exclusively capturing the soluble form or also protein from cell membranes (e.g., arising from in vivo sources such as exosomes or from sample handling). Complementary cytometry data on paired samples would provide additional insights.

In summary, this study provides the most comprehensive map of the plasma proteome in SLE to date. Stratification by autoantibody status reveals distinct proteomic endotypes even after adjustment for disease activity. Our data provide insights into pathogenesis and highlight potential therapeutic targets.

## Methods

### Sex as a biological variable.

Samples analyzed in this study were obtained from male and female patients and healthy controls. The majority of patients were female (Table 1), reflecting the sex bias in the prevalence of SLE. Sex was included as a covariate in all statistical models.

### Patients and samples.

We recruited 268 patients with SLE between 2019 and 2023 from the Imperial Lupus Centre at the Hammersmith Hospital, London, UK. Inclusion criteria were (i) fulfilled the 1997 American College of Rheumatology criteria, (ii) positive for ANA, (iii) aged 18 or over, and (iv) able to provide informed consent. We recruited 86 HVs to provide a control group, sampled as 2 temporally distinct groups comprising 45 and 41 individuals, respectively. No patients had received anifrolumab since this drug is not licensed in the UK. Peripheral blood was collected in EDTA tubes and centrifuged at 1,000*g* for 10 minutes at room temperature within 3 hours of venipuncture and stored at –70°C without freeze-thaw before assay processing.

### Proteomic measurements and quality assessment.

Plasma proteins were measured using the aptamer-based SomaScan v4.1 platform. The SomaScan v4.1 platform uses modified aptamers (SOMAmers) that bind to their targeting proteins for measurement. A total of 7,288 SOMAmers targeting 6,595 unique human proteins were measured (some proteins were assayed by multiple distinct SOMAmers; Supplemental Data File 2). To avoid interference of anti-dsDNA autoantibodies with the SOMAmers, a validated anti-DNA antibody protocol was used (10). This involved adding herring sperm DNA to saturate anti-dsDNA autoantibodies within each sample and prevent these antibodies from binding the SOMAmers. The platform contains buffer replicates containing no protein that are used to estimate the background noise level, calibrator replicates to control for intraplate variations and batch effects across experiments, and quality control (QC) replicates for signal normalization and QC check. Multiple normalization and calibration steps were performed using SomaLogic’s standard protocol. Data normalized by adaptive normalization by maximum likelihood was used for our analyses. Relative fluorescence units were log_2_ transformed before analyses.

Proteomic measurements were performed in 2 separate batches 2 years apart: batch A consisted of 226 SLE and 45 healthy samples and batch B of 57 SLE and 41 HV samples. The reason for performing proteomic measurements in 2 batches was due to funding availability. Assignment of SLE to batches was by random sampling, but for HVs samples were obtained in 2 distinct periods. No statistically significant differences in demographics or clinical characteristics of the SLE patients (e.g., age, sex, SLEDAI-2K, disease duration) were observed between batches (Supplemental Table 1).

In addition to the manufacturer’s quality assessment, we performed additional steps including PCA and inspection of sample distributions via box-and-whisker plots. We identified batch effects, with separation of batch A and batch B on PCA (Supplemental Figure 26, A and B). After manual examination on the PCA plot, 3 samples from batch A were removed since they were distinctly distributed from the main cohort of samples (>3 standard deviations from the means of PC1 and PC2) and also processed on the same day, suggesting technical rather than biological variation (Supplemental Figure 26A). Additionally, 1 sample from batch A and 4 samples from batch B were removed since they were flagged as potential poor quality samples by SomaScan’s standard QC pipeline requiring extremely high (>2.5) or low (<0.4) scaling factors for normalization. For 6 patients, serial longitudinal samples were available. In these instances, we retained the first sample for each patient and excluded the others from all downstream analyses except for the evaluation of intra-individual changes over time in disease activity–associated markers (see below). For the main analyses (excluding serial samples), post-QC sample sizes were as follows: batch A, 207 SLE and 45 HVs; batch B, 53 SLE and 41 HVs.

### Missing values.

No missing values were observed in the SomaScan proteomic datasets across either proteins or samples. For the clinical metadata, a small number of samples had missing data on the status of autoantibodies and treatments (enumerated in Table 1) and these samples were omitted from analyses requiring these metadata. Sample sizes used in each are reported in the relevant figures and supplemental tables.

### Protein nomenclature.

Due to non-standardization of protein nomenclature (e.g., where a given protein has multiple names) and the lack of human interpretability of UniProt identifiers, we annotated each protein using the non-italicized HUGO symbol for the encoding gene. In instances where there were multiple distinct SOMAmers mapping to the same HUGO gene symbol (1,669 SOMAmers mapped to 781 unique gene symbols and 782 UniProt identifiers), we concatenated the gene symbol with the SOMAmer identifier to preserve a unique identifier for each analyte. Importantly, in some instances, multiple SOMAmers mapping to the same gene symbol represented measurements of distinct biological entities. For example, the intact complement C3 protein and its distinct cleavage products such as C3a, C3b, and C3d, all mapped to a single HUGO gene symbol and UniProt ID. However, in other instances there were multiple SOMAmers that are nominally directed against the same target protein. A comprehensive annotation file mapping of SOMAmers to their target proteins, UniProt IDs and corresponding gene IDs, and gene symbols is provided in Supplemental Data File 2.

### Complement proteins.

Various complement proteins and cleavage fragments were included on the SomaScan platform. To evaluate the accuracy of complement C3 and C4 quantification, we compared the levels of intact complement C3 and C4 proteins measured by SomaScan in SLE patients to their corresponding values measured in the clinical laboratory from the same blood draw. We performed this analysis separately for each batch to avoid variation related to batch.

### Identification of differentially abundant proteins in SLE versus HV samples.

For each protein measured, multiple linear regression was used to analyze differences in protein abundance between SLE and HV samples using the lm() function in R. Age and sex were adjusted for by including them as covariates. The regression model in Wilkinson notation was *Prot* ~ *D* + age + sex, where *Prot* is plasma level for a given protein and *D* is a binary variable for disease status (SLE vs. HV).

In batch B, we observed inflation of test statistics and an unusually high proportion of proteins that were significantly differentially abundant in the comparison of SLE versus HVs (Supplemental Figures 27–29). We also observed marked separation of SLE and HV samples on PC1 (when PCA was performed limiting to batch B; Supplemental Figure 30), with PC1 accounting for 42% of the variance. The most upregulated protein was PGAM1, which has been identified by SomaLogic as a marker of preanalytical variation including time to spin, suggesting that systematic non-biological variation was confounding the differences between SLE and HVs in batch B (Supplemental Figures 27 and 31). We therefore included the first principal component as a covariate to adjust for preanalytical variation in batch B (see Supplemental Material).

*P* values were adjusted using the Benjamini-Hochberg (BH) method (*P*_BH_) to control the FDR across multiple tests (7,288 protein analytes). To enhance the robustness of SLE-associated proteins reported here, we required a protein to be significant (FDR < 0.05) in SLE versus HV comparisons in both batch A and in batch B.

We then meta-analyzed the results of the batch A and batch B analyses using the R package metafor (v4.8-0) (64) with inverse variance–weighted fixed-effect models to obtained combined effect size estimates across the 2 batches.

### Identification of proteins associated with disease activity.

Disease activity was assessed using SLEDAI-2K (65) at the same time as the research blood sample. Given that SLEDAI-2K scores have a highly non-normal distribution and can be sensitive to the weightings attributed to specific clinical features, prior to testing for association with proteins, we categorized disease activity into a 4-level ordinal variable as follows: HDA was defined as SLEDAI-2K ≥ 10; MDA as SLEDAI-2K between 5 and 9; LDA as SLEDAI-2K ≤ 4 but not meeting the definition for remission; and “remission” as defined by the Definition of Remission in SLE (DORIS) criteria (66), characterized by clinical components of SLEDAI-2K = 0 (i.e., not including anti-dsDNA antibody or complement status) and a prednisolone dose of ≤5 mg/day.

To identify proteins associated with disease activity, we performed multiple linear regression for each protein using the lm() function in R. Protein abundance was regressed on disease activity (coded as an ordinal variable: “In remission,” “LDA,” “MDA,” “HDA”), with sex and batch as covariates.

We evaluated effectiveness of batch adjustment through PCA plots of residuals after regressing protein levels on batch. We also compared our primary analysis modeling batch as a covariate with batch correction using ComBat (15) (Supplemental Material and Supplemental Figures 6 and 32).

As a sensitivity analysis to explore the effect of the method of encoding disease activity, Spearman’s rank correlation was conducted between protein abundance and SLEDAI-2K scores. Prior to correlation testing, protein abundances were adjusted to account for sex and batch effects using a linear model and the resulting residuals were used in the Spearman’s correlation test versus SLEDAI-2K scores.

### Identification of proteins associated with autoantibody status.

We analyzed proteomic associations with the presence of antibodies against Sm, dsDNA, and phospholipids. For each plasma protein, abundance (*Prot*) was regressed on antibody status (*Ab*; a binary variable) with batch and sex included as covariates. The regression model in Wilkinson notation was *Prot* ~ *Ab* + batch + sex. To identify associations with anti-Sm antibodies independent of disease activity, we then repeated the analysis, adding disease activity status as a covariate to the model.

For the anti-dsDNA analysis, antibodies were measured in the clinical laboratory from the same blood draw as the research sample. Samples were classified as anti-dsDNA negative if the level was lower than 10 IU/mL (the manufacturer’s defined threshold) and as anti-dsDNA positive if the patient’s plasma anti-dsDNA autoantibodies level was higher than 20 IU/mL. Patients with anti-dsDNA levels (*n* = 40) within the borderline range of 10–20 IU/mL were excluded in this analysis. To identify proteins associated with anti-dsDNA positivity independent of disease activity, we used a modified SLEDAI-2K score excluding the anti-dsDNA component from the score to avoid overadjustment, since SLEDAI-2K includes 2 points for dsDNA antibody positivity. We recalibrated the disease activity category thresholds between LDA, MDA, and HDA based on this modified score (DORIS remission definition was unchanged since this does not include anti-dsDNA status). Specifically, we normalized the modified SLEDAI-2K by the highest possible score excluding dsDNA antibody positivity (i.e., 105 – 2 = 103), and applied the same procedure to the unmodified SLEDAI-2K thresholds used to define activity categories described earlier. To illustrate this procedure, an individual positive for anti-dsDNA antibodies with an unmodified SLEDAI-2K score of 11 would have been defined as being in HDA (defined as SLEDAI-2K of 10 or higher) in the earlier analyses. The modified SLEDAI-2K score would be 9. The normalized score would be 9/103 = 0.087. The new cutoff for HDA would be 10/103 = 0.097. Thus, this individual would be classified as in MDA for the purposes of covariate adjustment in the analysis testing for proteins associated with anti-dsDNA antibodies independent of disease activity.

As a sensitivity analysis, we tested protein abundances against anti-dsDNA titer (as a continuous variable) using all available SLE samples. To do this, protein abundances were first adjusted to account for sex, batch, and disease activity (as an ordinal variable) using a linear model and the resulting residuals were used in the Spearman’s correlation test versus anti-dsDNA titer.

To formally test whether CD40LG’s association with disease activity was dependent on anti-dsDNA antibody status, we fitted a regression model with an anti-dsDNA antibody status (*Ab*) × disease activity interaction term: CD40LG ~ *Ab* + disease_activity + *Ab*:disease_activity + batch + sex. Here, *Ab* was a binary variable indicating negative (“0”) or positive (“1”) anti-dsDNA antibody status.

### Pathway enrichment analyses.

For SLE-versus-HV comparisons and disease activity analysis, we performed GSEA using the fgsea R package (version 1.24.0) (67) and the Human MSigDB Collection “C2: curated gene sets” (sub-collection: “Canonical pathways”) which consists of 4,023 gene sets (v2025.1) mainly sourced and curated from BioCarta, Kyoto Encyclopedia of Genes and Genomes (KEGG), Pathway Interaction Database, Reactome, and WikiPathways (68). For SLE-versus-HV comparisons, GSEA was performed separately in each batch. We then used robust-rank aggregation (RRA) (69) to identify the pathways most consistently enriched across both batches. RRA outputs a score analogous to a *P* value for each pathway term. These scores were –log_10_ transformed to represent the aggregated score. A higher aggregated score indicates stronger consistency.

Over-representation tests were used to identify enriched pathways in WGCNA protein modules and antibody-associated protein lists, implemented using the enrichPathway function from the ReactomePA R package (v1.42.0) (70). Proteins were first mapped to corresponding Entrez IDs since this package requires Entrez ID annotation. Entrez IDs mapped to proteins in the module were tested against the background set of 6,363 Entrez IDs representing the proteins measured on SomaScan v4.1. All pathway enrichment analyses were restricted to protein sets containing 10–500 proteins to exclude very small sets vulnerable to noise and large sets representing broad and non-specific processes.

### Protein network analysis.

Network analysis was performed using WGCNA (71) as detailed in the Supplemental Methods. Sensitivity analyses were performed by modifying the parameters used in WGCNA to evaluate the stability of identified modules (Supplemental Figures 33 and 34).

### Annotation of ISPs.

To identify ISPs, we obtained a list of ISGs curated from the Interferome database (72) as detailed in the Supplemental Methods.

### Whole-blood ISG score.

The whole-blood ISG score was measured as described previously (73). Details are described in the Supplemental Methods.

### Statistics.

Details of regression models are provided in the relevant sections above. Multiple testing correction was performed using the Benjamini-Hochberg procedure. We defined statistical significance as a *P*_BH_ of less than 0.05 (i.e., leading to FDR < 0.05). The criterion for classification of a protein as SLE-associated in the SLE versus HV analysis is described above. All statistical tests were 2-sided. For box-and-whisker plots, the lower and upper bounds of the box indicate the first and third quartiles, respectively. The horizontal line within the box represents the median. Whiskers indicate the most extreme data point that is no more than 1.5 times the IQR from the box. All individual points are shown.

### Study approval.

Human samples used in this research project were obtained from the Imperial College Healthcare Tissue and Biobank (ICHTB). ICHTB is supported by the National Institute for Health Research Biomedical Research Centre based at Imperial College Healthcare NHS Trust and Imperial College London. ICHTB is approved by Wales REC3 to release human material for research (22/WA/0214). Ethical approval was provided by the ICHTB (Human Tissue Authority Licensing number 12275; Project number R14042-3A; sub-collection IMM_MB_13_001) and informed consent obtained from all participants.

### Resource availability.

Requests for further information and resources should be directed to the corresponding author, James E. Peters (j.peters@imperial.ac.uk).

### Materials availability.

This study did not generate new unique reagents.

### Data availability.

Individual-level proteomic data and meta-data have been deposited to Zenodo (https://doi.org/10.5281/zenodo.17692315). All data associated with the results reported in this study are available in the Supporting Data Values file.

## Author contributions

GHDL: analysis, methodology, visualization, manuscript writing. CB and NB: data acquisition, project administration. RTM: data acquisition, project administration, manuscript editing. BJW: visualization, manuscript editing. BZ: analysis. YJH: data acquisition. TTS, MC, LL, and TC: data acquisition. MB, MCP, and JEP: designed the study, funding acquisition, methodology, supervision. JEP: manuscript writing with editing from MB and MCP.

## Conflict of interest

LL is an Advisor/Consultant to Alexion, Argenx, Astra Zeneca, Boehringer Ingelheim, Carna Health, GSK, Incyte, Kezar, Nkarta, Novartis, Otsuka, Pfizer, Roche, Sanofi, Vera, and Vertex; on the speaker bureaus of Astra Zeneca, GSK, Otsuka, and Roche; and on trial data monitoring committees for Nkarta and Novartis.

## Funding support

- Medical Research Foundation Fellowship MRF-057-0003-RG-PETE-C0799 (to JEP).
- NIHR Imperial Biomedical Research Centre Immunology Theme.
- Imperial College Healthcare Tissue and Biobank (infrastructure funded by the NIHR Imperial Biomedical Research Centre).
- Wellcome Trust Senior Fellow in Clinical Science grant 212252/Z/18/Z (to MCP).

## Supplementary Material

Supplemental data

Supplemental data sets 1-15

Supporting data values

## Figures and Tables

**Table 1:**
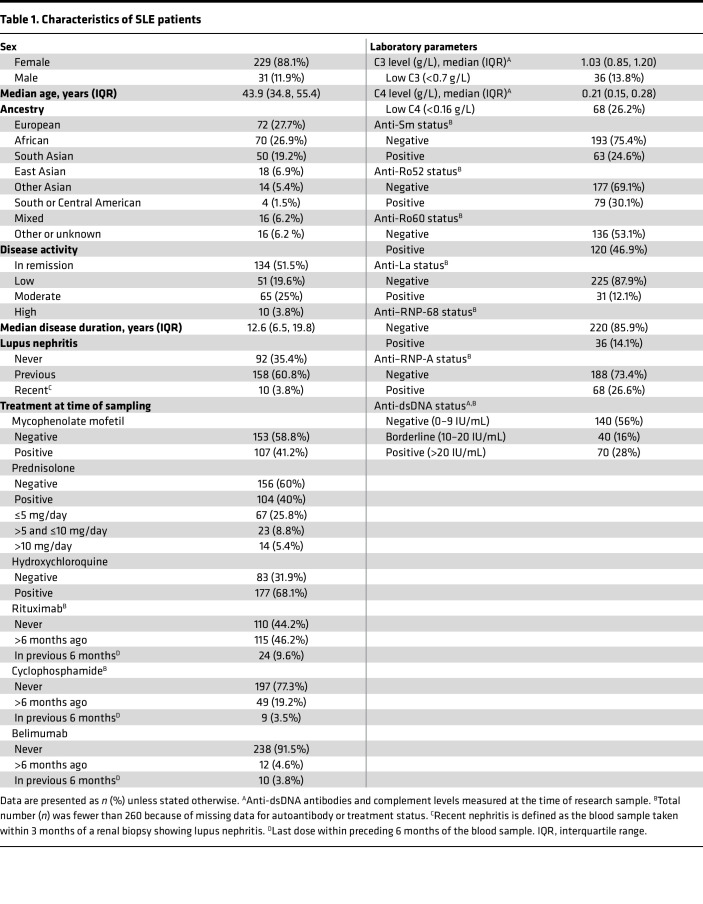
Characteristics of SLE patients

## Version 1 06/02/2026

In-Press Preview

## Version 2 07/22/2026

Electronic publication
