# Supplementary material for: Mapping the plasma proteomic architecture of systemic lupus erythematosus
Source: JCI Insight. 2026 Jun 2;11(14):e206938. doi: 10.1172/jci.insight.206938 (PMC13461151; doi:10.1172/jci.insight.206938)
Supplement: Supplementary file 1 [file jciinsight-11-206938-s384.pdf]

Supplementary material for 'Mapping the plasma proteomic architecture of systemic lupus erythematosus' by Leung et al

Table of Contents

Supplementary Note

1. Correction for pre-analytical variation confounding SLE vs HV differential abundance analysis in Batch B
2. Definition of significance in SLE vs HV differential protein abundance testing
3. Batch correction for within SLE cases analyses
4. Sensitivity analyses for potential confounding variables
5. Sensitivity analysis by adjusting the parameters of WGCNA
6. Exploratory Longitudinal Analysis

Supplementary Methods

1. Protein network analysis
2. Annotation of ISPs
3. Whole-blood interferon stimulated gene score

Supplementary Table

Supplementary Figures

Titles for Supplementary Data

Supplementary Note

1. Correction for pre-analytical variation confounding SLE vs HV differential abundance analysis in Batch B

Principal component analysis performed separately on each batch of data showed that the SLE were sharply demarcated from HV samples in Batch B on plots of PC1 vs PC2. This pattern was not observed in Batch A, where SLE and HV samples were more overlapping on the PCA plot (**Supplementary Figure 30**). Moreover, PC1 explained 42% of the variance in the dataset in Batch B.

Differential protein abundance testing of SLE vs HVs in Batch B resulted in a very high proportion of significantly differentially abundant proteins (DAPs). 5,643 (77%) of 7,288 measured proteins) were significantly differentially abundant at 5% false discovery rate (**Supplementary Figure 27**). This resulted in an unusual appearance of the volcano plot. In contrast, in Batch A, only 1,821 proteins were significant, despite a much larger sample size (207 SLE, 45 HVs) and hence statistical power. We confirmed the impression of inflated signals in Batch B using QQ plots (**Supplementary Figure 28**). This finding could not be explained by differences in disease activity (median SLEDAI = 4 in batch A, median SLEDAI = 2 in batch B; P = 0.07) or other clinical characteristics between the Batches. We performed a number of sensitivity analyses, including restricting to females only, but the inflated signals in Batch B persisted. For SLE samples, we had contemporaneous clinical laboratory measurements of intact complement C4 available and so tested these with SomaScan-measured intact complement C4. This revealed very strong correlations between SomaScan and clinical lab measurements of intact C4 in both Batch A and Batch B, suggesting that the SomaScan measurements were reliable in the SLE samples (**Supplementary Figure 10B**). This led us to suspect that the source of inflation might be the pre-analytical variation related to the HV samples in Batch B. Potential sources of variability for HV samples in Batch B included differences in personnel performing sample processing and time to spin. In contrast, for Batch A HV samples and SLE samples the sample handling was more consistent. Moreover, PGAM1 was most upregulated with the greatest effect estimate ($\log_2$ fold change) in SLE vs HVs in batch B samples. This marker has been identified by SomaLogic as suggesting pre-analytical variation and is affected by time to spin.

Given this and the separation on the PCA plots, we computed the first principal component (PC) for all batch B samples and included it as a covariate in linear regression models for SLE vs HV differential abundance testing in the batch B analysis. Following PC-adjustment, the proportion of

significantly differentially abundant proteins at 5% FDR fell to 5.9% and the QQ plots were improved **(Supplementary Figures 27-28)**. In addition, the strongest signals were interferon-stimulated proteins, i.e. a biological plausible finding.

Comparison of beta estimates and significant proteins (5% FDR) between the two batches are shown in **Supplementary Figure 27**. Without PC1 adjustment in Batch B, 4,182 proteins were significant in Batch B but not Batch A (despite smaller sample size in Batch B), and many proteins with large effect sizes in Batch B had no significant effect in Batch A. Following PC1-adjustment in Batch B, there was greater concordance of effect size estimates.

Exploration of other approaches

As an alternative to PC adjustment, we explored the use of Surrogate Variable Analysis (SVA) to correct for pre-analytical variation. We explored inclusion of between 1 and 10 latent factors from SVA as covariates in the regression model. Analysis adjusted for the first latent factor did not correct inflation (**Supplementary Figure 29**), with 5704/7288 (78%) analytes significantly differentially abundant at 5% FDR, similar to the results when no adjustment was applied, and the artefactual association with the marker of pre-analytical variation PGAM1 was not attenuated (**Supplementary Figure 31C**). Inclusion of more latent factors did not correct pre-analytical variation (**Supplementary Figure 31D**). In summary, adjustment for PC1 outperformed the SVA approach to reduce pre-analytical variations in batch B.

2. Definition of significance in SLE vs HV differential protein abundance testing

Because of the issues with pre-analytical variation in the SLE vs HV analysis outlined above, we decided to adopt a conservative approach to defining a protein as differentially abundant, utilizing the batch structure. To declare a protein as SLE-associated protein, we required it to be independently significant in *both* Batch A and in Batch B (after PC adjustment) at FDR <0.05 (where multiple testing correction was performed across all proteins in each batch, rather than a typical discovery-replication strategy where multiple testing in the replication cohort is limited to significant hits in the discovery cohort).

3. Batch correction for within SLE cases analyses

For within cases analyses (e.g. testing for association with disease activity and autoantibody status) we analysed data from Batch A and Batch B together to maximise power, adjusting for batch as a covariate in our regression models. To evaluate the success of including batch as a covariate, we performed PCA analysis on residuals generated after regressing protein levels on

batch. Comparison of PCA plots on the unadjusted proteomic data with those on the residuals after regressing out batch revealed successful removal of the batch effects (**Supplementary Figures 26B and 32A**).

We also explored an alternative batch correction method, ComBat (1). PCA plots after application of ComBat showed successful removal of batch effects **(Supplementary Figure 32B)**.

We compared the results of our primary analysis for SLE-activity (i.e. including batch as a covariate) with those from ComBat-adjusted data. These showed very similar results: a) effect size estimates were very highly correlated (r 0.99 for comparison of all proteins), b) of the 421 activity-related proteins from our primary analysis, 419 were also significant when analysed ComBat data (**Supplementary Figures 6A-B**).

Finally, we performed a comparison to the results restricting to Batch A samples alone; again similar results (**Supplementary Figures 6C-D).**

In conclusion, these results show our approach for adjusting for batch effects in the within cases analyses were robust.

4. Sensitivity analyses for potential confounding variables

Our dataset consisted of patients with varied disease durations (from diagnosis to time of sampling) and treatment(s) at the time of sampling. To assess whether these might have affected the results of our analyses, we performed multiple sensitivity analyses and compared the effect sizes of each protein with those obtained from the primary analyses. These included:

1. Limiting samples to those with disease duration ≤ 5 years (**Supplementary Figures 5A, 8B, 18B, 19C**);
2. Adjusting for disease duration as a covariate in the regression model (**Supplementary Figures 8A, 18A, 19B**);
3. Excluding samples that were on MMF at time of sampling (**Supplementary Figure 5B**);
4. Excluding samples that were on Prednisolone >5mg/day at time of sampling (**Supplementary Figure 5C**);
5. Excluding samples that had recently (within preceding 6 months) received Rituximab, Cyclophosphamide, or Belimumab, and adjusting for MMF use and Prednisolone dose (**Supplementary Figures 5D, 8C, 18C, 19D**).

These sensitivity analyses showed that the estimated effect sizes for our major analyses remained largely consistent, acknowledging that subsetting samples leads to loss of power and less precision in effect estimates.

5. Sensitivity analysis by adjusting the parameters of WGCNA

In our primary WGCNA analysis, we selected a soft-thresholding power of 6 since this was the lowest power to achieve a scale-free topology ($R^2 > 0.9$; **Supplementary Figures 33 and 34**). To examine whether the protein modules identified in our study were affected by parameter choice, we re-ran WGCNA using powers of 5 and 7 as sensitivity analyses. These showed that "interferon-associated" (correlating with whole-blood ISG score measured by qPCR) and "renal-associated" modules (correlating with serum Creatinine and urinary protein:creatinine ratio) were identified across all tested powers (5,6, and 7) (**Supplementary Figure 33**). Pathway enrichment analyses also revealed the same major pathways represented by these modules as in our primary analysis (**Supplementary Figure 34**).

In addition to varying the soft-thresholding power, we also assessed whether minimum module size had any effect on our WGCNA results by increasing 20 to 30. Again, this showed no material changes to the modules' allocation (**Supplementary Figures 33 and 34**).

6. Exploratory Longitudinal Analysis

For 3 patients, longitudinal samples taken at 4 or more timepoints were available. The limited sample size precluded formal biomarker analysis, but we used these data to qualitatively explore selected proteins that we had identified as disease activity-associated in the cross-sectional analysis. We first selected proteins that were: (i) potential drug targets, defined as having an associated compound with a characterized mechanism of action in Open Targets (Dataset: "Drug - mechanism of action"; data version 25.03), and (ii) were members of the pathways significantly enriched among disease activity–associated proteins. From this list we then selected the 5 proteins with the greatest effect sizes in the ordinal linear regression analysis testing for association with disease activity. These criteria resulted in 5 proteins, comprising CXCL10, CCL2, CD40LG, HAVCR2, and C3d (measured by SOMAmer seq.5803.24), one of which was an interferon-stimulated protein (CXCL10). In addition, we added two interferon-stimulated proteins (MX1 and B2M) that were significantly associated with disease activity.

We visualised the longitudinal relationships between these 7 proteins and SLEDAI scores and disease activity-related serological markers (clinically measured C3, C4, and anti-dsDNA

autoantibody levels). Our data showed that the levels of these proteins generally tracked with changes in disease activity over time **(Supplementary Figure 9A)**. For example, in Patient 1, who exhibited a progressive decline in disease activity leading to remission, most of the selected proteins showed a consistent decrease in abundance across timepoints. Repeated measures correlation was used to assess the association between these proteins and clinically measured disease activity traits, accounting for the non-independence of observations due to repeated measurements within the same individuals (2). This approach is more appropriate than Pearson's correlation, which assumes independent observations. This confirmed strong within-patient relationships: all proteins were significantly correlated with at least 1 clinical parameter, showing positive correlations with SLEDAI-2K and anti-dsDNA titres and negative correlations with C3 and C4 levels **(Supplementary Figure 9B)**. For example, CD40LG was strongly correlated with anti-dsDNA levels (r 0.95, P_{BH} 5.6×10^{-4}; **Supplementary Figure 9C**) consistent with the key role of the CD40LG-CD40 pathway in B cell proliferation/differentiation and antibody production (3). Longitudinal changes in C3d were inversely correlated with clinically measured C3 (**Supplementary Figure 9D**), in keeping with the known biology of the complement pathway activation whereby cleavage of C3 reduces the amount of intact C3 and increases C3d. Notably, several proteins returned to normal levels (within the IQR of HV samples) as disease activity diminished. However, the levels of the ISPs CXCL10, MX1 and B2M failed to fully normalise, suggesting persistent interferon pathway activation.

Supplementary Methods

1. Protein network analysis

We applied the Weighted Gene Co-expression Network Analysis (WGCNA) package (v1.72-5) (4) to identify modules of correlated proteins. For this analysis we used SLE samples (n=207) from Batch A. An unsigned network was constructed, and a soft thresholding power of 6 was selected based on the standard criteria of approximate scale-free topology and preservation of mean network connectivity. Networks were constructed and modules were identified by the adjacency and topology overlap matrices, with minimum module size set to 20. We then refined the modules by their similarity based on modular eigenprotein (representing the first principal component of each module's protein abundance profile) correlation using a dynamic tree-cut approach. Modules were merged if their eigenprotein-based dissimilarity was below a threshold of 0.25. The resulting modular eigenproteins were then used to correlate with 20 clinical traits using Spearman's rank correlation test, including 11 continuous traits: age, SLEDAI-2K, neutrophil count, lymphocyte count, haemoglobin, creatinine, urine protein-creatinine ratio (uPCR), albumin, complement C3, complement C4, and whole-blood ISG score (see below), and 9 binary traits: positivity of antibodies against cardiolipin (ACA), RNP-A, RNP-68, Sm, La, Ro52, Ro60, dsDNA, and sex. Correlations with $P_{BH} < 0.05$ were considered significant after correcting for 21 modules x 20 traits = 420 tests. Eigenprotein-based connectivity, or module membership, was calculated for each protein in each module by correlation between the protein abundance levels and the corresponding module's eigenprotein values. The protein with highest module membership was then defined as the hub protein for each module. The network of the proteins in the selected module was constructed using the topology overlap matrix which calculates the strength of similarity between each pair of proteins. The network was then visualised in Cytoscape (v3.10.3) (5), with nodes representing proteins and thickness of edges representing the strength of similarity. Only the edges with a threshold value of > 0.03 were included in the visualised network. Sensitivity analyses were performed by modifying the parameters used in WGCNA to evaluate the stability of identified modules (**Supplementary Figures 33 and 34**).

2. Annotation of ISPs

To identify ISPs, we obtained a list of ISGs curated from the Interferome database (v2.01) (6), which collected gene expression data derived from multiple in vitro and in vivo microarray experiments of IFN stimulation. Searches were restricted to type I interferons and human datasets, a fold-change threshold of ≥2.0 for upregulated or downregulated genes, and IFN treatment time between 0 and 12 hours. All other search parameters were left at their default settings.

3. Whole-blood interferon stimulated gene score

The whole-blood interferon-stimulated gene score was measured as described previously (7). In brief, total RNA was isolated from whole blood samples using Tempus RNA isolation kits (Thermo Fisher Scientific) following the manufacturer's protocol. Gene expression was measured by real-time qPCR and relative gene expression of *CMPK2*, *EPSTI1* and *HERC5* were calculated by subtracting the housekeeping gene *HPRT* using the ΔCt method. The ISG score was calculated using the mean ΔCt from *CMPK2*, *EPSTI1* and *HERC5* genes *(-1).

Supplementary Table

	SLE (n=260)			Healthy volunteers (n=86)		
	**Batch A**	**Batch B**	**P^**	**Batch A**	**Batch B**	**P^**
**Samples, N [%]**	207	53		45	41	
Female	184 [88.9%]	45 [84.9%]	0.57	38 [84.4%]	20 [48.8%]	<0.001
Male	23 [11.1%]	8 [15.1%]		7 [15.6%]	21 [51.2%]	
**Median age, Years [IQR]**	44 [35.2, 55.2]	42.3 [34.4, 56.3]	0.86	34.2 [27.8, 40.4]	47.0 [38.1, 55.2]	<0.001
**SLEDAI-2K [IQR]**	4 [0, 6]	2 [0, 4]	0.065	-	-	
**Disease activity, N [%]**				-	-	
In remission	100 [48.3%]	34 [64.2%]		-	-	
Low	43 [20.8%]	8 [15.1%]	0.12	-	-	
Moderate	54 [26.1%]	11 [20.8%]		-	-	
High	10 [4.8%]	0 [0.0%]		-	-	
**Median disease duration, Years [IQR]**	12.6 [6.5, 19.7]	12.6 [6.6, 19.9]	0.86	-	-	
**Lupus nephritis, N [%]**				-	-	
Never	76 [36.7%]	16 [30.2%]		-	-	
Previous	123 [59.4%]	35 [66.0%]	0.67	-	-	
Recent*	8 [3.9%]	2 [3.8%]		-	-	
**Laboratory parameters**						
C3 level (g/L, Median [IQR])†	1.02 [0.85, 1.23]	1.04 [0.85, 1.15]	0.53	-	-	
Low C3 (<0.7g/L), N [%]	31 [15.0%]	5 [9.4%]		-	-	
C4 level (g/L, Median [IQR])†	0.21 [0.14, 0.28]	0.22 [0.18, 0.29]	0.65	-	-	
Low C4 (<0.16g/L), N [%]	57 [27.5%]	11 [20.8%]		-	-	
Anti-Sm status, N [%]‡				-	-	
Negative	153 [75.0%]	40 [76.9%]	0.91	-	-	
Positive	51 [25.0%]	12 [23.1%]		-	-	
Anti-dsDNA status, N [%]†‡				-	-	
Negative (0-9)	112 [56.3%]	28 [54.9%]		-	-	
Borderline (10-20)	33 [16.6%]	7 [13.7%]	0.79	-	-	
Positive (>20)	54 [27.1%]	16 [31.4%]		-	-	
**Treatment at time of sampling, N [%]**						
Mycophenolate mofetil				-	-	
Negative	122 [58.9%]	31 [58.5%]	1.0	-	-	
Positive	85 [41.1%]	22 [41.5%]		-	-	
Prednisolone				-	-	
Negative	118 [57.9%]	38 [71.7%]	0.073	-	-	
Positive	89 [43.0%]	15 [7.2%]		-	-	
≤5mg/day	54 [26.1%]	13 [24.5%]		-	-	
>5mg, ≤10mg/day	23 [11.1%]	0 [0.0%]	0.073	-	-	
>10mg/day	12 [5.8%]	2 [3.8%]		-	-	
Hydroxychloroquine						
Negative	67 [32.4%]	16 [30.2%]	0.89	-	-	
Positive	140 [67.6%]	37 [69.8%]		-	-	
Rituximab‡				-	-	
Never	89 [45.2%]	21 [40.4%]		-	-	
> 6 months ago	90 [45.7%]	25 [48.1%]	0.77	-	-	
In previous 6 months#	18 [9.1%]	6 [11.5%]		-	-	
Cyclophosphamide‡				-	-	
Never	157 [77.3%]	40 [76.9%]		-	-	
> 6 months ago	39 [19.2%]	10 [19.2%]	0.99	-	-	
In previous 6 months#	7 [3.4%]	2 [3.8%]		-	-	
Belimumab				-	-	
Never	186 [89.9%]	52 [98.1%]		-	-	
> 6 months ago	11 [5.3%]	1 [1.9%]	0.14	-	-	
In previous 6 months#	10 [4.8%]	0 [0.0%]		-	-	

Supplementary Table 1. Characteristics of the study participants stratified by batch. ^P-values for differences between Batch A and Batch B samples. For continuous variables P-values were calculated using Student's t-test and for discrete variables calculated using chi-squared test. *Recent nephritis is defined as blood sample taken within 3 months of a renal biopsy showing lupus nephritis. †Anti-dsDNA antibodies and complement levels measured at the time of research sample. Anti-dsDNA assay units in IU/ml. Normal range 0-9. ‡Differences in N reflect individuals with missing data for autoantibody or treatment status. #Last dose within preceding 6 months of the blood sample.

Supplementary Figures

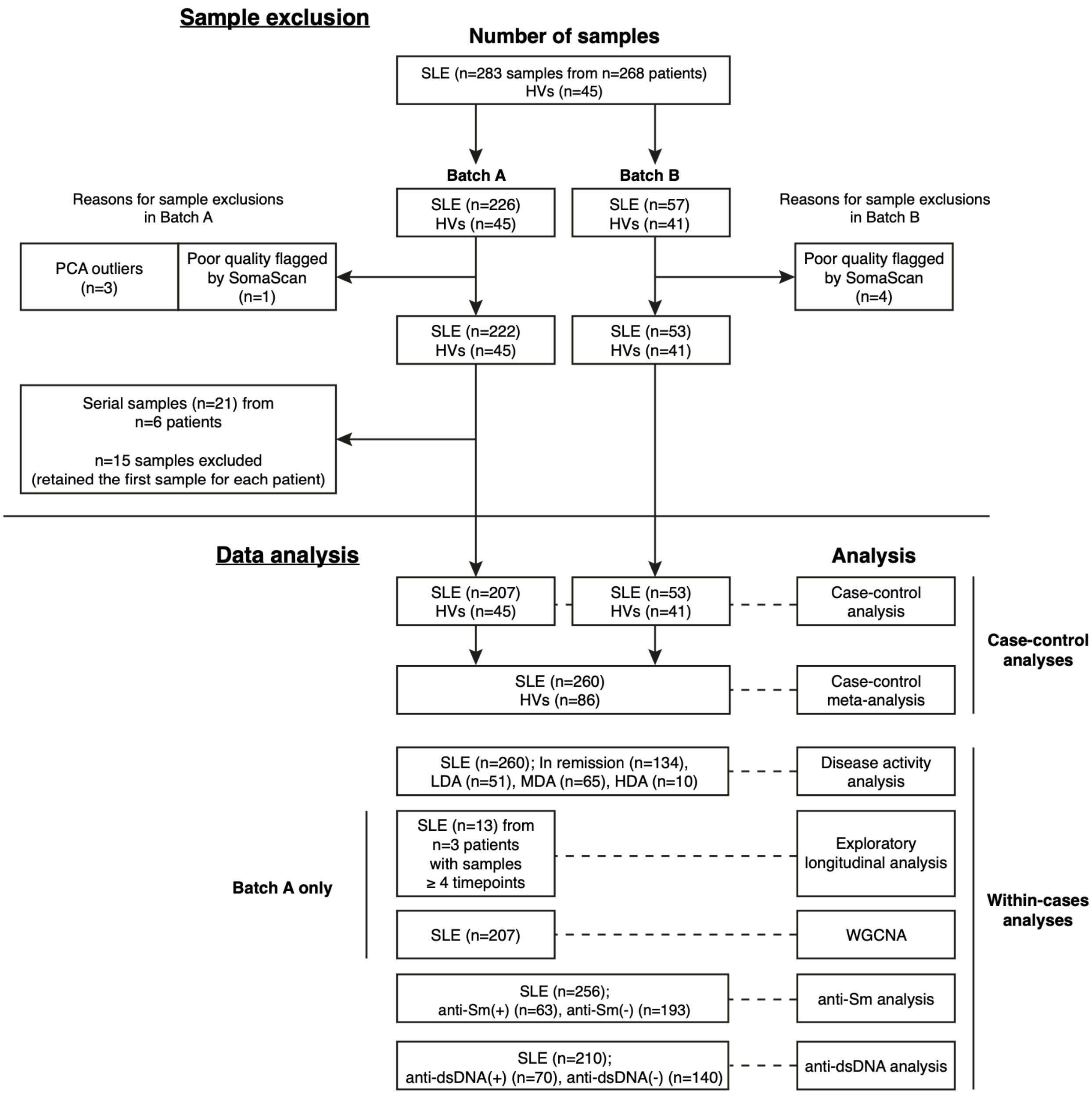

Supplementary Figure 1. Summary of sample exclusions during quality control and final sample sizes for each primary analysis. PCA, Principal component analysis; HVs, Healthy volunteers; WGCNA, Weighted gene co-expression network analysis.

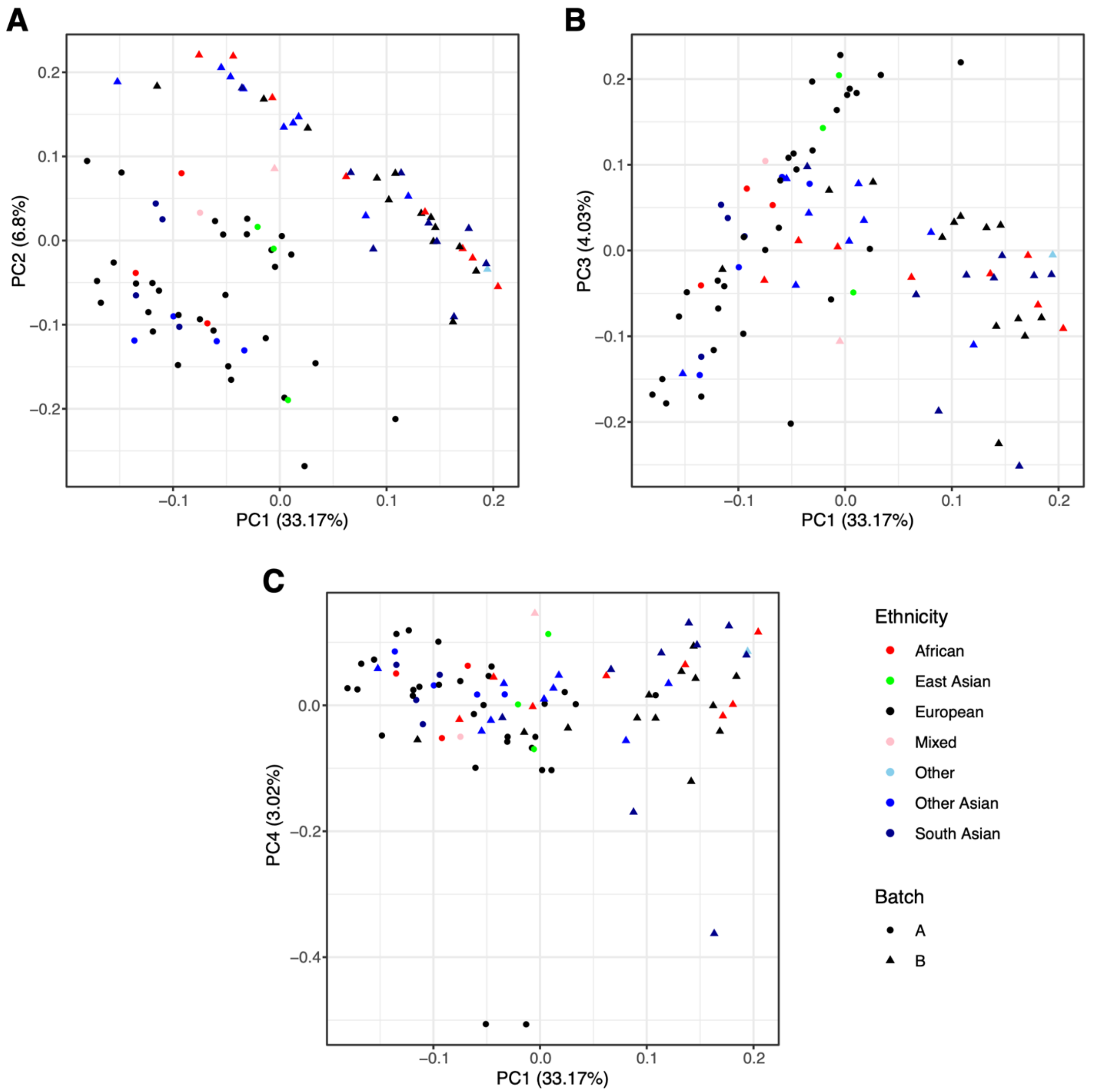

Supplementary Figure 2. Principal components analysis of healthy volunteer proteome stratified by ancestry. A) Principal component (PC) 1 versus PC2 in healthy volunteer samples. **B)** PC1 versus PC3. **C.** PCA of PC1 versus PC4. Colours represent self-reported ethnicity. Circles = Batch A samples; Triangles = Batch B samples.

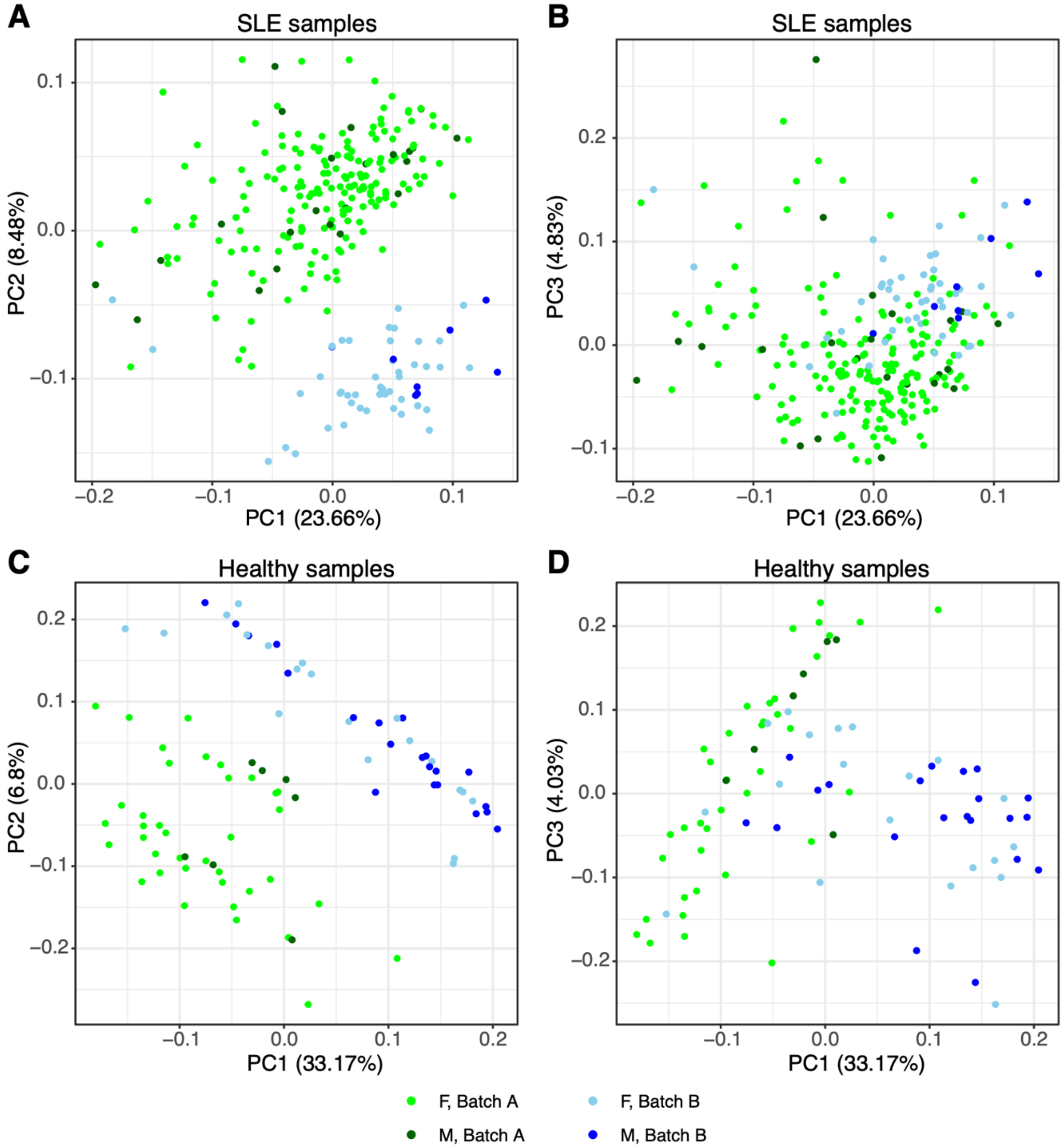

Supplementary Figure 3. Principal components analysis of proteome stratified by sex. A-B) SLE samples. **C-D)** Healthy volunteer samples. Light green = female samples in Batch A; dark green = male samples in Batch A; light blue = female samples in Batch B; blue = male samples in Batch B. PC = prinicipal component.

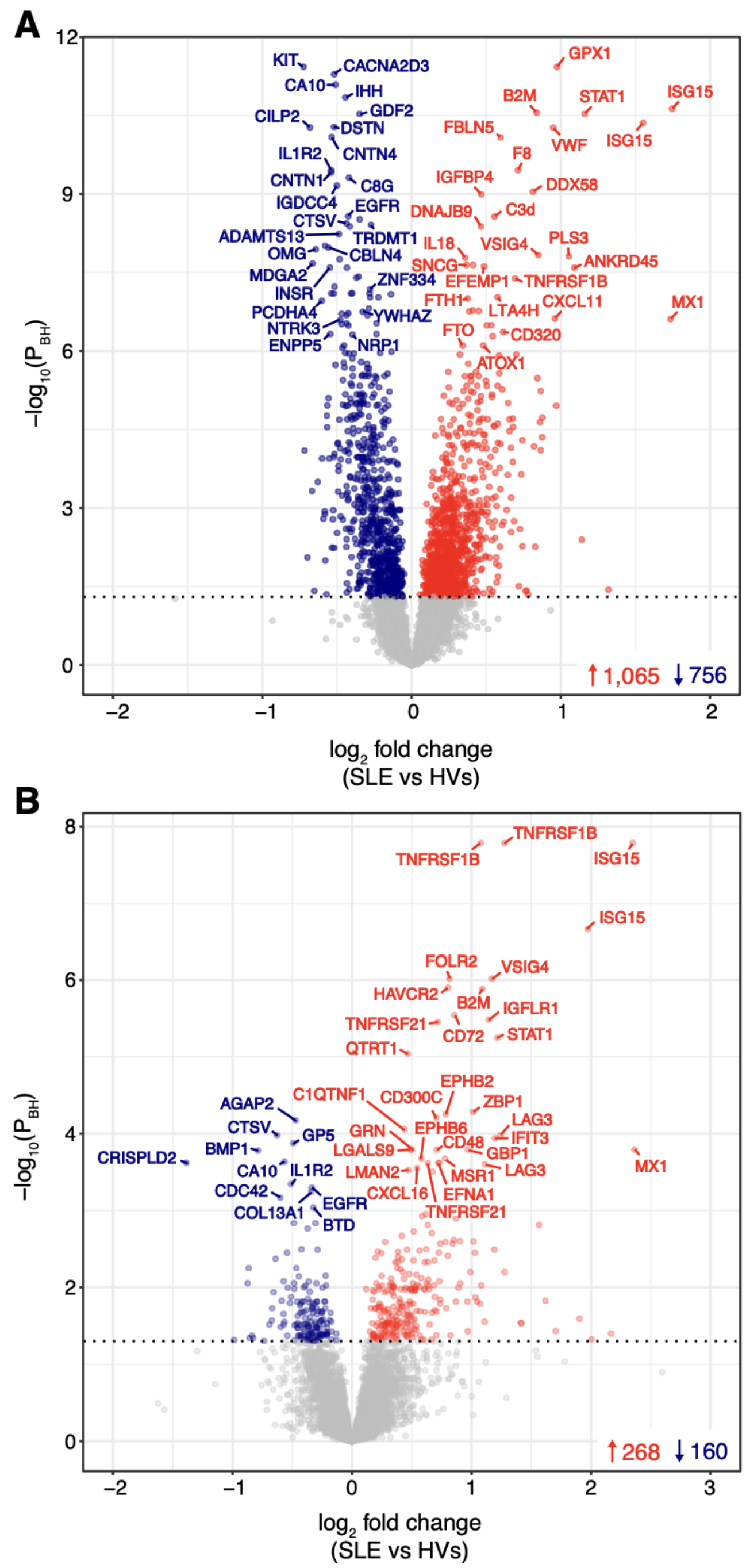

Supplementary Figure 4. Differential abundance analysis of SLE vs HVs. A) Volcano plot showing the $\log_2$ fold change and BH-adjusted P values of each protein from linear regression analyses in batch A. Age and sex were included as covariates. Each point represents a protein. Significantly upregulated proteins are coloured in red while downregulated proteins are coloured in blue. Proteins with BH-adjusted P ≥ 0.05 are colored in grey. **B)** Volcano plot showing the same information as in A, but the analysis was performed in batch B. P_{BH}, Benjamini-Hochberg-corrected P value.

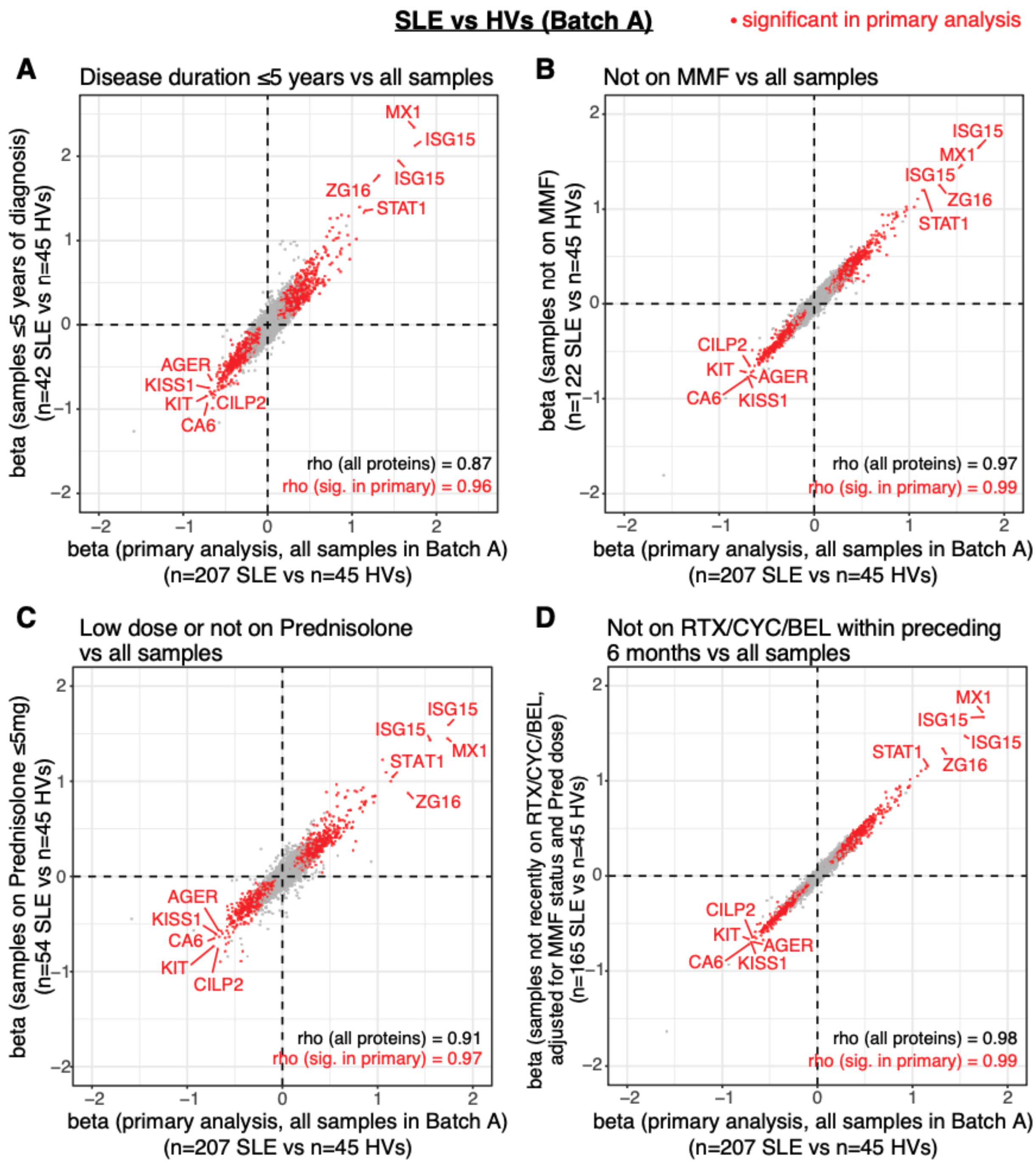

Supplementary Figure 5. Sensitivity analyses for SLE vs HVs differential protein abundance. Comparison of the primary analysis of SLE-vs-HVs using all available samples versus **(A)** restricting to samples taken within 5 years of SLE diagnosis, **(B)** restricting to samples from patients not on MMF, **(C)** restricting to samples from patients on either no or low dose (≤5mg) Prednisolone, and **(D)** excluding samples from patients who received Rituximab, Cyclophosphamide, or Belimumab in the preceding 6 months, and statistically adjusted for MMF treatment status and Prednisolone dose. All results displayed were derived from analysing Batch A ("discovery cohort") samples. Red and grey, significant and not significant in primary analysis, respectively.

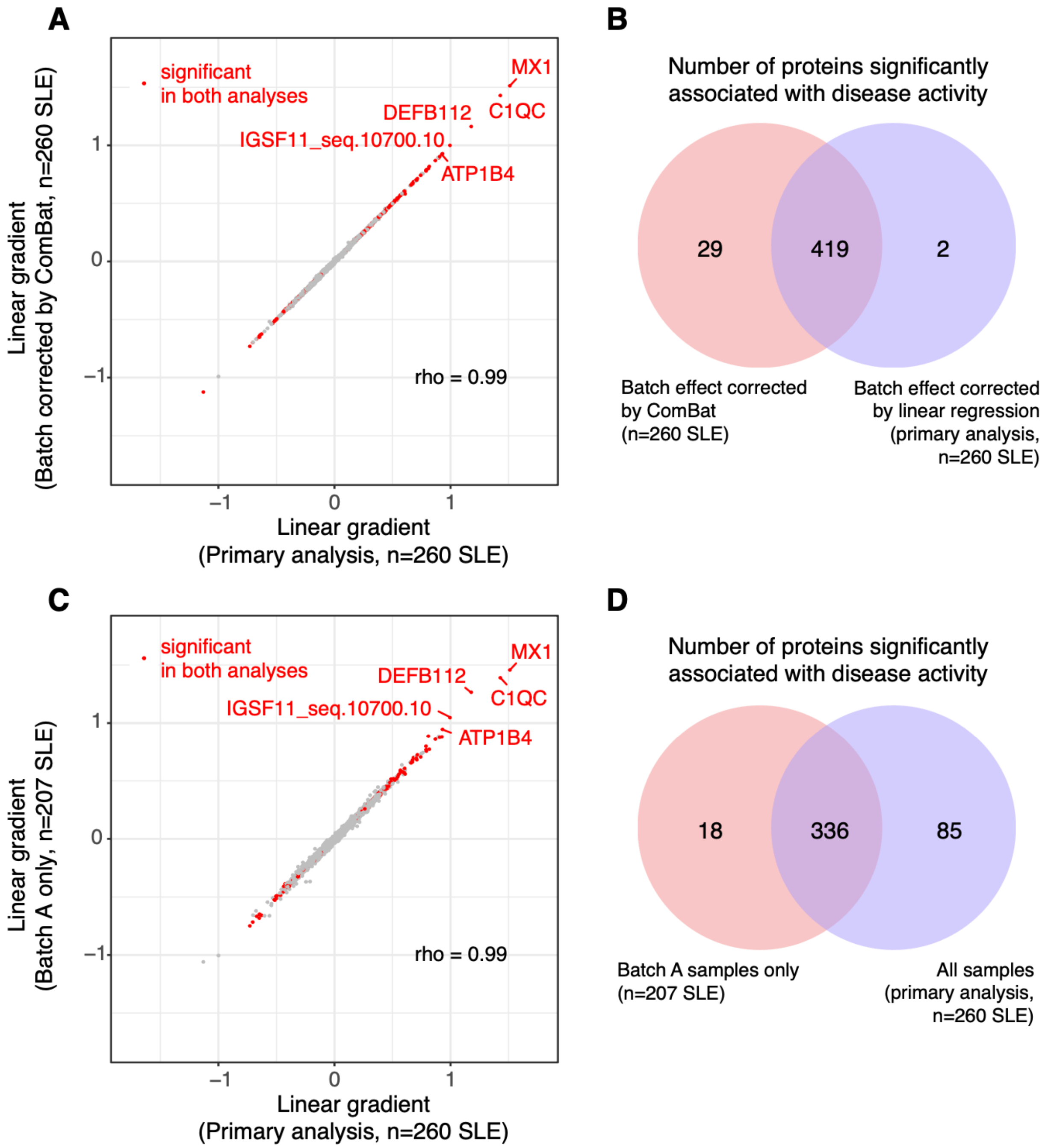

Supplementary Figure 6. Sensitivity analyses for disease activity-associated proteins analysis evaluating effect of batch correction strategies. A) Comparison of effect sizes obtained from primary analysis (batch adjusted by inclusion of covariate in linear regression model) versus using ComBat-corrected proteomic data. Proteins significant in both analyses are highlighted in red. **B)** Overlap of disease activity-associated proteins identified in the primary analysis versus using ComBat-adjusted data. **C)** Comparison of effect sizes from the primary analysis and an analysis restricted to Batch A samples only (n=207). **D)** Overlap of disease activity-associated proteins identified in the primary and Batch A-only analyses.

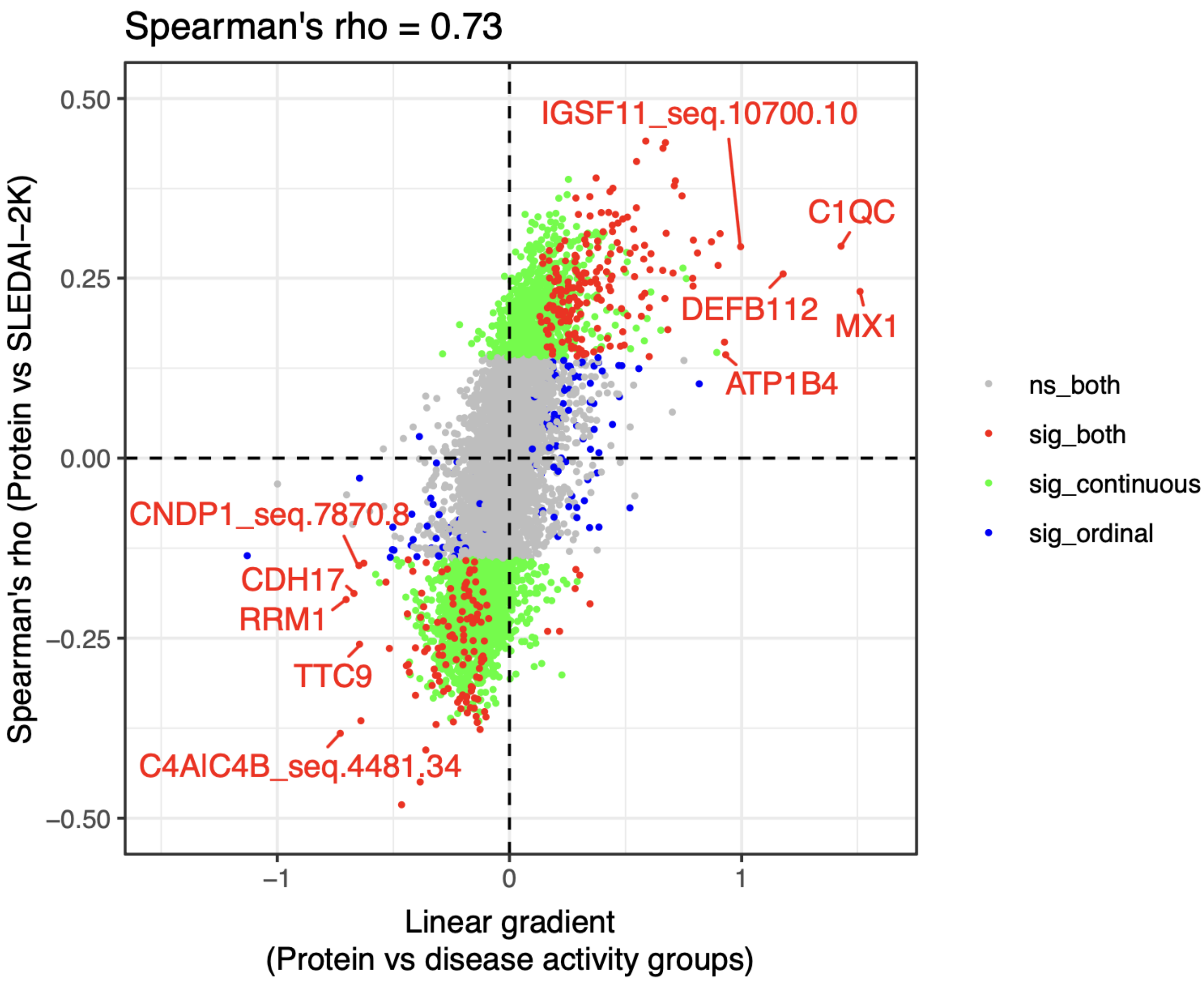

Supplementary Figure 7. Sensitivity analysis treating SLEDAI as a continuous variable. Sensitivity analysis comparing analysing disease activity as a 4-level ordinal variable versus using SLEDAI score as a continuous trait. X-axis: linear gradient obtained from linear regression model treating disease activity as ordinal variable. Y-axis: Spearman's correlation test on protein levels (residuals after adjustment for covariates) vs SLEDAI (as continuous trait). Each point represents a protein analyte and is coloured based on statistical significance - i) not significant (ns, FDR < 0.05) in both models (grey); ii) significant in both models (red); iii) significant only in the Spearman's test (green); or iv) significant only in the linear regression model (blue).

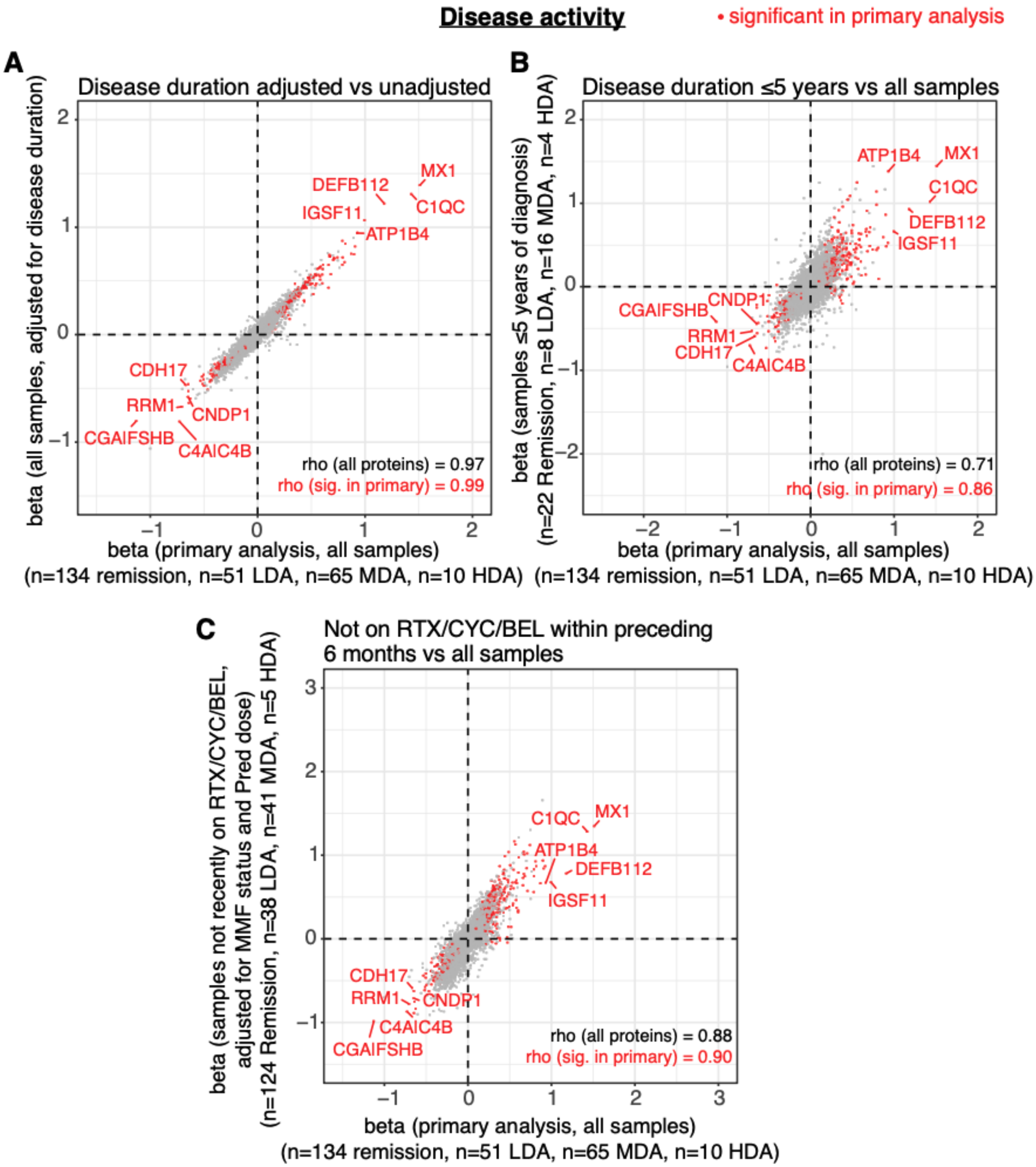

Supplementary Figure 8. Sensitivity analyses for testing proteins for association with disease activity. Comparison of the primary analysis using all available samples versus **(A)** with the inclusion of disease duration as an additional covariate, **(B)** restricting to samples taken within 5 years of SLE diagnosis, and **(C)** excluding samples from patients who received Rituximab, Cyclophosphamide, or Belimumab within the preceding 6 months, and additionally adjusted for MMF treatment status and Prednisolone dose. Red and grey, significant and not significant in primary analysis, respectively.

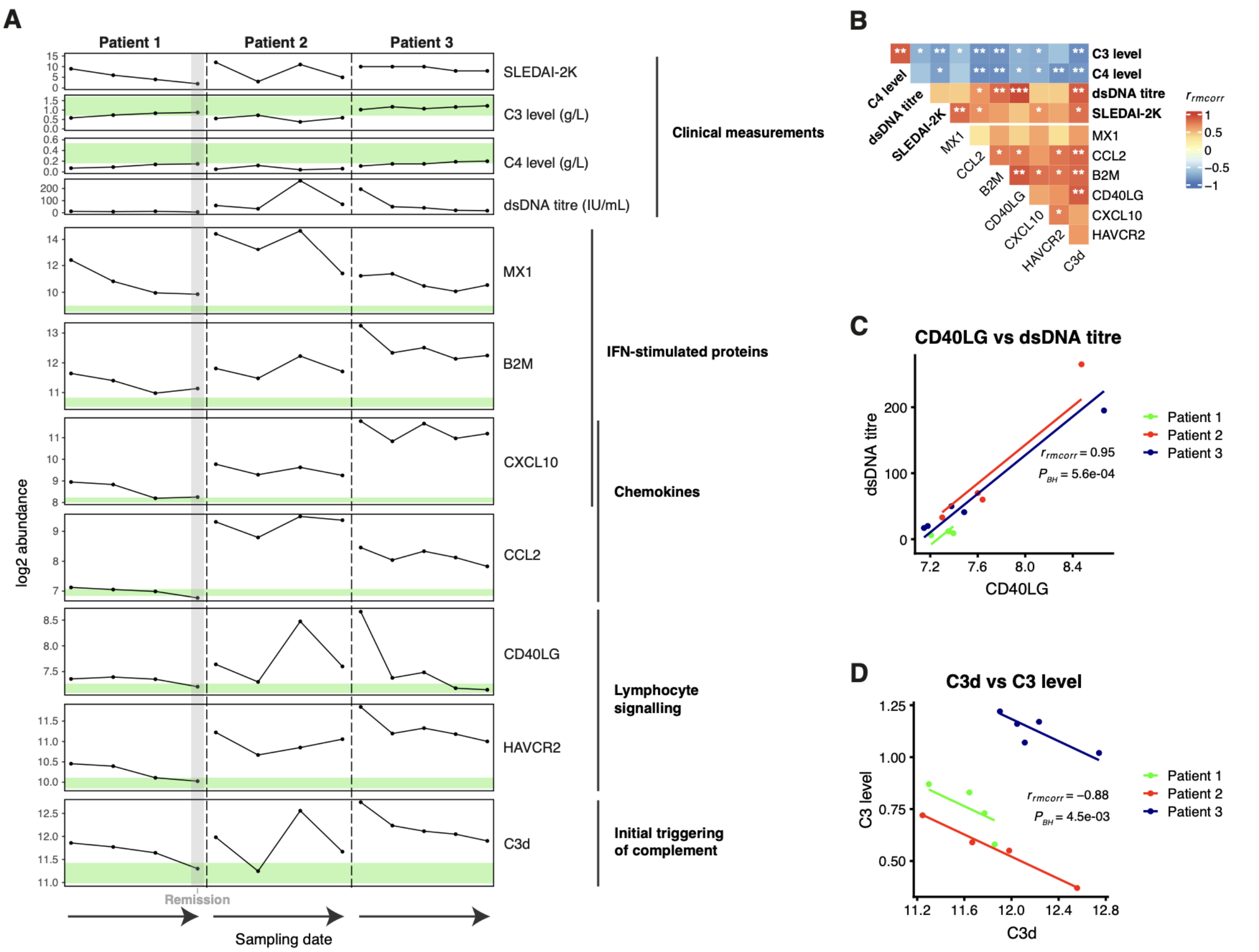

Supplementary Figure 9. Disease activity-associated proteins in serial samples collected from 3 SLE patients. A) Upper panel: SLEDAI-2K and clinically used disease activity biomarkers. Lower panel: plasma proteins measured using SOMAScan. Each point represents a sample. Samples were ordered by sampling date (from the oldest to the most recent one) within each patient. For clinical laboratory measured C3 and C4 levels, the green colour represents their normal reference range. For SOMAScan-measured plasma proteins, normal range was estimated using the median±interquartile range from the healthy volunteers. Proteins were grouped by their biological functions. The grey vertical shading area highlights the sample of Patient 1 taken during remission. Proteins B2M and HAVCR2 had multiple SOMAmers on the SOMAScan; in this figure B2M corresponds to measurement with SOMAmer "seq.3485.28" and HAVCR2 to "seq.5134.52". **B)** Repeated measures correlations between clinical parameters (shown in bold font) and levels of SOMAScan-measured plasma proteins (non-bold) in all serial samples of the 3 patients. Red and blue represent positive and negative correlations, respectively. *, $P_{BH}<0.05$; **, $P_{BH}<0.01$; ***, $P_{BH}<0.001$. **C-D)** Intra-individual evaluation of longitudinal correlation between **C)** plasma CD40LG and anti-dsDNA autoantibody levels, and **D)** plasma C3d (SOMAScan) versus C3 levels (clinical laboratory measure).

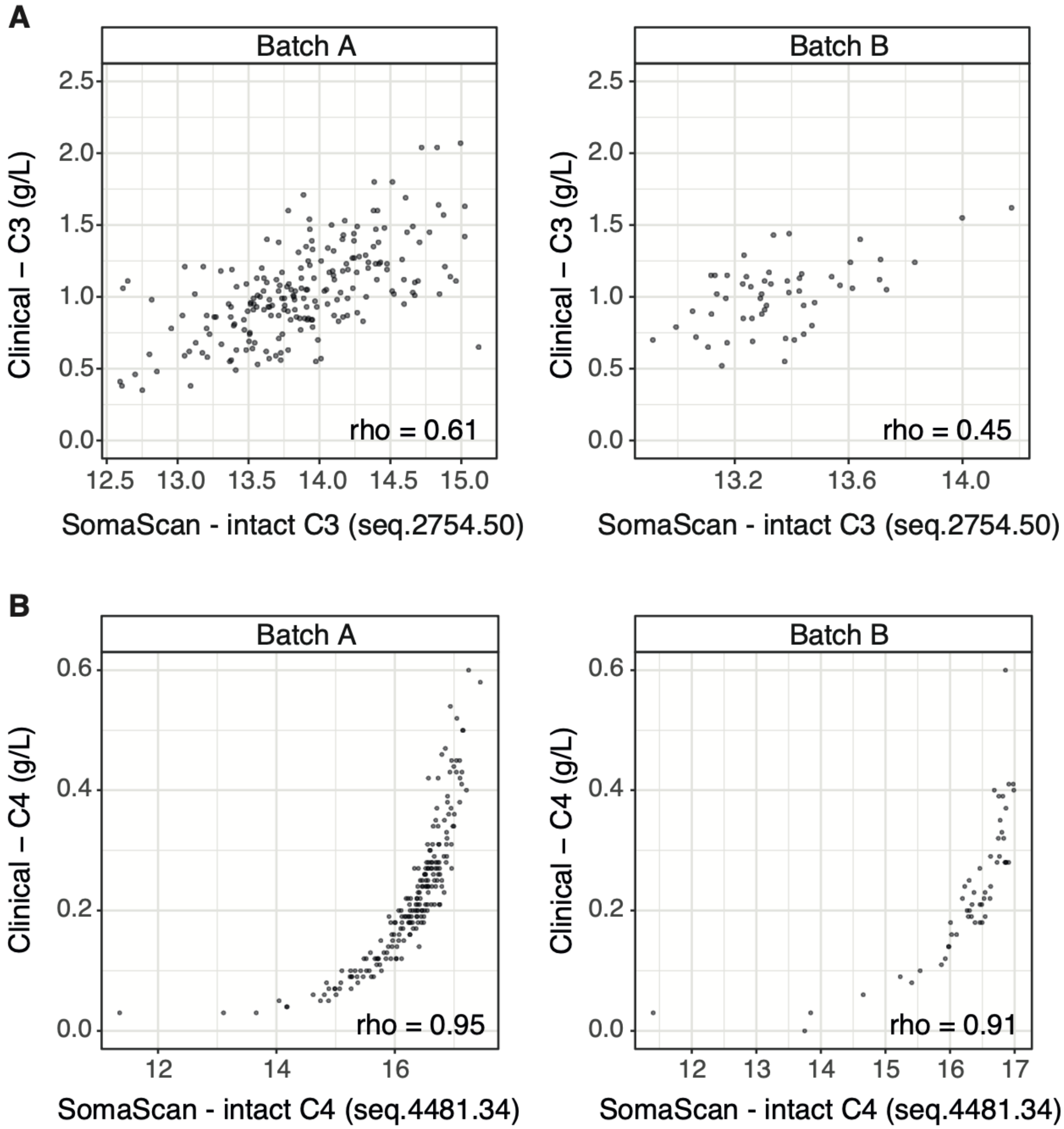

Supplementary Figure 10. Correlation of SomaScan- and clinically measured intact complement levels. A) Correlations between clinically measured C3 levels and SomaScan-measured intact C3 levels (measured by SOMAmer seq.2754.50) in both batches of data. **B)** Correlations between clinically measured C4 levels and SomaScan-measured intact C4 levels (measured by SOMAmer seq.4481.34) in both batches of data.

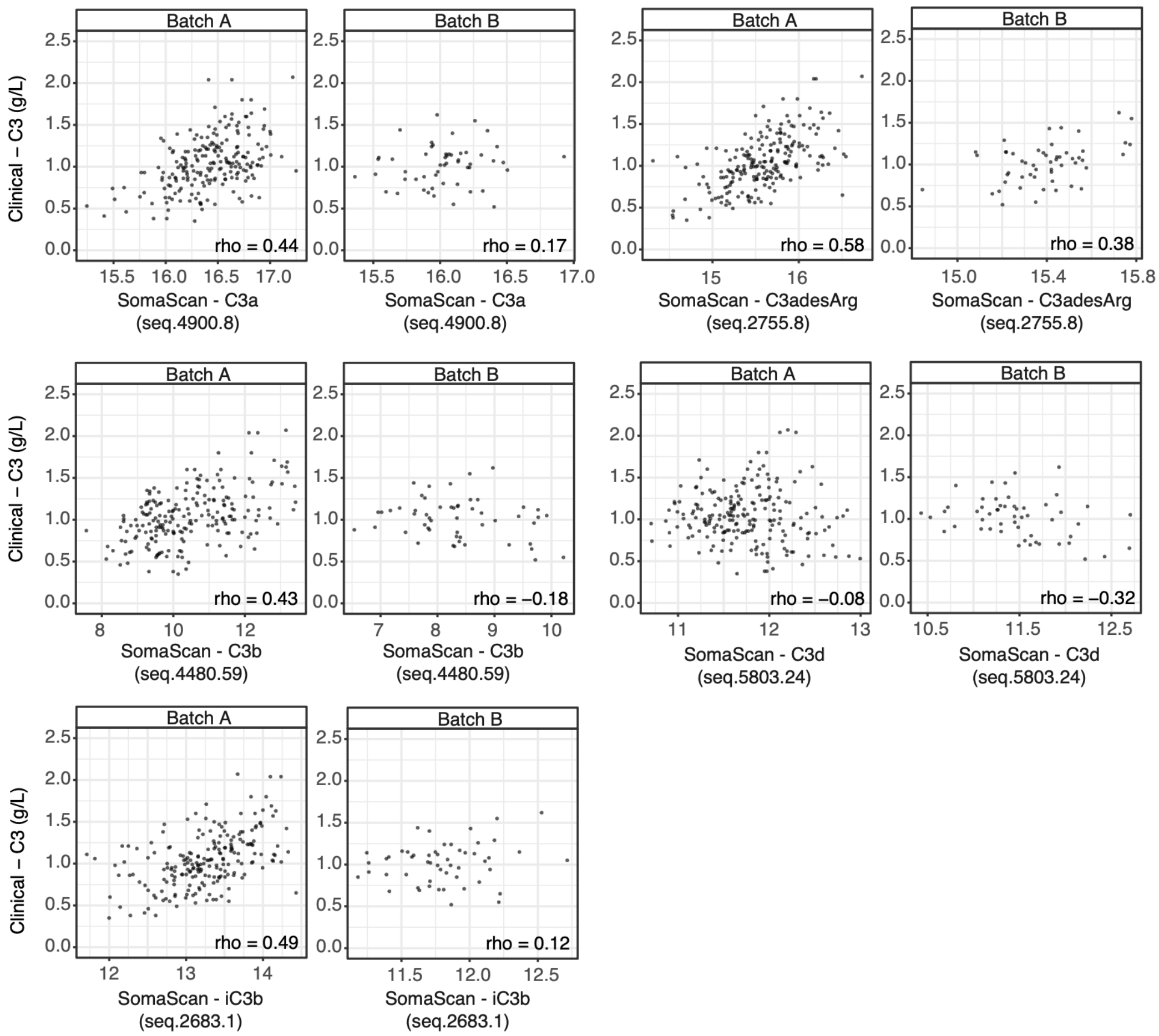

Supplementary Figure 11. Complement cleavage fragments of C3 measured in SomaScan and their correlations with clinically measured intact C3. Five SOMAmers for C3 fragments were measured in SomaScan, targeting C3a (seq.4900.8), C3adesArg (seq.2755.8), C3b (seq.4480.59), C3d (seq.5803.24), and iC3b (seq.2683.1). Data are presented stratified by batch.

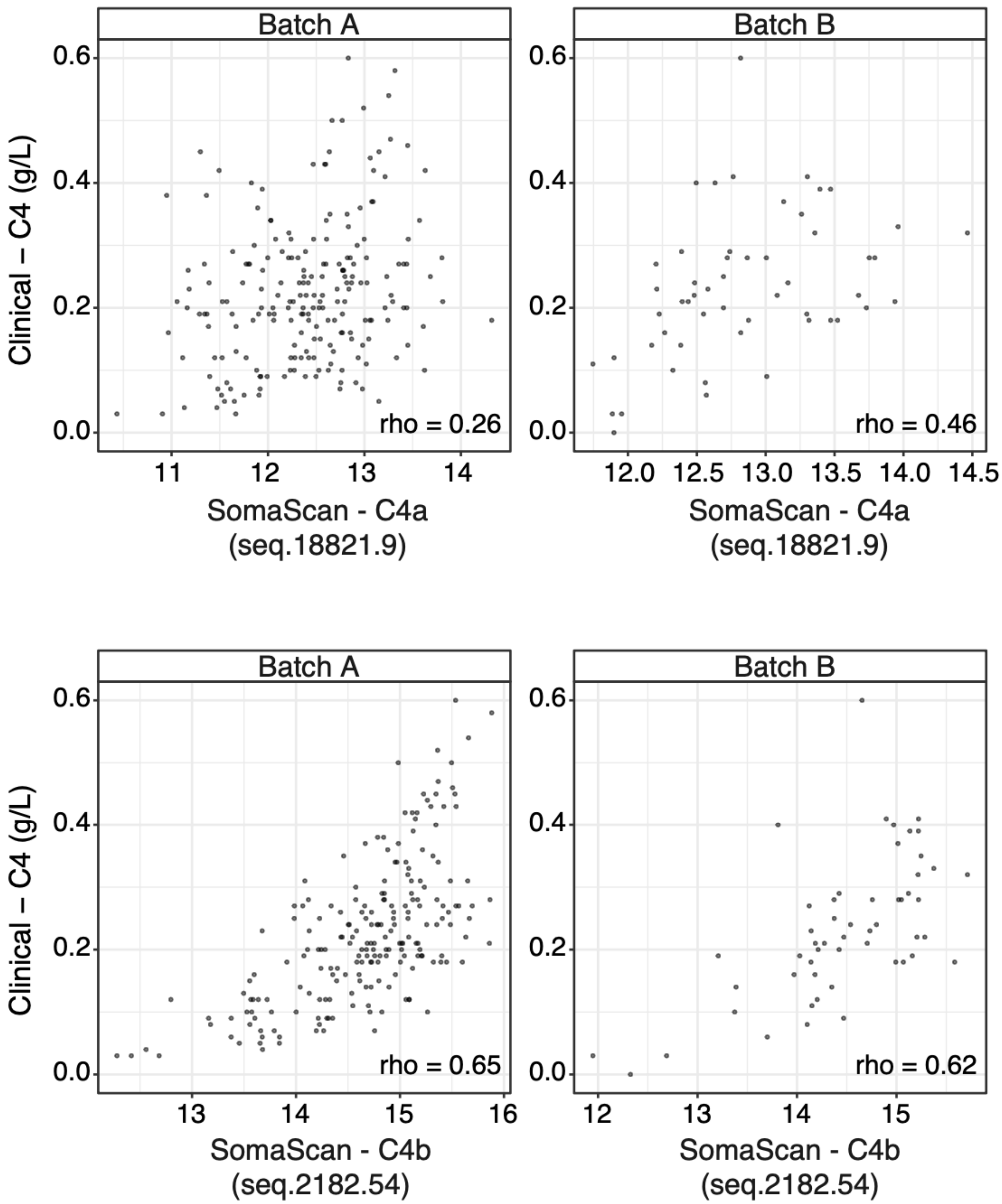

Supplementary Figure 12. Complement cleavage fragments of C4 measured in SomaScan and their correlations with clinically measured C4. Two SOMAmers for C4 fragments were measured in SomaScan, targeting C4a (seq.18821.9), and C4b (seq.2182.54). Data are presented stratified by batch.

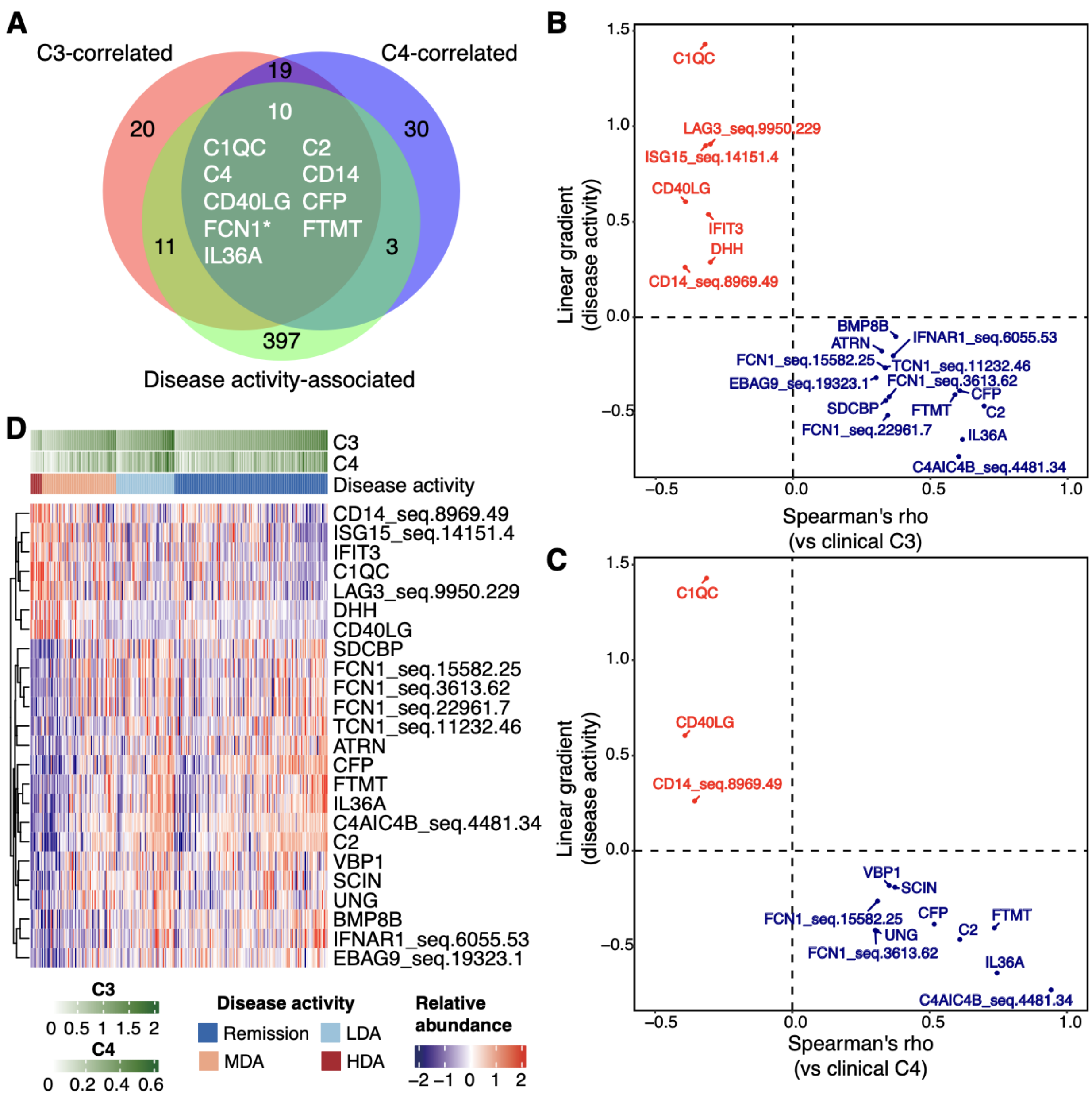

Supplementary Figure 13. Proteins associated with disease activity and clinically measured C3 or C4. A) Venn diagrams of the number of protein analytes significantly correlated with clinical C3 and C4 levels, and associated with disease activity. Ten SOMAmers correlated with both C3 and C4 and associated with activity were listed. *These include two SOMAmers for FCN1 (seq.15582.25 and seq.3613.62). **B)** Proteins associated with disease activity and with clinical lab-measured C3 levels. **C)** Proteins associated with disease activity and correlated with clinical lab-measured C4. Red: proteins positively associated with disease activity, Blue: proteins negatively associated with disease activity. **D)** Proteins associated with disease activity and with either clinical lab-measured C3 or C4. Protein levels were adjusted by sex and batch.

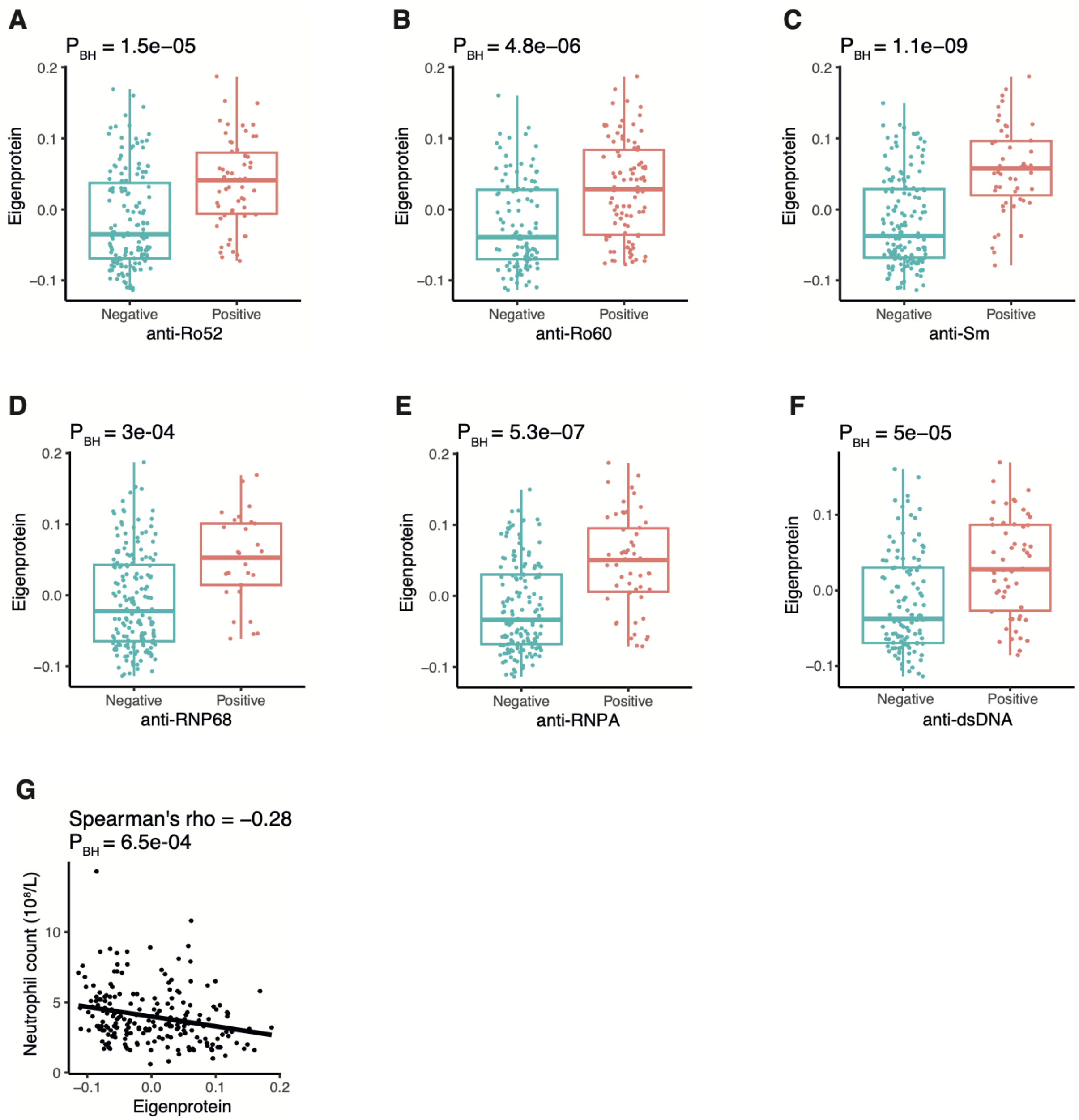

Supplementary Figure 14. Clinical traits that were significantly associated with the red module identified by WGCNA. All traits that were strongly associated (FDR < 0.001) with the modular eigenprotein values of the red module were shown, including 6 binary traits of autoantibodies status: **A)** anti-Ro52, **B)** anti-Ro60, **C)** anti-Sm, **D)** anti-RNP68, **E)** anti-RNPA, **F)** anti-dsDNA, and 1 continuous trait: **G)** neutrophil count. P_{BH}, Benjamini-Hochberg-corrected P value.

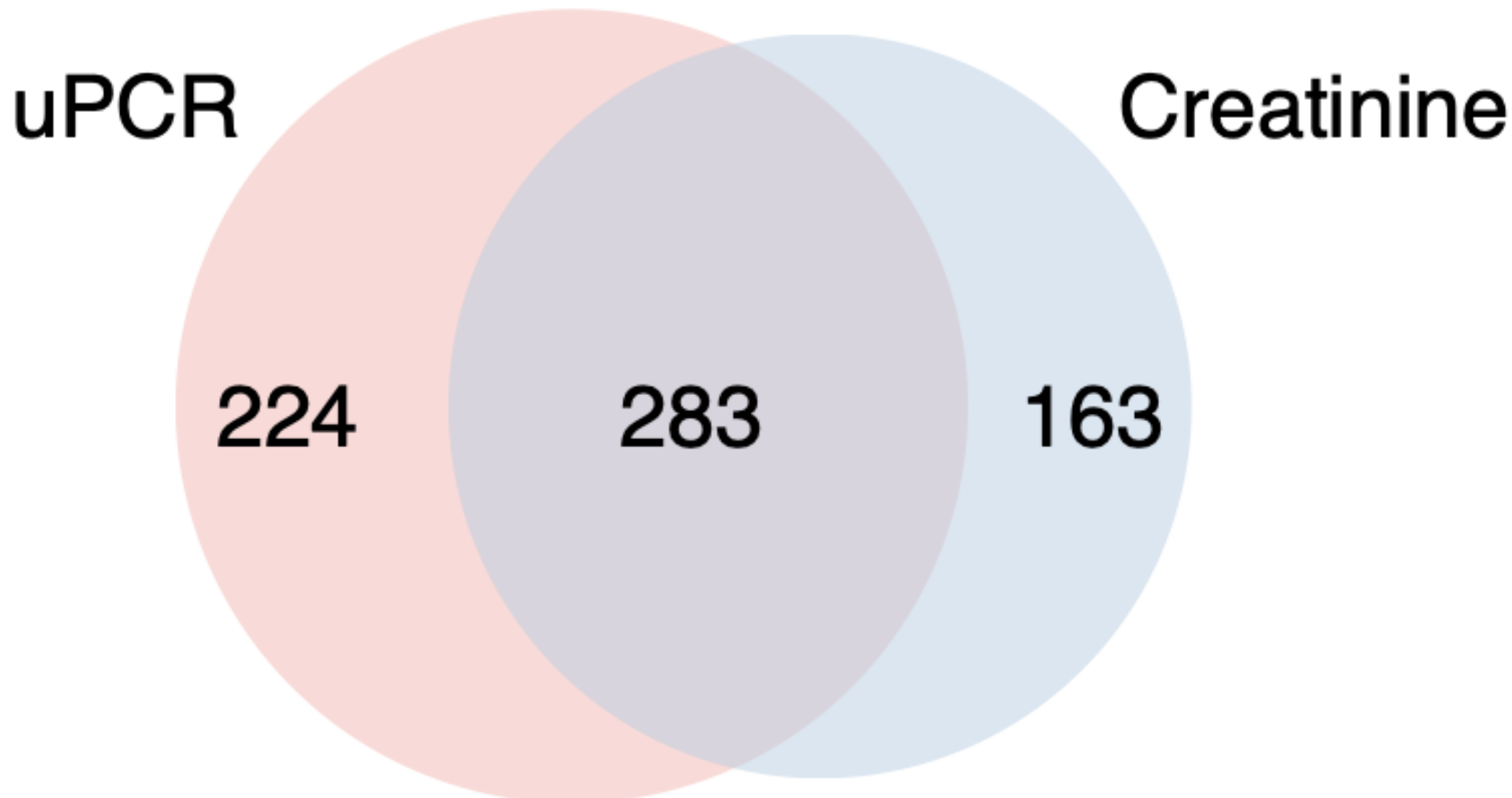

Supplementary Figure 15. Venn diagram of the number of proteins significantly correlated with serum uPCR and creatinine levels. Correlation defined as unsigned rho >0.3 and P_{BH} <0.05.

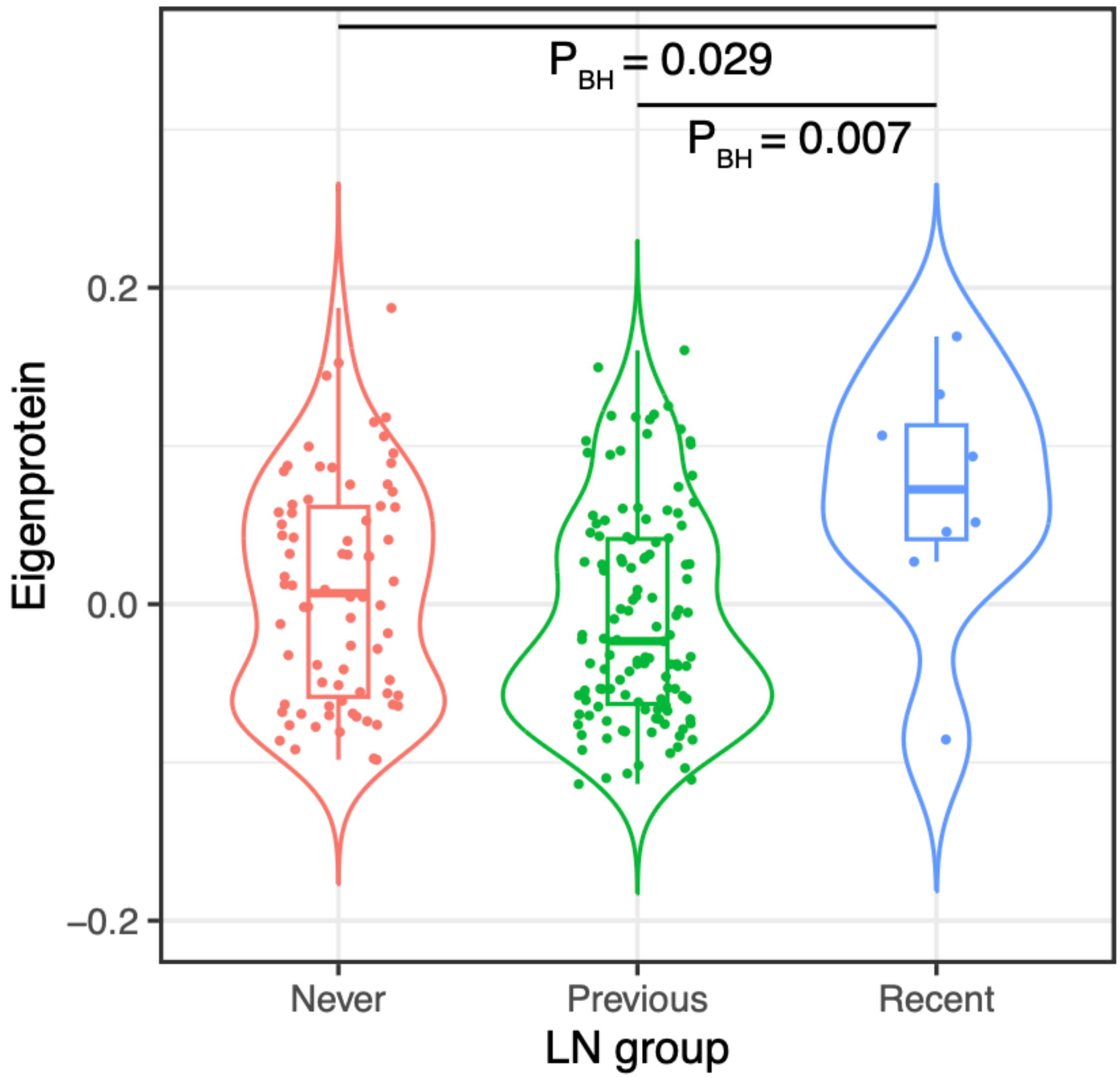

Supplementary Figure 16. Association between the IFN-associated protein module and lupus nephritis (LN) status. Violin and box plots showing eigenprotein values across LN groups (Never, Previous, and Recent LN). Each point represents an individual sample. Significant pairwise comparisons are indicated with Benjamini-Hochberg-adjusted P values (P_{BH}).

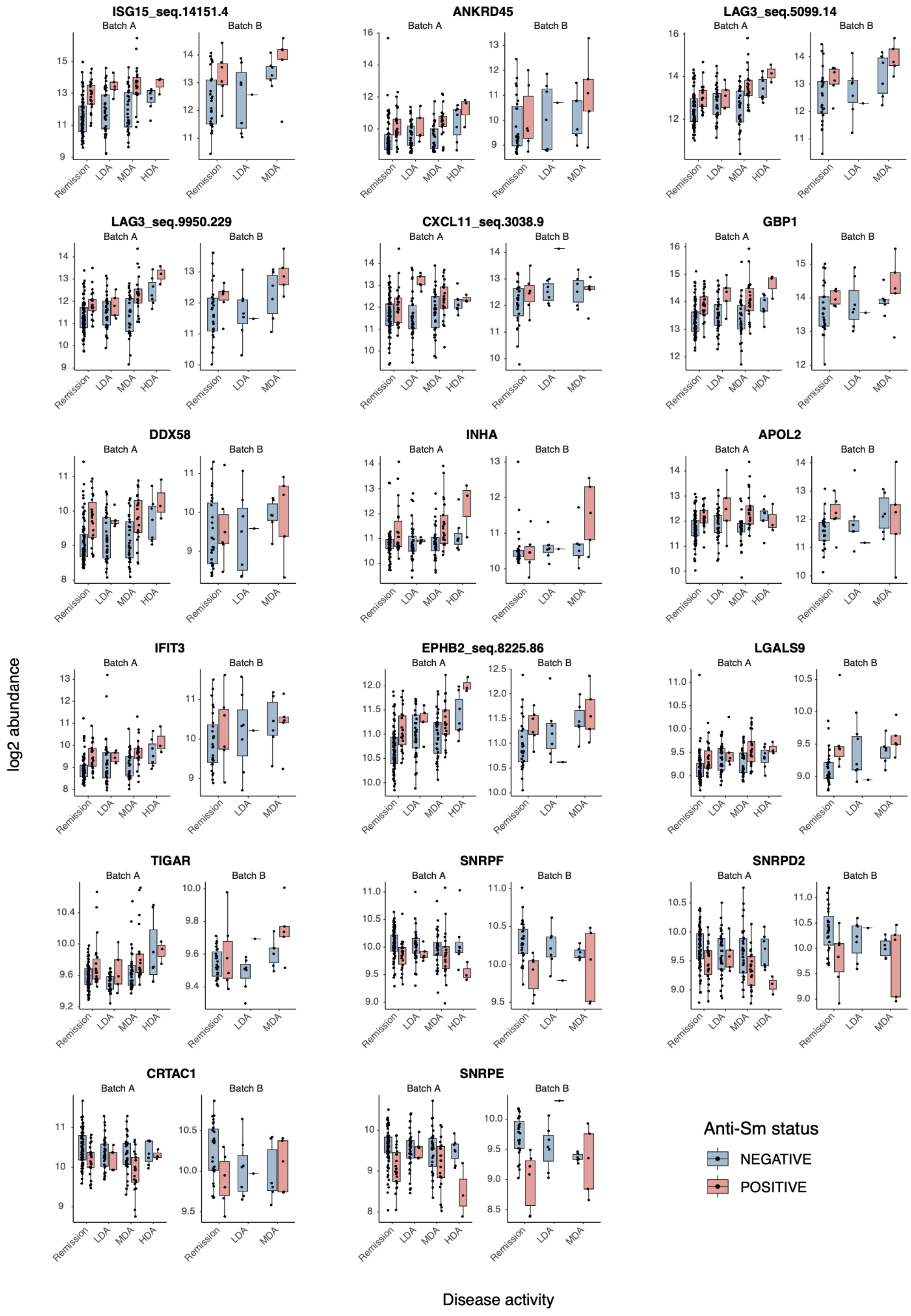

Supplementary Figure 17. Proteins associated with anti-Sm autoantibodies independent of disease activity. Boxplots showing the protein levels of anti-Sm-associated proteins (excluding 3 proteins that were displayed in Figure 4C) stratified by batch and disease activity. For clarity of visualisation, only the proteins associated with anti-Sm positivity at 1% FDR were shown. A full list of anti-Sm associated proteins is provided in Supplementary Data 14.

Anti-Sm(+) vs Anti-Sm(-)

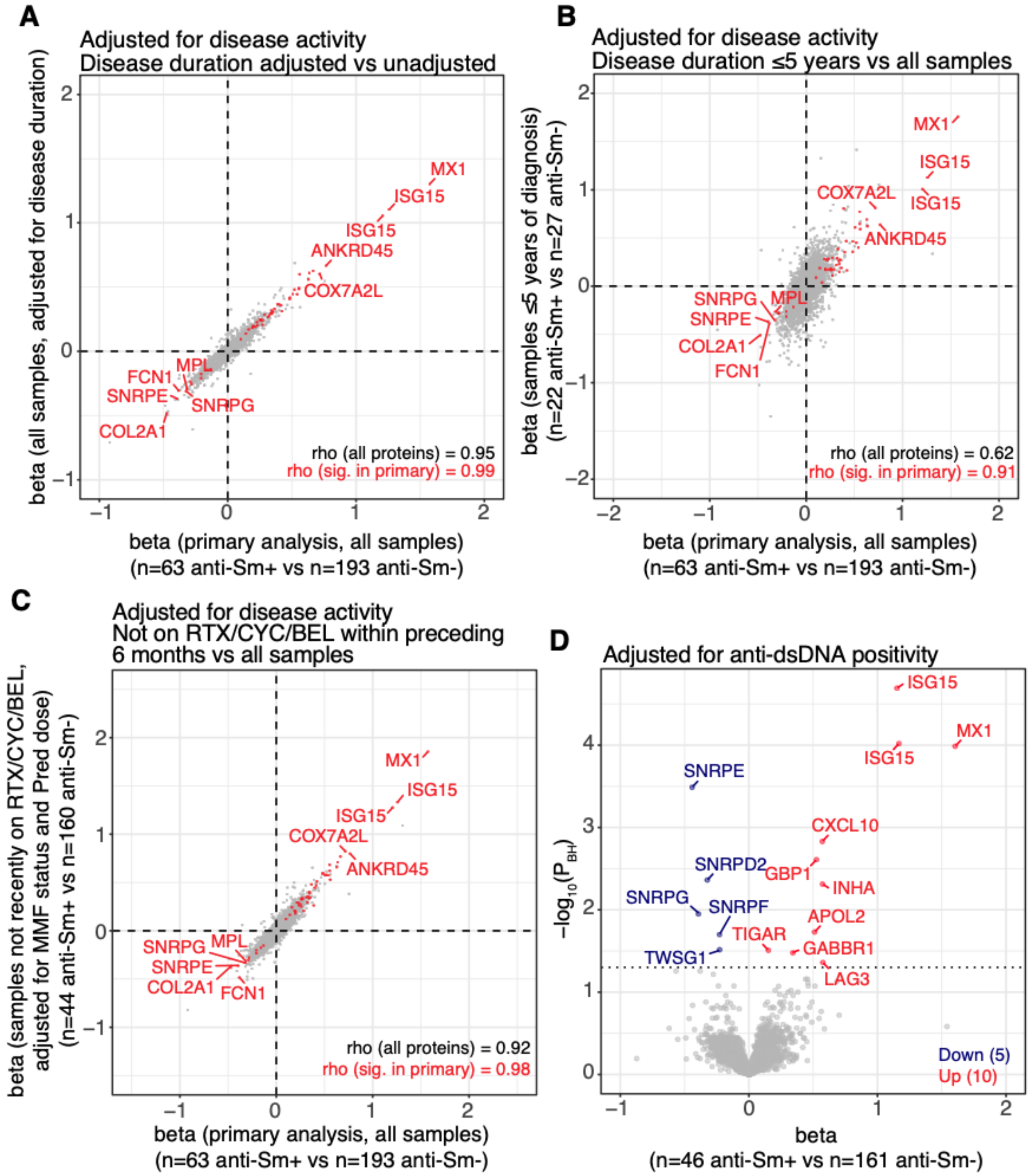

Supplementary Figure 18. Sensitivity analyses of anti-Sm(+) vs anti-Sm(-). Comparison of the primary analysis of anti-Sm(+) vs anti-Sm(-) using all available samples versus **(A)** with the inclusion of disease duration as an additional covariate in the regression models, **(B)** restricting to samples taken within 5 years of SLE diagnosis, and **(C)** excluding samples from patients who had received Rituximab, Cyclophosphamide or Belimumab within the previous 6 months and statistically adjusting for MMF treatment status and Prednisolone dose. Red and grey, significant and not significant in primary analysis, respectively. For **A-C,** disease activity was included as a covariate. **D.** Differential abundance analysis of n=46 anti-Sm(+) vs n=161 anti-Sm(-) SLE after adjusting for anti-dsDNA antibody positivity. Red, upregulated; Blue, downregulated. P_{BH}, Benjamini-Hochberg-corrected P value.

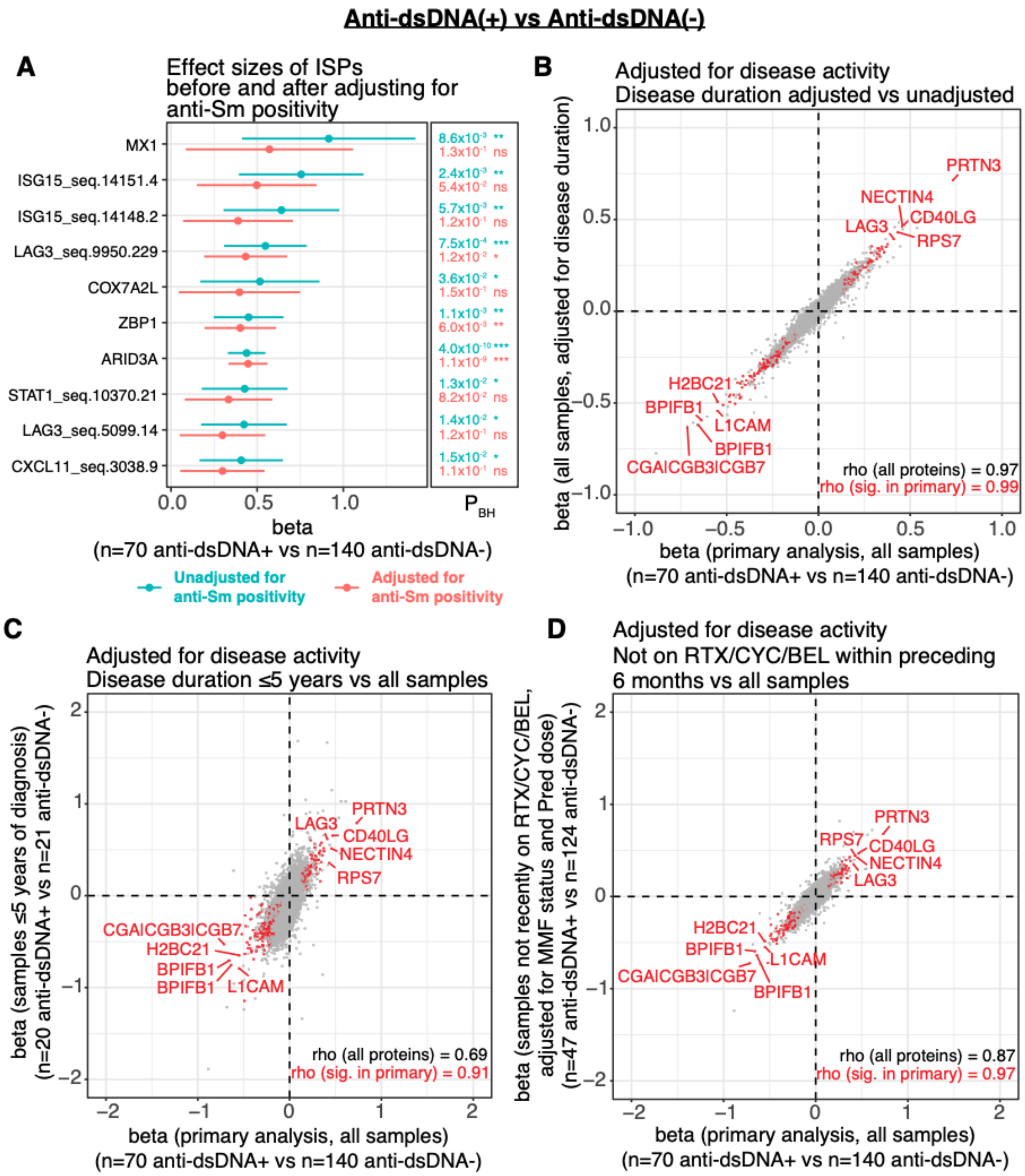

Supplementary Figure 19. Sensitivity analyses of anti-dsDNA(+) vs anti-dsDNA(-). A. Effect sizes (log_2 fold change) of the ten most differentially abundant ISPs in the analysis of anti-dsDNA(+) vs anti-dsDNA(-) SLE unadjusted and adjusted for anti-Sm autoantibody positivity. Error bars represent the 95% confidence intervals (not accounting for multiple testing). P-values have been adjusted for multiple testing. * $P_{BH}<0.05$; **, $P_{BH}<0.01$; ***, $P_{BH}<0.001$. ns, not significant at FDR<0.05. **B-D.** Comparison of the primary analysis of anti-dsDNA(+) vs anti-dsDNA(-) using all available samples versus **(B)** with the inclusion of disease duration as an additional covariate in the regression models, **(C)** restricting to samples taken within 5 years of SLE diagnosis, and **(D)** excluding samples from patients who received Rituximab, Cyclophosphamide, or Belimumab in the preceding 6 months and additionally adjusted for MMF status and Prednisolone dose. Red and grey, significant and not significant in primary analysis, respectively. P_{BH}, Benjamini-Hochberg-corrected P value.

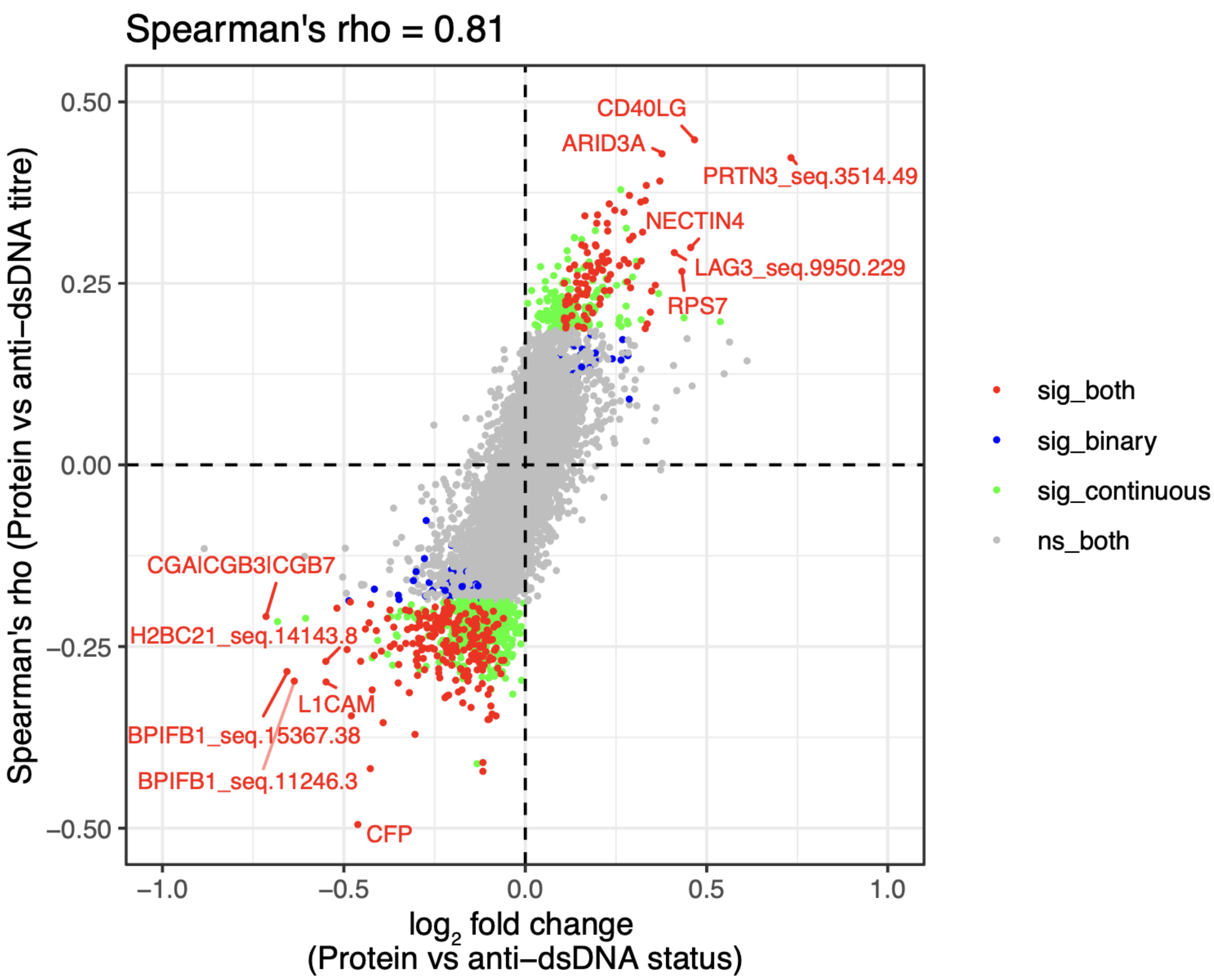

Supplementary Figure 20. Sensitivity analysis treating anti-dsDNA titre as a continuous variable. Sensitivity analysis was performed using Spearman's correlation test on protein levels vs anti-dsDNA titre (as a continuous trait) to obtain Spearman's rho (Y-axis) and compared with effect size (adjusted $\log_2$FC) obtained from linear regression (X-axis, treating anti-dsDNA status as binary outcome). Protein levels were adjusted for batch, sex, and disease activity in both analyses. Each point represents a protein and is coloured based on their statistical significance - i) not significant (ns) in both models (grey); ii) significant in both models (red); iii) significant only in the linear regression model (blue); or iv) significant only in the Spearman's test (green).

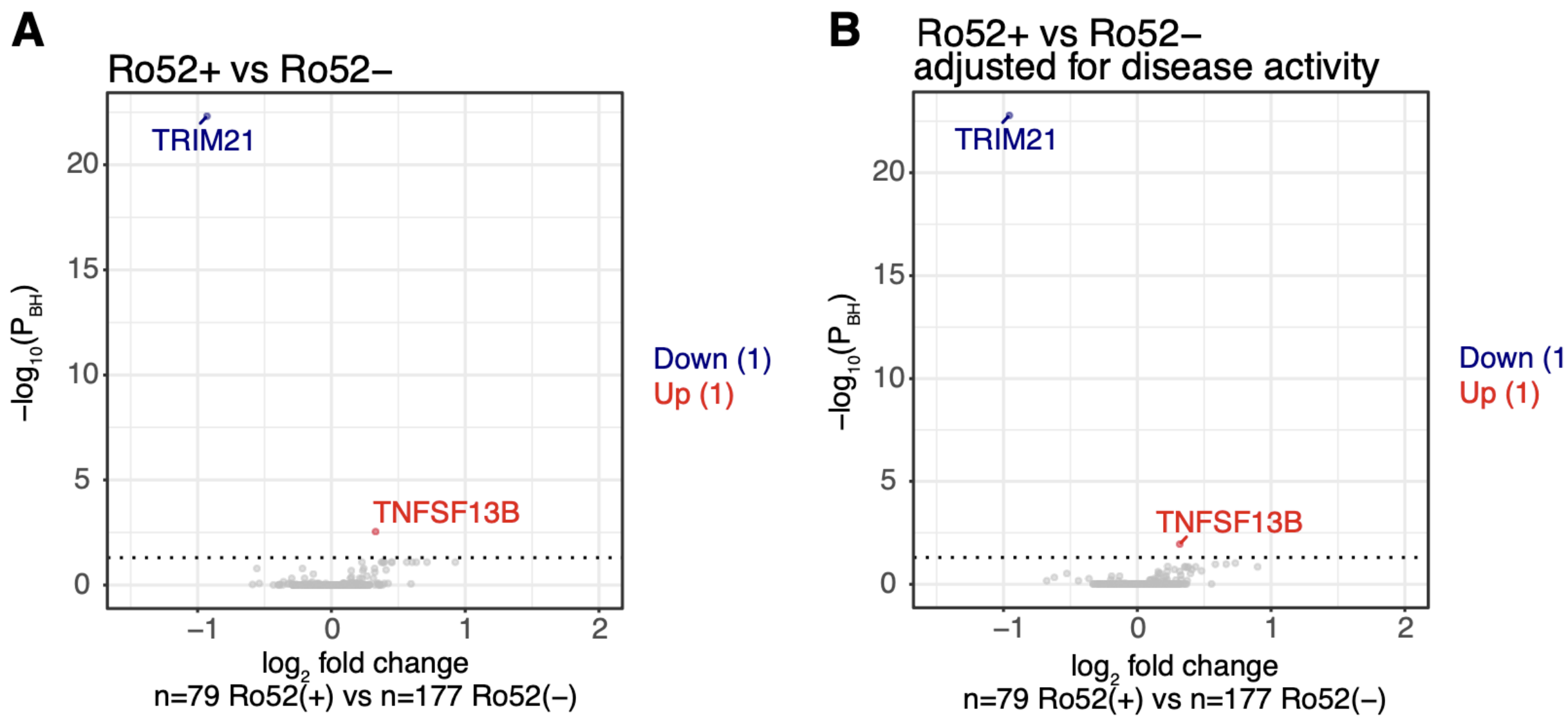

Supplementary Figure 21. Proteins associated with anti-Ro52 autoantibodies. A. Differential abundance analysis comparing n=79 anti-Ro52(+) and n=177 anti-Ro52(-) SLE. **B.** As in **A** but adjusted for disease activity. Batch and sex were adjusted as covariates in both analyses. Red, upregulated proteins; Blue, downregulated proteins. P_{BH}, Benjamini-Hochberg-corrected P value. Proteins annotated using non-italicised symbol of the encoding gene. TRIM21 is another name for the Ro52 antigen.

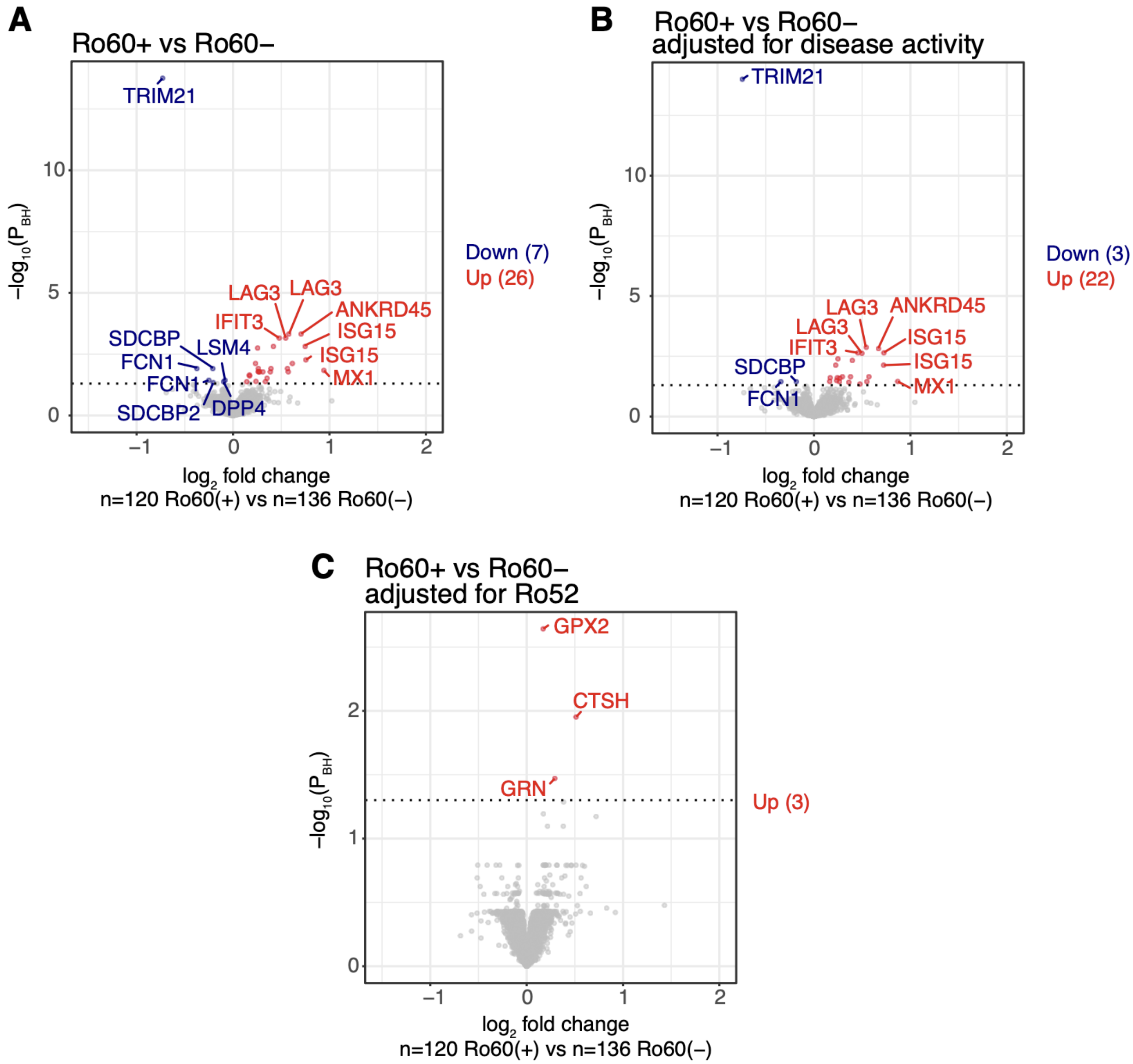

Supplementary Figure 22. Proteins associated with anti-Ro60 autoantibodies. A. Differential abundance analysis comparing n=120 anti-Ro60(+) and n=136 anti-Ro60(-) SLE. **B.** As in **A** but adjusted for disease activity. **C.** As in **A** but adjusted for anti-Ro52 antibody positivity. Batch and sex were adjusted as covariates in all analyses. Red, upregulated proteins; Blue, downregulated proteins. P_{BH}, Benjamini-Hochberg-corrected P value.

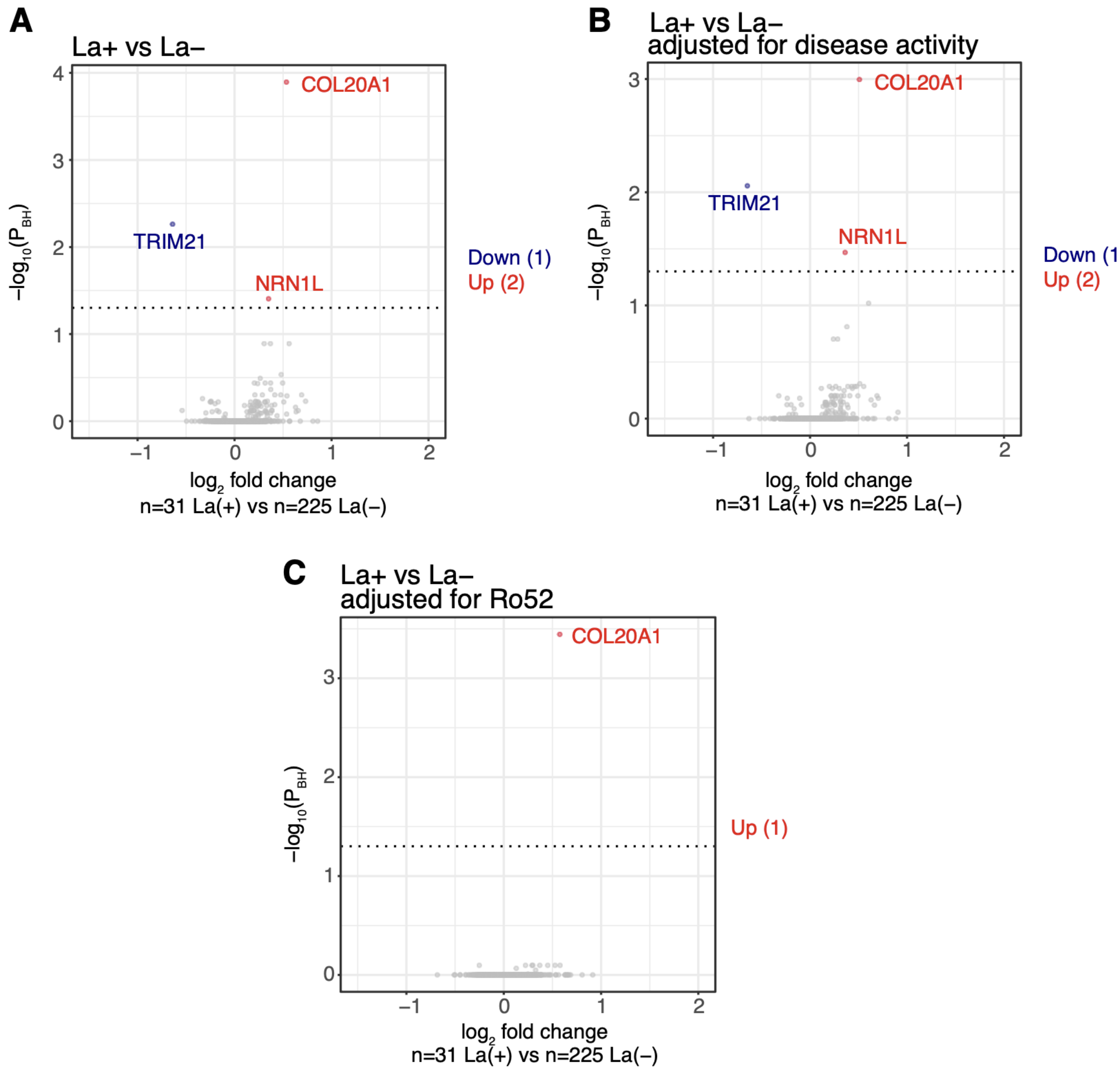

Supplementary Figure 23. Proteins associated with anti-La autoantibodies. A. Differential abundance analysis comparing n=31 anti-La(+) and n=225 anti-La(-) SLE. **B.** As in **A** but adjusted for disease activity. **C.** As in **A** but adjusted for anti-Ro52 antibody positivity. Batch and sex were adjusted as covariates in all analyses. Red, upregulated proteins; Blue, downregulated proteins. P_{BH}, Benjamini-Hochberg-corrected P value.

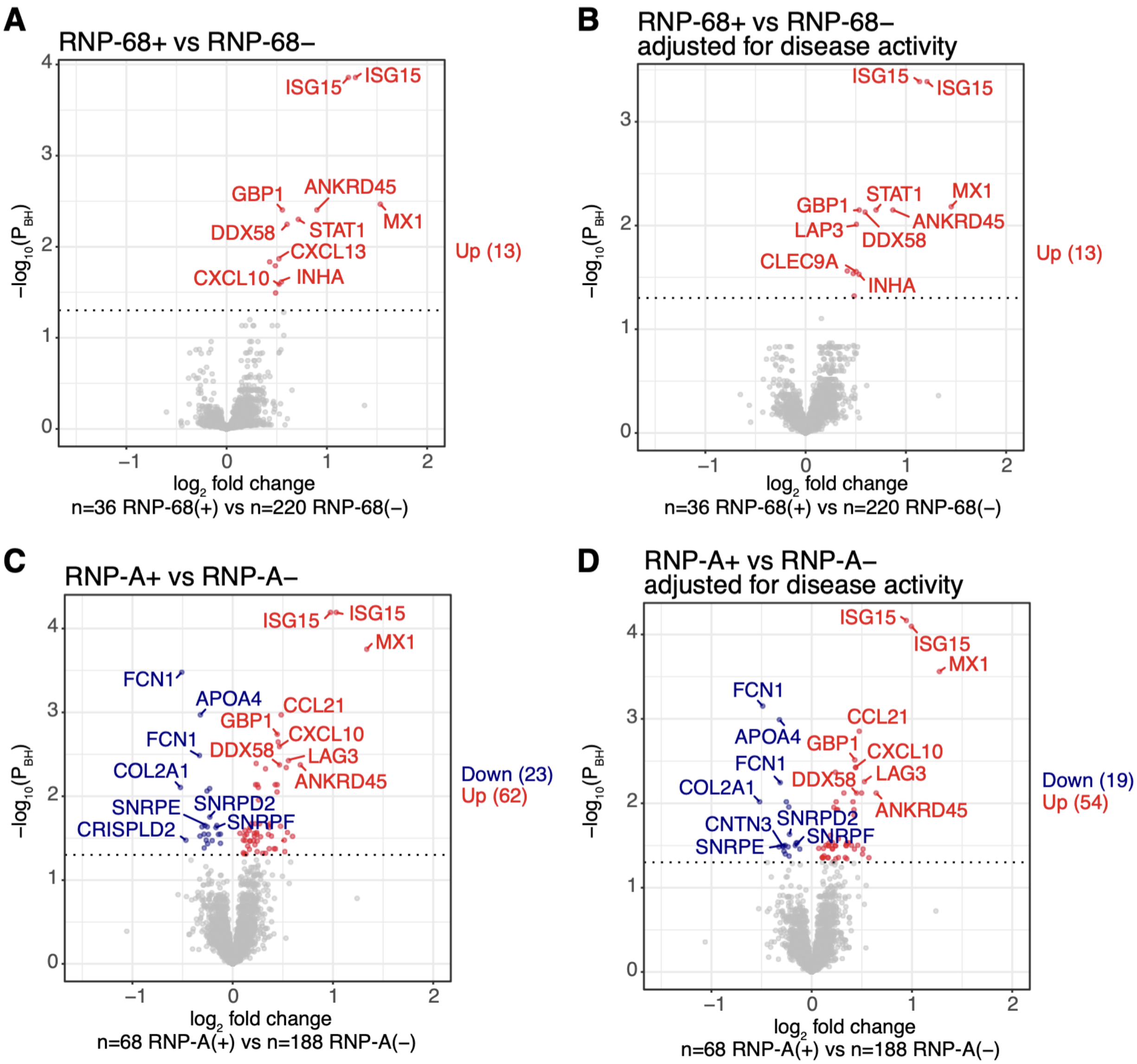

Supplementary Figure 24. Proteins associated with RNP-68 and RNP-A autoantibodies. A. Differential abundance analysis comparing n=36 anti-RNP-68(+) and n=220 anti-RNP-68(-) SLE. **B.** As in **A** but adjusted for disease activity. **C.** Differential abundance analysis comparing n=68 anti-RNP-A(+) and n=188 anti-RNP-A(-) SLE. **D.** As in **C** but adjusted for disease activity. Batch and sex were adjusted as covariates in all analyses. Red, upregulated proteins; Blue, downregulated proteins. P_{BH}, Benjamini-Hochberg-corrected P value.

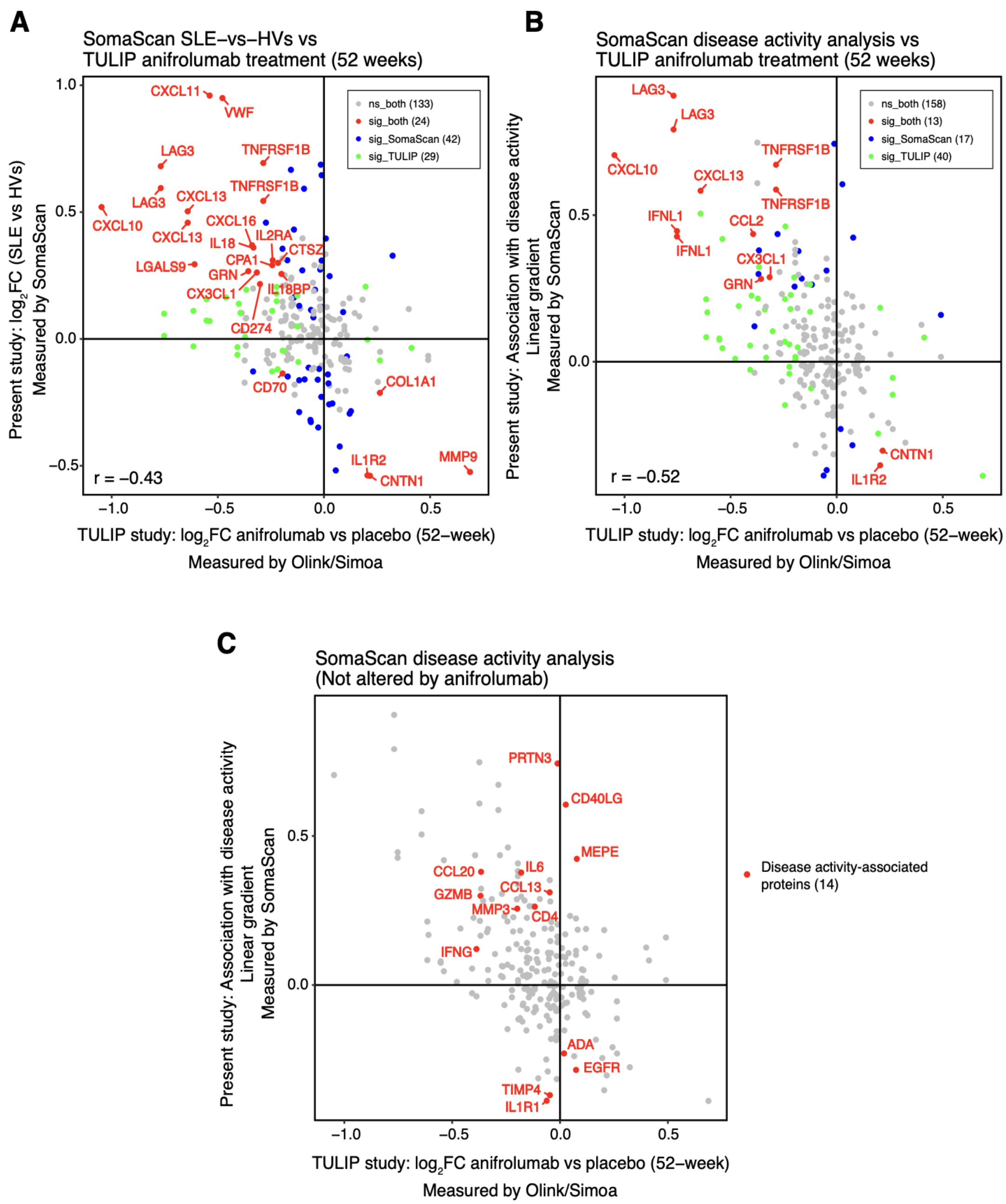

Supplementary Figure 25. Comparison of proteomic changes in SLE with anifrolumab treatment. A) Effect sizes (log_2FC) from our analysis of SLE vs HVs compared with those from the TULIP-1 anifrolumab trial (52 weeks post-anifrolumab vs placebo), showing an inverse correlation (r = -0.43). Proteins dysregulated (FDR < 0.05) in both datasets are highlighted in red. Grey: proteins ns (not significant) in any dataset. Blue: proteins dysregulated in our analysis only. Green: proteins dysregulated in TULIP-1 trial only. **B)** Associations with disease activity in our analysis compared with treatment effects in the TULIP-1 study, showing inverse correlation (r = -0.52). Proteins significant in both datasets are highlighted in red. **C)** Proteins associated with disease activity (FDR <0.05) in our study but were not altered by anifrolumab treatment at nominal P <0.05**.**

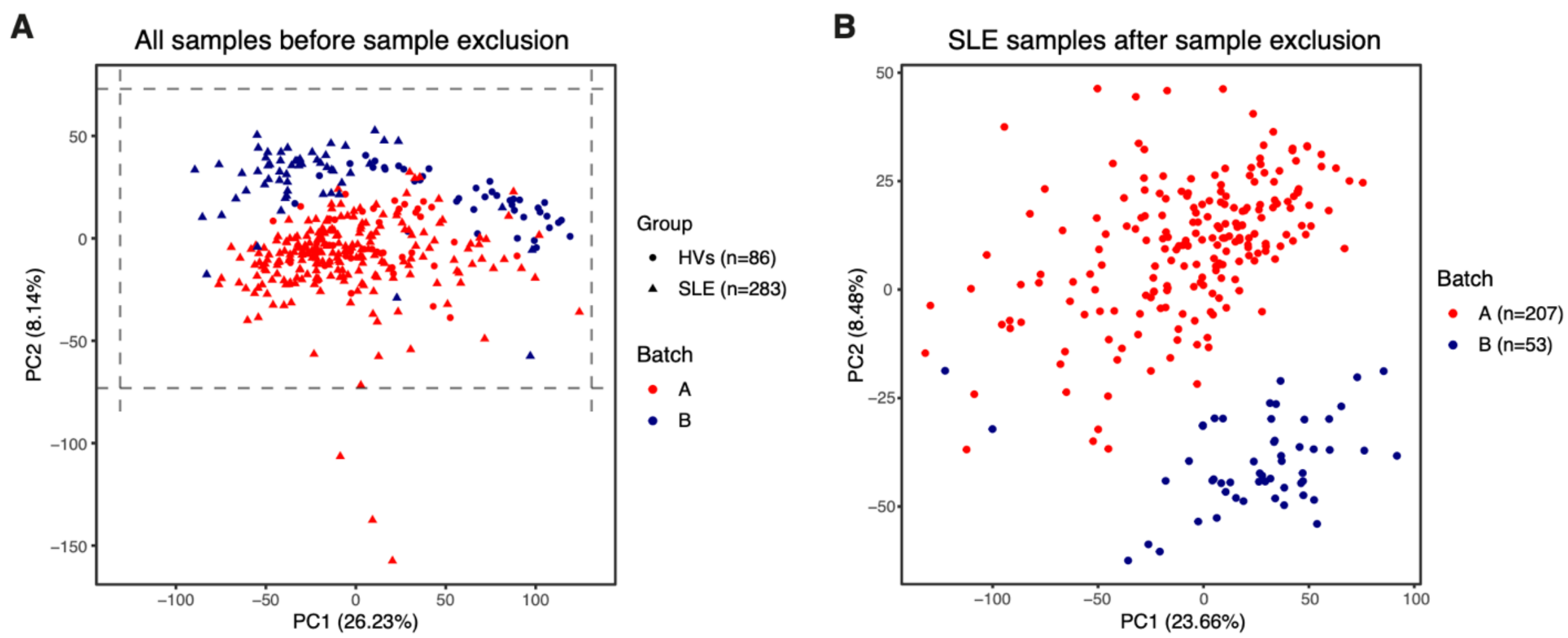

Supplementary Figure 26. Principal components analysis (PCA) of proteomic data. A) PCA plot of SLE and HV samples before outlier removal. Each point represents a sample. Batch is indicated by colour and disease status by shape. Dashed lines indicate the range of ±3 SD from the mean of PC1 and PC2. Three outlier samples outside the dashed lines were removed for downstream analyses. **B)** PCA plot restricted to SLE samples (after removal of outlier samples). Red: Batch A samples, Blue: Batch B samples.

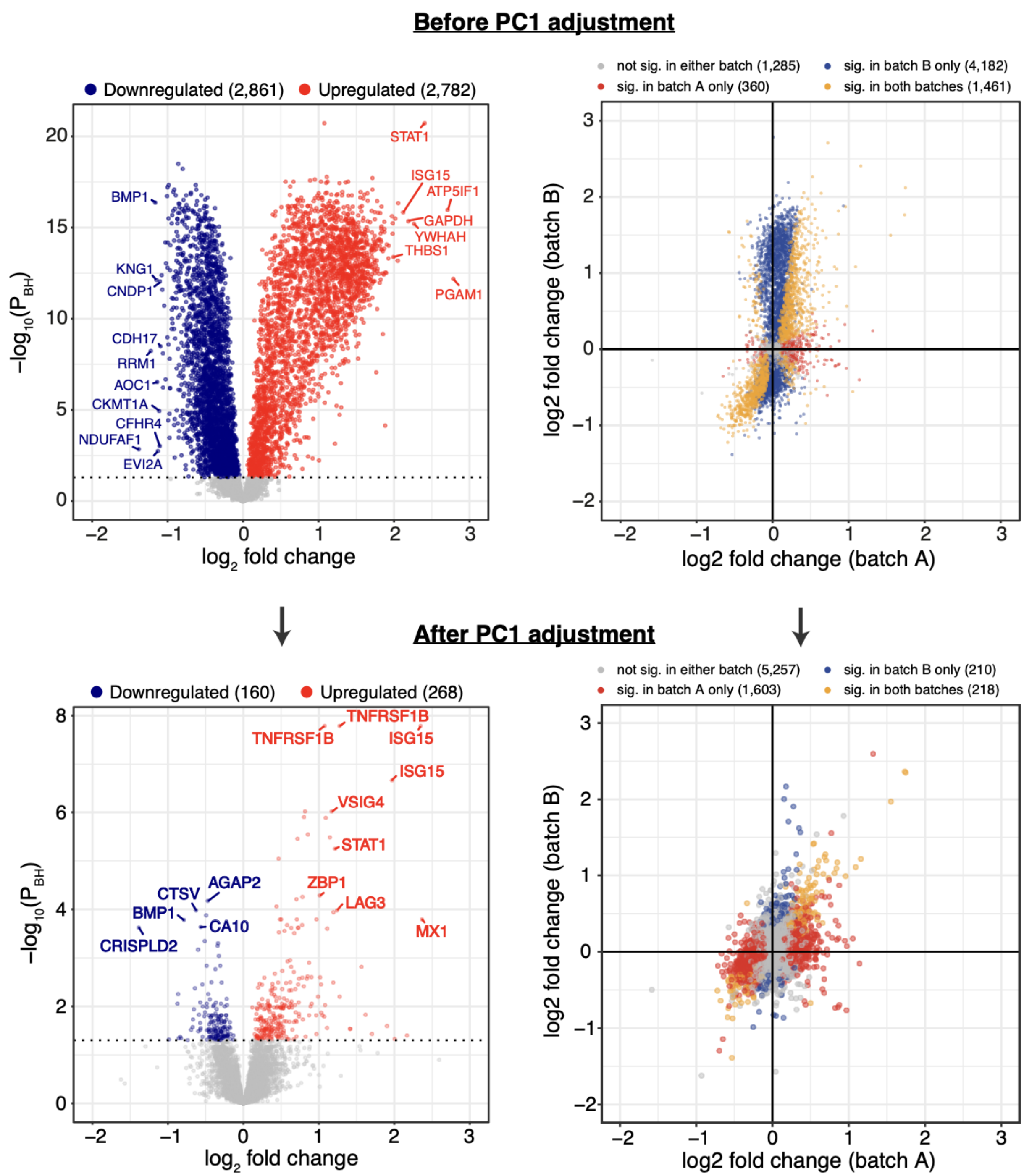

Supplementary Figure 27. Inflated signals in batch B and mitigation by PC1 adjustment. An unusually large number of significantly differentially abundant proteins was observed in batch B before PC1 adjustment. The most strongly upregulated protein was PGAM1, a platelet activation marker, suggesting pre-analytical variations. After adjustment for the first PC, inflated signals were substantially reduced. Effect sizes were more consistent across batches. P_{BH}, Benjamini-Hochberg-corrected P value.

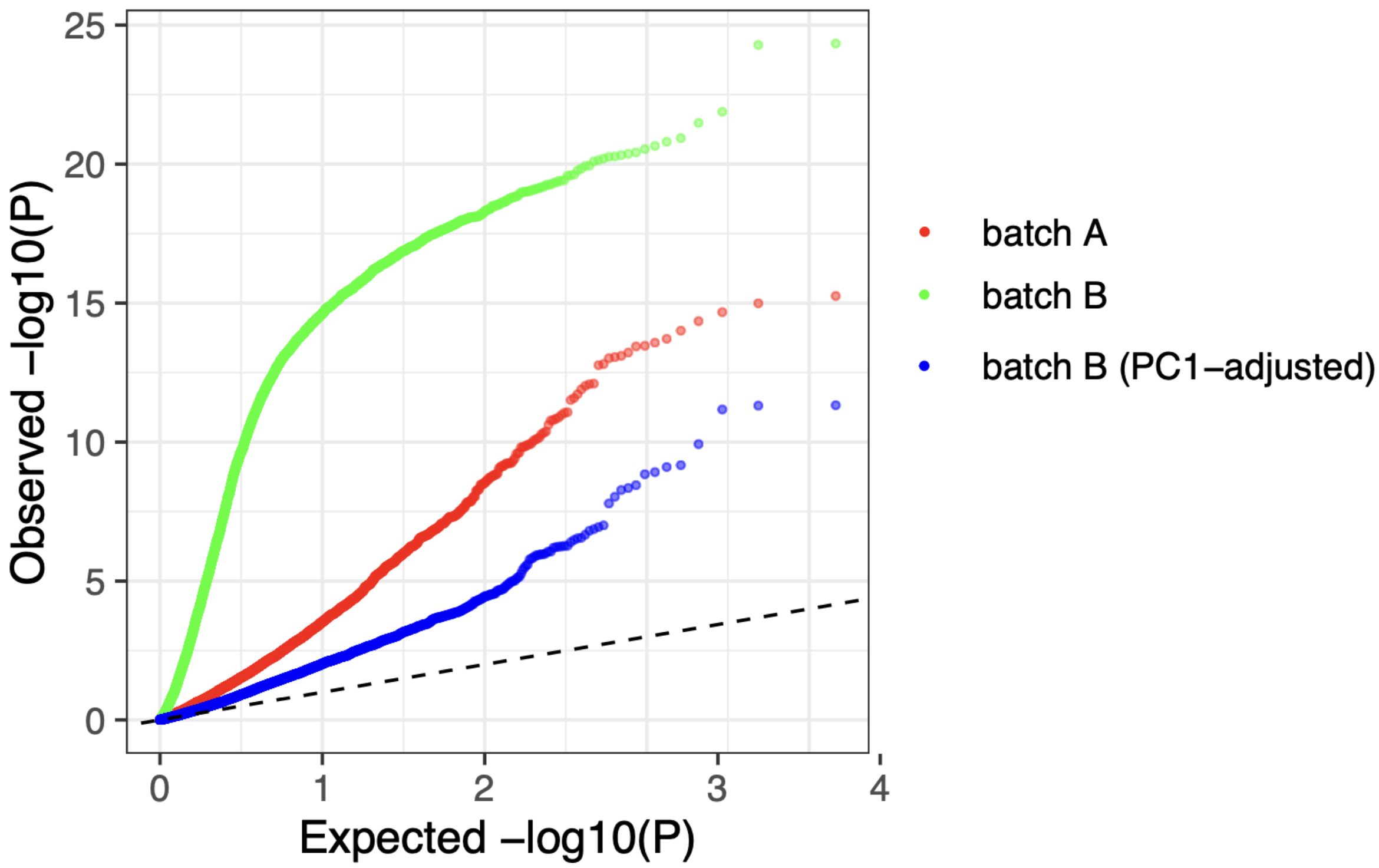

Supplementary Figure 28. QQ plots of SLE vs HV associations. X-axis: expected nominal P values under the null hypothesis of no association between levels of each protein and SLE status. Y-axis: nominal P values from the differential protein abundance analysis. Each point represents a SOMAmer. Batch B showed strongly inflated test statistics (green) relative to batch A (red), which were largely normalised after PC1 adjustment (blue).

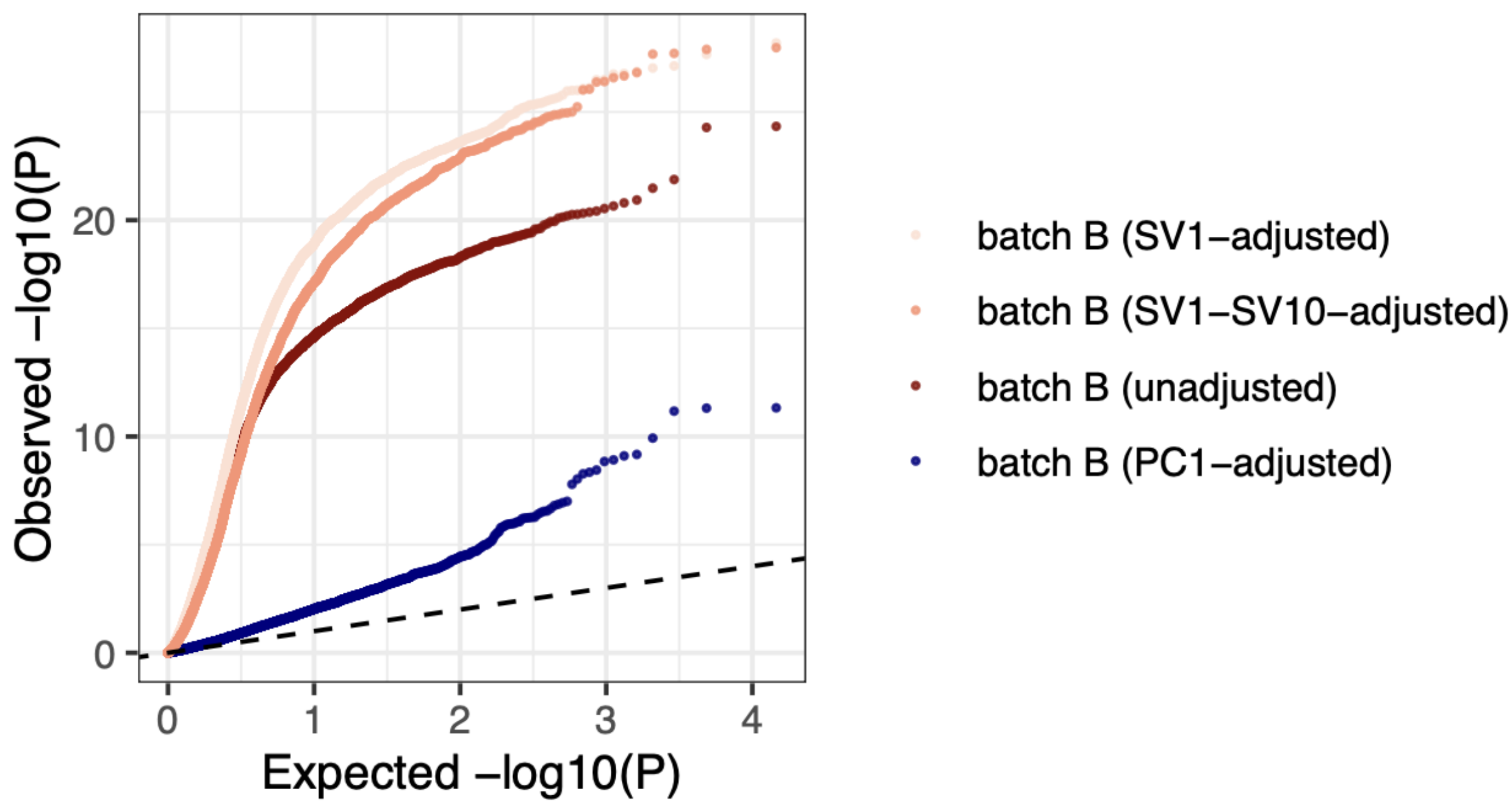

Supplementary Figure 29. QQ plots of SLE vs HV associations in Batch B before and after different approaches for correcting pre-analytical variation. X-axis: expected nominal P values under the null hypothesis of no association between levels of each protein and SLE status. Y-axis: nominal P values from the differential protein abundance analysis. Each point represents a SOMAmer. Batch B showed strongly inflated test statistics (brown). Adjustment for latent factors did not correct inflation (pink and orange), whereas inflation was attenuated after PC1 adjustment (blue).

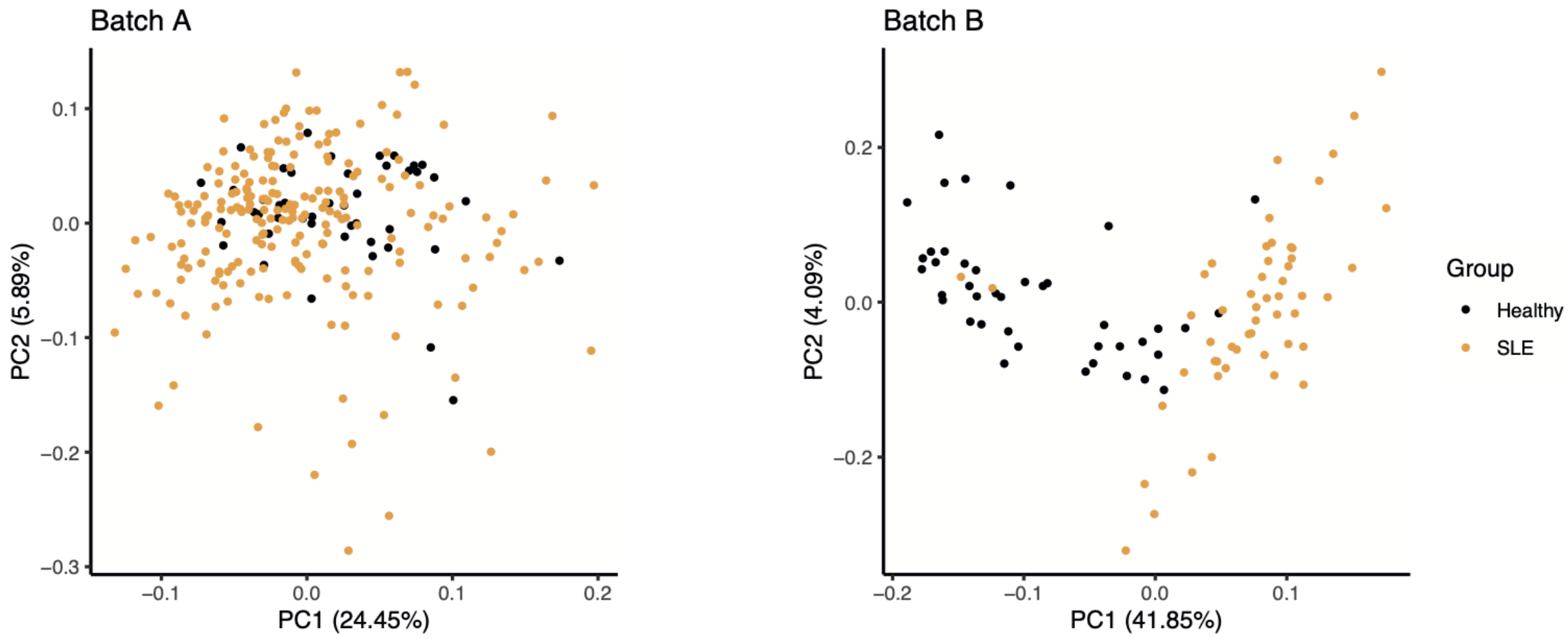

Supplementary Figure 30. PCA of SLE and healthy samples in two batches of data. PCA was performed separately on Batch A and Batch B. Each point represents a sample. Orange = SLE, Black = healthy volunteers

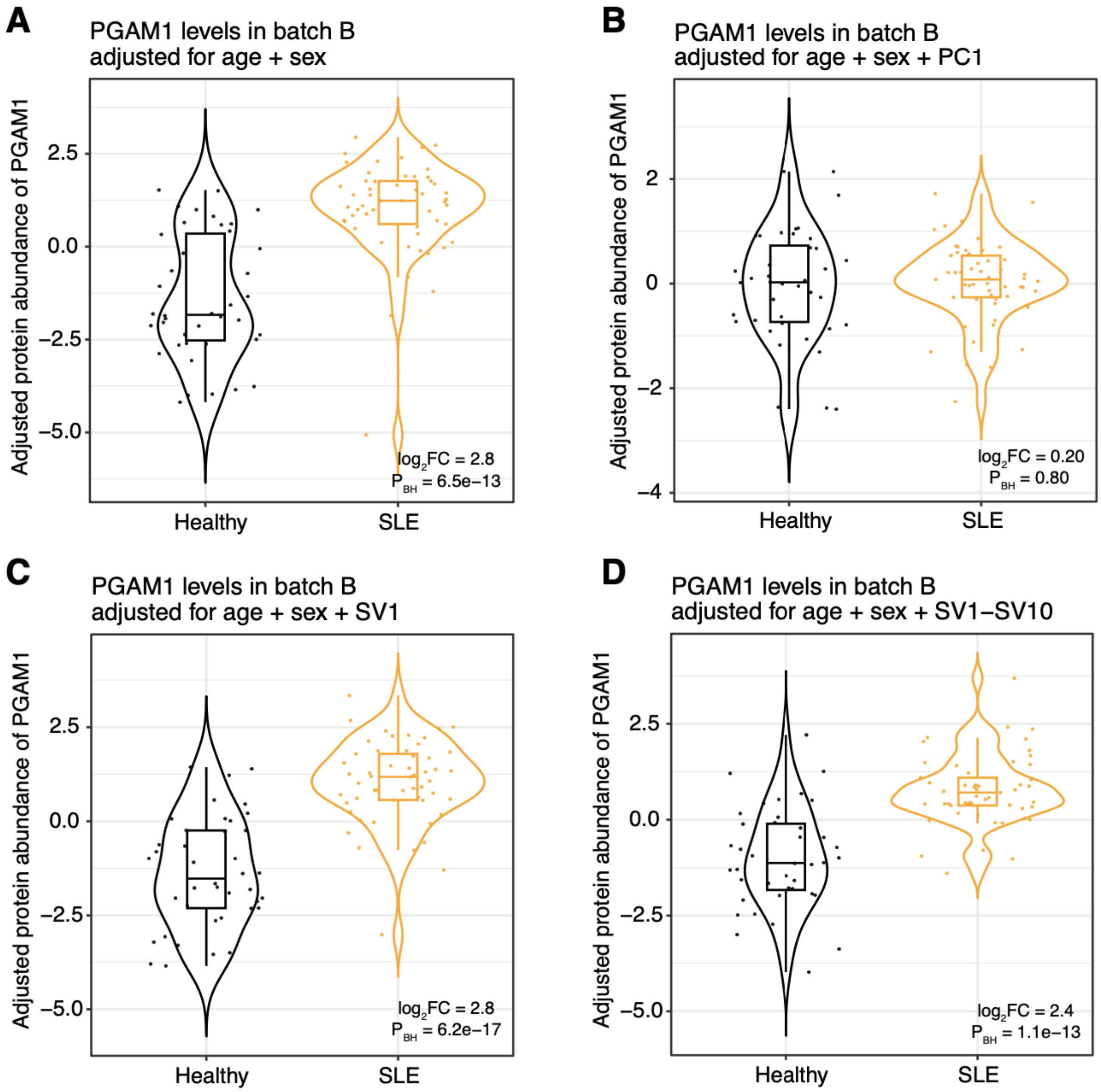

Supplementary Figure 31. Marker of pre-analytical variation in Batch B and comparison of approaches to correct it. A) Association of PGAM1 in SLE vs HVs suggests pre-analytical variation. **B)** Abrogation of PGAM1 association following adjustment for PC1. **C-D)** Adjustment for the first latent factor **(C)** or latent factors 1-10 **(D)** using Surrogate Variable Analysis does not attenuate PGAM1 association.

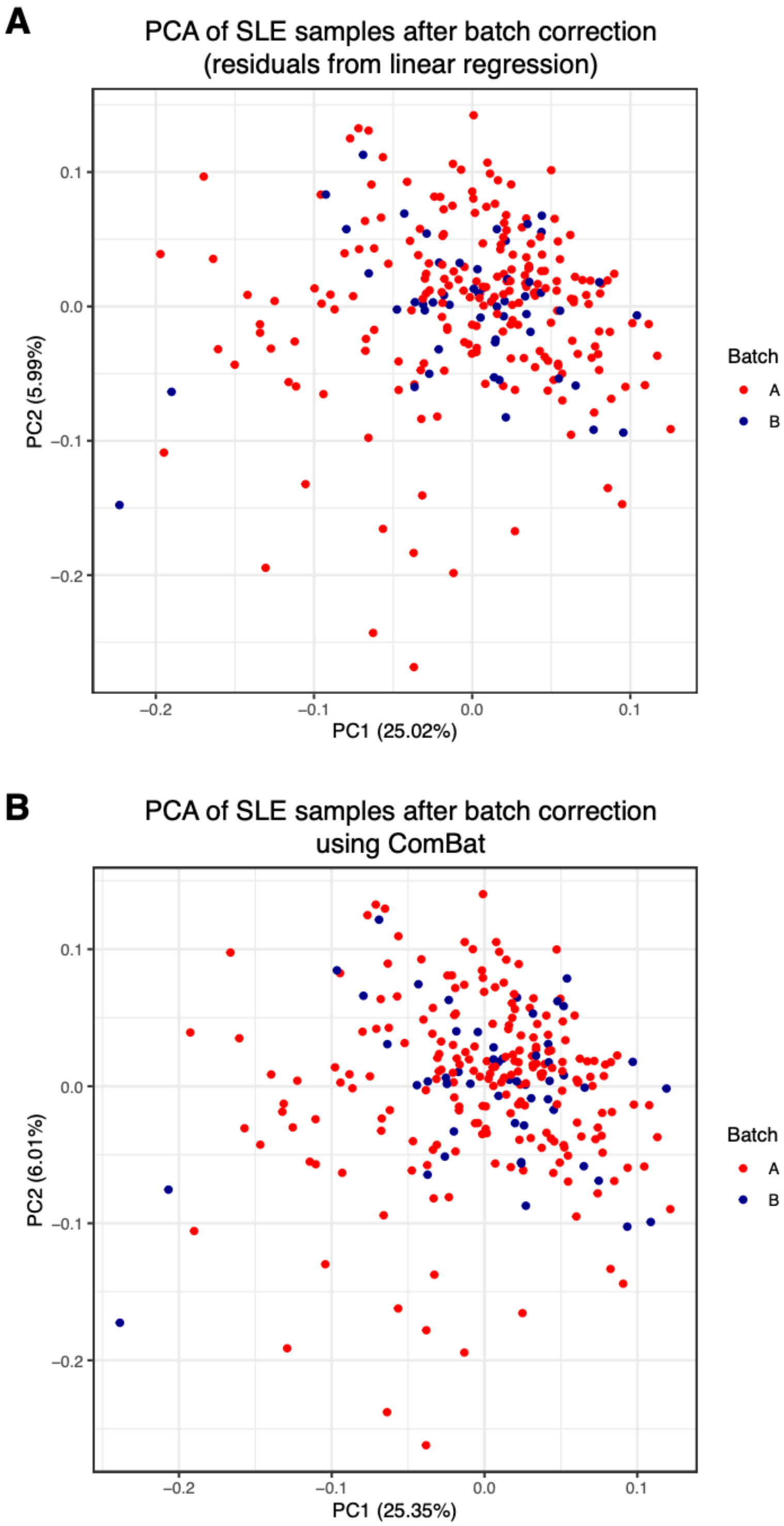

Supplementary Figure 32. Comparisons of batch correction using two different approaches. A. PCA of SLE samples after batch correction using residuals derived from linear regression models. **B.** PCA of SLE samples after batch correction using ComBat.

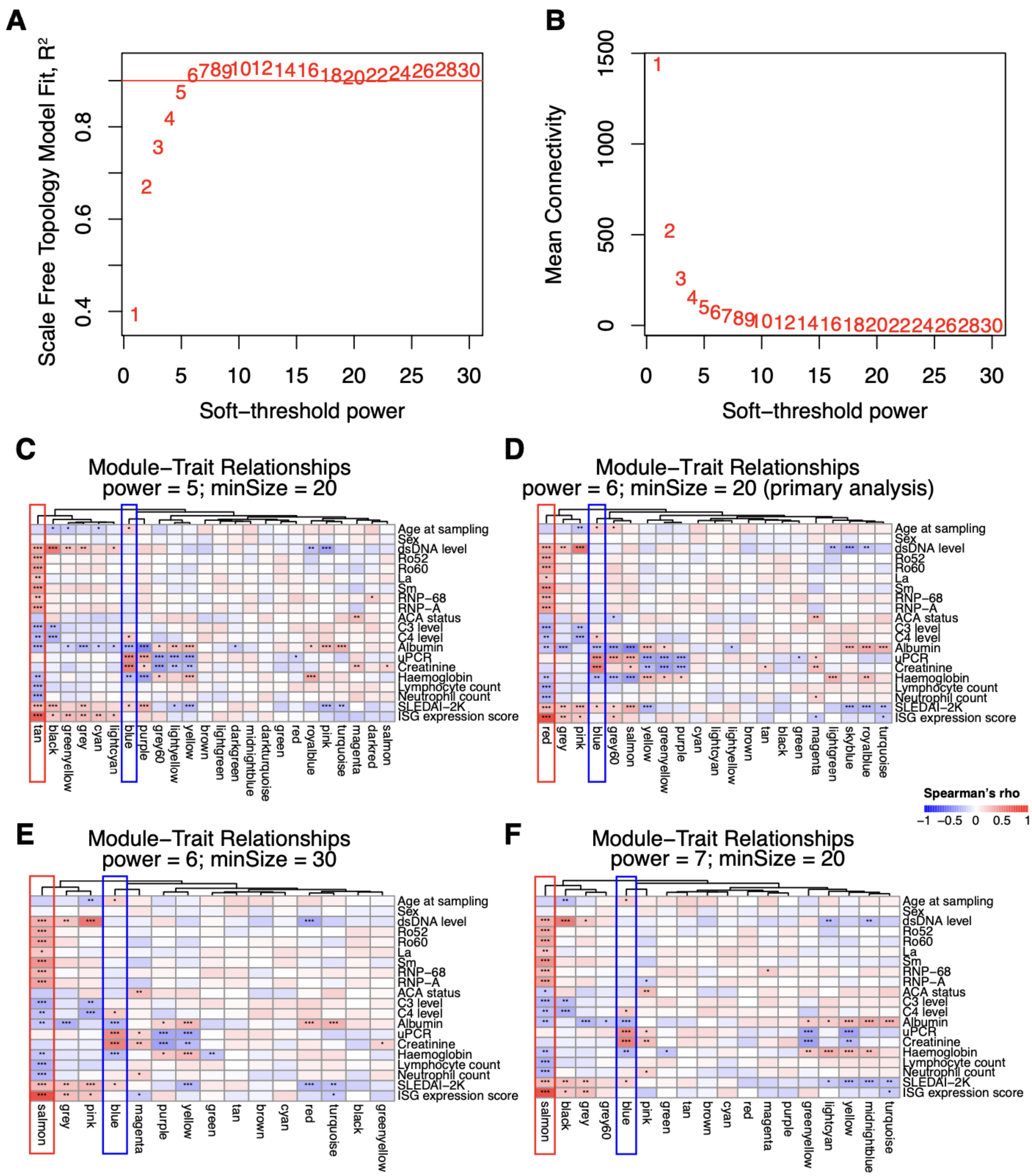

Supplementary Figure 33. Selection of soft-threshold power for WGCNA and sensitivity analyses using different parameters. A) Model fit (R^2) for approximate scale free topology for each soft-threshold power tested. A power of 6 was the lowest power achieving approximate scale free topology (defined as $R^2 > 0.9$, red line). **B)** Mean network connectivity for each soft-threshold power tested. **C-F)** Module-trait relationships by adjusting WGCNA parameters: **C)** power = 5, **D)** primary analysis, **E)** minimum module size = 30, **F)** power = 7. Red box = IFN-associated module. Blue box = renal-associated module. Asterisks represent BH-adjusted P values: * < 0.05, ** < 0.01, ***< 0.001. minSize, minimum module size

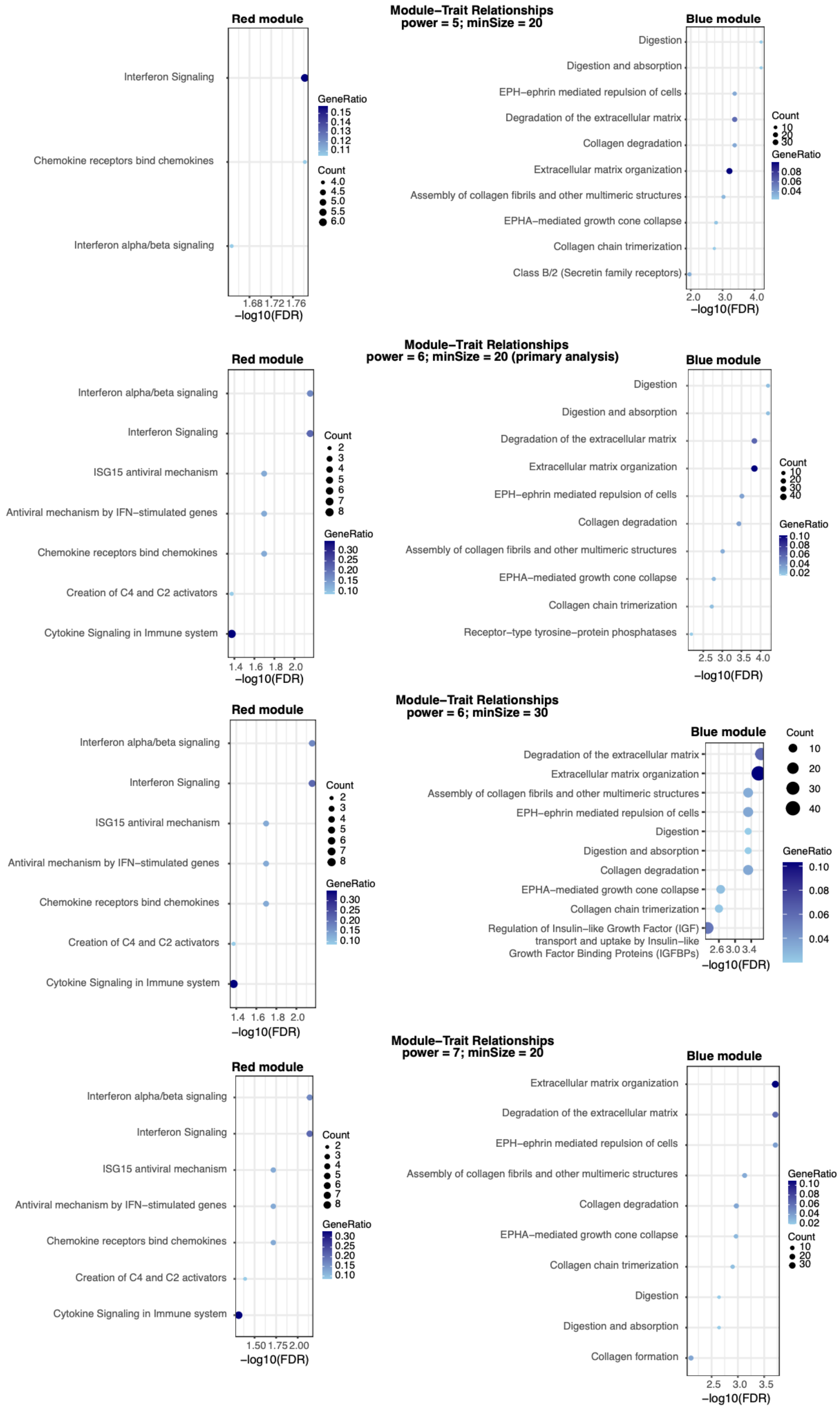

Supplementary Figure 34. Pathways enriched by the red and blue modules identified using different WGNCA parameters. FDR, False discovery rate; minSize, minimum module size

Titles for Supplementary Data (in Excel file)

Supplementary Data 1. Distribution of Lupus Nephritis (LN) classes among SLE patients with previous or recent LN. Contains the number of SLE patients with previous or recent biopsy-confirmed LN in each class.

Supplementary Data 2. All proteins measured by SomaScan in this study. Contains all proteins measured by SomaScan (v4.1) in this study. A total of 7,288 SOMAmers along with their SOMAmer ID, target protein, UniProt ID, Entrez Gene ID, and gene symbol are listed.

Supplementary Data 3. Differential abundance analysis of SLE vs HVs (Batch A). Contains test statistics and estimates from linear regression model comparing SLE vs HVs in Batch A, and protein information, for all 7,288 proteins.

Supplementary Data 4. Differential abundance analysis of SLE vs HVs (Batch B). Contains test statistics and estimates from linear regression model comparing SLE vs HVs in Batch B, and protein information, for all 7,288 proteins.

Supplementary Data 5. SLE vs HVs differentially abundant proteins in both batch A and batch B. Contains test statistics and estimates from linear regression model comparing SLE vs HVs in Batch A and Batch B, and protein information, for 215 validated proteins ($P_{BH} < 0.05$).

Supplementary Data 6. Robust-rank aggregation on pathway terms in SLE vs HVs. Contains Robust-rank aggregation results and GSEA test statistics of pathways in SLE vs HVs comparisons in Batch A and Batch B.

Supplementary Data 7. Summary statistics for each protein from disease activity association tests. Contains test statistics and estimates from linear regression model, and protein information, for all 7,288 proteins tested against disease activity.

Supplementary Data 8. GSEA results of disease activity association analysis. Contains effect sizes, test statistics and leading edge for each pathway term obtained from GSEA tested against disease activity associations.

Supplementary Data 9. Proteins correlated with IFNL1. Contains correlation coefficient and protein information for 75 proteins correlated with IFNL1 (Pearson's $r > 0.6$, $P_{BH} < 0.05$).

Supplementary Data 10. Proteins significantly correlated with clinical C3 levels. Contains correlation coefficient and protein information for 60 proteins correlated with clinically measured C3 levels (|rho| > 0.3, P_{BH} < 0.05).

Supplementary Data 11. Proteins significantly correlated with clinical C4 levels. Contains correlation coefficient and protein information for 62 proteins correlated with clinically measured C4 levels (|rho| > 0.3, P_{BH} < 0.05).

Supplementary Data 12. Connectivity of each protein in the red (IFN-associated) module. Contains connectivity (Spearman's correlation between protein levels and the module's eigenprotein values) and protein information for 35 proteins in the red module.

Supplementary Data 13. Connectivity of each protein in the blue (renal-associated) module. Contains connectivity (Spearman's correlation between protein levels and the module's eigenprotein values) and protein information for 611 proteins in the blue module.

Supplementary Data 14. Proteins associated with anti-Sm positivity before or after adjusting for disease activity. Contains test statistics and estimates from linear regression model comparing anti-Sm(+) vs anti-Sm(-), and protein information, for proteins that are differentially abundant before or after adjusting for disease activity (P_{BH} < 0.05).

Supplementary Data 15. Proteins associated with anti-dsDNA positivity before or after adjusting for disease activity. Contains test statistics and estimates from linear regression model comparing anti-dsDNA(+) vs anti-dsDNA(-), and protein information, for proteins that are differentially abundant before or after adjusting for disease activity (P_{BH} < 0.05).

<u>Supplementary References</u>

1. Johnson, W.E., C. Li, and A. Rabinovic, *Adjusting batch effects in microarray expression data using empirical Bayes methods.* Biostatistics, 2007. **8**(1): p. 118-27.
2. Bakdash, J.Z. and L.R. Marusich, *Repeated Measures Correlation.* Front Psychol, 2017. **8**: p. 456.
3. Elgueta, R., et al., *Molecular mechanism and function of CD40/CD40L engagement in the immune system.* Immunol Rev, 2009. **229**(1): p. 152-72.
4. Langfelder, P. and S. Horvath, *WGCNA: an R package for weighted correlation network analysis.* BMC Bioinformatics, 2008. **9**: p. 559.
5. Shannon, P., et al., *Cytoscape: a software environment for integrated models of biomolecular interaction networks.* Genome Res, 2003. **13**(11): p. 2498-504.

6. Rusinova, I., et al., *Interferome v2.0: an updated database of annotated interferon-regulated genes.* Nucleic Acids Res, 2013. **41**(Database issue): p. D1040-6.
7. Buang, N., et al., *Type I interferons affect the metabolic fitness of CD8(+) T cells from patients with systemic lupus erythematosus.* Nat Commun, 2021. **12**(1): p. 1980.
